# 4273*π*: Bioinformatics education on low cost ARM hardware

**DOI:** 10.1186/1471-2105-14-243

**Published:** 2013-08-12

**Authors:** Daniel Barker, David EK Ferrier, Peter WH Holland, John BO Mitchell, Heleen Plaisier, Michael G Ritchie, Steven D Smart

**Affiliations:** 1 Sir Harold Mitchell Building, School of Biology, University of St Andrews, St Andrews, Fife KY16 9TH, UK; 2 Scottish Oceans Institute, School of Biology, University of St Andrews, East Sands, St Andrews, Fife KY16 8LB, UK; 3 Department of Zoology, University of Oxford, South Parks Road, Oxford OX1 3PS, UK; 4 Biomedical Sciences Research Complex and EaStCHEM School of Chemistry, Purdie Building, University of St Andrews, North Haugh, St Andrews, Fife KY16 9ST, UK; 5 School of Biology, Bute Building, University of St Andrews, St Andrews, Fife KY16 9TS, UK

**Keywords:** Bioinformatics education, Teaching material, Raspberry Pi, Linux

## Abstract

**Background:**

Teaching bioinformatics at universities is complicated by typical computer classroom settings. As well as running software locally and online, students should gain experience of systems administration. For a future career in biology or bioinformatics, the installation of software is a useful skill. We propose that this may be taught by running the course on GNU/Linux running on inexpensive Raspberry Pi computer hardware, for which students may be granted full administrator access.

**Results:**

We release 4273*π*, an operating system image for Raspberry Pi based on Raspbian Linux. This includes minor customisations for classroom use and includes our Open Access bioinformatics course, *4273π Bioinformatics for Biologists*. This is based on the final-year undergraduate module BL4273, run on Raspberry Pi computers at the University of St Andrews, Semester 1, academic year 2012–2013.

**Conclusions:**

4273*π* is a means to teach bioinformatics, including systems administration tasks, to undergraduates at low cost.

## Background

Bioinformatics is increasingly included in the undergraduate curriculum for biology students. Teaching bioinformatics is made difficult, however, by the constraints of a typical university computer classroom. Some areas of basic bioinformatics may be taught using such classrooms, where all that is required is an Internet connection and Web browser (e.g. BLAST [1] searches at the NCBI [2]). More in-depth teaching requires the re-creation of a bioinformatics research environment, consisting of a Linux or UNIX operating system, standard GNU utilities [3], specialist bioinformatics software, and sequence databases.

Undergraduate modules ought, ideally, to prepare students for research in an academic research group. Students who do pursue a research career will often find that institutional computer support is targeted to generic computer use (e.g. Microsoft software) rather than installing and maintaining systems suitable for bioinformatics. Particularly outside of bioinformatics research (but also occasionally within it), the principal investigator of the research group may never have used Linux, may have a limited idea of the procedures, and may expect group members to ‘pick things up’ and deal with problems themselves. This requires researchers to have a high level of proficiency with Linux, including the ability to install both standard Linux packages and software for which no standard package may be available. A single taught module cannot prepare a student for all eventualities, but ought to leave the student with the basic skills and confidence to be able to discover solutions, and implement them, as required. Hence, a certain amount of system administration should appear in an undergraduate bioinformatics module for biologists.

Traditionally, the environment required for an undergraduate bioinformatics module has been created in one of four ways. Firstly, one may set up a central GNU/Linux server on the campus and allow students to connect by Secure Shell, ssh (‘the server approach’). The server will typically run either a standard Linux distribution or a specialist bioinformatics distribution such as NEBC Bio-Linux [4]. The server approach allows the instructor to have full control over the server, and allows students to connect from existing computing classrooms with little or no adjustment to the classroom software. For students to connect to the server via the intranet, classroom computers only require an ssh client, the X Window System (X11), and a means of file transfer such as secure copy (scp). Students may also connect to the server from home (typically requiring them to install virtual private network software in addition to ssh, X11 and an scp client) or elsewhere on campus. Secondly, one may provide students with a virtual machine, consisting of an environment similar to that which they might experience on the Linux server but running on a classroom computer, either with a standard Linux distribution or a specialist bioinformatics distribution such as DNA Linux Virtual Desktop Edition [5] (‘the VM approach’). This has the advantage that students may be given administrator access to their virtual machine. Thirdly, one may provide students with a Linux system on removable media (‘the USB stick approach’, for example [6]; where files and settings do not have to be saved, a DVD may be used instead [7]). So long as students have the media to hand, this allows them to boot into ‘their own’ Linux. As with the VM approach, students may be given administrator access. The additional advantage is that the media may be portable between computer classrooms and home computers, without requiring students to move virtual machine image files. Fourthly, students may be loaned or required to buy laptops of a specific kind, with a suitable operating system, data and software installed (‘the laptop approach’). This avoids hardware incompatibilities that the USB stick approach may, in practice, experience [6].

Because administrator access cannot be allowed, the server approach fails to give students experience of the standard mechanism of software installation. It also involves competition for resources such as CPU time, especially if the class is large or the server is also shared with research colleagues. The VM approach solves both these problems but is less portable. Although, in theory, students may transfer a VM from one computer to another (assuming the destination has the necessary virtualisation software installed), the task is non-trivial, and more time consuming than a simple transfer of data or documents. The USB stick approach reduces the portability problem, since it is trivial to move a USB stick from one computer to another. However, smooth operation on all hardware is not guaranteed and requires ongoing efforts from the developers of the Linux distribution as new hardware is released. The laptop approach avoids all these problems by providing a portable computer holding everything required for the course. However, it is expensive.

As a fifth approach, we propose loaning a Raspberry Pi computer [8] and associated peripherals to students for the duration of the course (‘the Raspberry Pi approach’). This includes a customised version of Linux, appropriate software and data. This allows students full administrator access to a suitable operating system, without the difficulties of the VM or USB stick approaches. Should the student accidentally damage critical files, the system can be re-written from a master image.

The Raspberry Pi Model B – with 256 MB (now 512 MB) RAM, an ARM11 CPU running at 700 MHz before over clocking and a Video core IV GPU – was released for public sale in 2012 [9] and costs £28.07^a^ or £31.20 [10,11]. Though additional items are required to turn it into a functioning, general-purpose computer (case, charger, SD card, mouse, keyboard, monitor and cable; and an entirely separate computer for initialising the SD card), it is still relatively low-cost (Additional file 1: Table S1). The existence of the Raspberry Pi is partly a celebration of the early days of popular computing in the 1980s, and an attempt to recreate that excitement among young people today [12]. It is also a symptom of the rapidly decreasing costs and increasing performance of computer hardware. The Raspberry Pi uses an ARM CPU [13]. Because of their high performance-per-watt, ARM CPUs are frequently found in small electronic appliances such as mobile phones and tablets. With CPU innovation increasingly driven by such applications, as opposed to more traditional areas such as desktop, laptop and server computers, the prevalence and utility of ARM-based computer hardware is likely to increase. Indeed, ARM-based servers are starting to appear in data centres, due to their modest requirements for power [14].

Though far slower than current desktop and laptop computers, the Raspberry Pi is notably faster than the Cray 1 supercomputer [15], a marvel of computer speed in its day. The valid question arises as to how much computer power is actually required to teach undergraduates bioinformatics? We propose that the answer is, by current standards, ‘not much’. The Raspberry Pi is more than adequate for the task. The Raspberry Pi approach includes all the benefits of the laptop approach, above, but at lower cost. In addition, the Raspberry Pi is a new and exciting computer system, which in itself can add interest to the course.

A variety of operating systems is available for the Raspberry Pi [16]. These include Raspbian [17], which is based on Debian GNU/Linux [18]. Over 35,000 Debian software packages are available pre-compiled for Raspbian, including Web browsers, text editors, word processors, and a wide range of bioinformatics packages [19]. Other software will usually compile and run without problems. Some features of recent CPUs (e.g. 64-bit addressing or vector operations) are absent, but we have not found these to be at all necessary for our proposed use of the Raspberry Pi. The most serious limitation, affecting structure visualisation software in particular, is limited graphics performance. Even so, some structural visualisation software does work on the Raspberry Pi [20]. New system software, improving graphics performance by making better use of the Raspberry Pi’s GPU, is under development [21].

We provide 4273*π*, a customised version of Linux for Raspberry Pi computer hardware. 4273*π* includes an Open Access bioinformatics course, *4273π Bioinformatics for Biologists*.

## Implementation

*4273π Bioinformatics for Biologists* is based on the module BL4273 Bioinformatics for Biologists at the University of St Andrews [22], an optional module of 15 SCOTCAT credits, equivalent to 7.5 ECTS credits or ~4 US credits. BL4273 is intended for final-year undergraduate students on BSc(Hons) Biology, BSc(Hons) Biochemistry and other degree courses taught by the School of Biology. BL4273 was taught on Raspberry Pi hardware in Semester 1 of academic year 2012–2013. During this time, students were loaned a Raspberry Pi, SD card and USB stick for backup. Teaching was carried out in a small computer room in which students connected to monitors more generally used with Windows desktop PCs. Students were allowed to take all equipment home on loan (including keyboard, mouse and cables), with the exception of the monitors; several copies of the main textbook [23] were also available. Course material was released week-by-week using an rsync server (running on a Raspberry Pi) on the university intranet. Following the conclusion of the module, material was edited to remove closed-access material (e.g. images in lectures), typographical errors were corrected, and the SD card image was re-created using a recent version of Raspbian [17].

Preparation of a release of 4273*π* begins with the latest Raspbian SD card image [24]. This is then customised to produce a ‘master’ SD card for the release, partly by a series of scripts which alter the configuration and use Raspbian’s port of the APT mechanism of Debian [18] to install specialist bioinformatics packages [19] and more general packages, and partly by a series of commands entered manually (e.g. to install BLAST databases and *4273π Bioinformatics for Biologists* in the ~/4273pi/ directory). The master SD card image is stored on a separate computer and uploaded to the 4273*π* Web site. A ‘work instruction’ detailing the steps performed to convert Raspbian into 4273*π*, and all scripts used, are distributed with 4273*π*.

For the permanent record, the teaching material included in the current release – excluding Linux, software and BLAST databases – is available as Additional file 2. The latest version may be downloaded from the 4273*π* Web site [25].

As the Raspbian operating system, Raspberry Pi firmware and hardware and *4273π Bioinformatics for Biologists* teaching material develop, further releases of 4273*π* will be made available. It is anticipated that there will be a minimum of two releases per year during the next four years.

## Results and discussion

**Figure 1:**
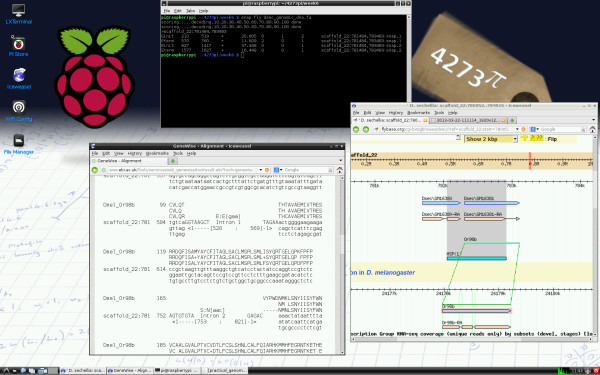
**4273*π *screenshot.** Practical, Week 7. Predicted gene structures of the Or98b gene in the *Drosophila sechellia* genome are being compared. Different predictions are made *de novo* by SNAP [26], run in a terminal on the Raspberry Pi; GeneWise [27], run online at the European Bioinformatics Institute [28], with the *D. melanogaster* protein being aligned against *D. sechellia* genomic DNA; and using TBLASTN [1], run online at FlyBase [29]. FlyBase also displays the annotation of this gene from its database. This case study arose during research for a comparative study of the chemoreceptor superfamily in *Drosophila*[30]. The 4273*π* background desktop image includes hand-written versions of material from *4273π Bioinformatics for Biologists* and from the module BL3320 Practical Statistics for Biologists, which most students at the University of St Andrews would have taken earlier. It also includes a copy of one of the first published phylogenetic trees, from *Vestiges of the Natural History of Creation*, written in St Andrews [31].

4273*π* provides an attractive, general-purpose computing environment, within which the course *4273π Bioinformatics for Biologists* is embedded (Figure 1; Table 1). Student feedback questionnaires for the module BL4273, upon which it is based, are too few to draw strong conclusions (five returned from a class of six students; Additional file 1: Table S2). However, they were sufficiently positive to continue the module without modification.

**Table 1:** **Timetable for 4273***π ***Bioinformatics for Biologists**

**Week**	**Lecture**	**Practical class**
1	Genomes, sequences and bioinformatics data.	Linux and Perl.
2	-	Linux, Perl and protein BLAST.
3	-	Linux, Perl and delimiting gene/protein families.
4	Multiple alignment and phylogeny.	Multiple alignment and phylogeny.
5	Gene family evolution.	Gene family evolution.
6	BLAST; DNA sequence analysis.	DNA sequence analysis.
7	Looking at species differences.	Detecting positive selection.
8	Function and evolution of enzymes.	Function and evolution of enzymes.
9	Seminar: student presentations.	-

Lectures last slightly less than an hour. Practical classes all use the Raspberry Pi, and last two hours. In addition, in their own time, students engage in self-study and complete a coursework ‘practical project’.

## Conclusions

### (Administrator) power to the people

Rather than present systems administration as a complex task to be delegated to a technical support unit – which in students’ future careers may not be available – *4273π Bioinformatics for Biologists* introduces software installation on Linux through standard package management (APT), through compilation (GNU ‘make’ for SNAP [26] and PAML [32]) and through command-line launch of a JAR (Modelgenerator [33] and Mesquite [34]). Other administrative tasks covered include upgrading Linux and installing a MySQL server. Although these matters of system administration are incidental to an intellectual understanding of bioinformatics, they do not take much time to teach, and we believe they leave students well-prepared for a bioinformatics research career – in many cases, better prepared than any other members of the research group within which they are working.

### The virtue of patience

No serious compromises in content were required to teach bioinformatics on the Raspberry Pi. A BLAST search of the GenPept database (‘nr’) is, in practice, too slow. However a BLAST search of the SwissProt database takes only a few minutes. Delimitation of protein families across two prokaryotic genomes using BLAST and OrthoMCL is entirely feasible, and is central to the coursework component of *4273π Bioinformatics for Biologists*. Java (Modelgenerator, Mesquite) programs run slowly, but not unbearably so. Speed will likely improve once Oracle Java becomes available for the platform. Floating-point-intensive tasks (PhyML [35] and PAML) are also slow, but feasible. Waiting an hour for an analysis is, in fact, realistic training for bioinformatics research. Research will tend to use a far faster computer or cluster, but with far larger input. Teaching bioinformatics on rather slow hardware was not universally popular among students (Additional file 1: Table S2). However, it is a valuable lesson in the transferrable virtue of patience, and the value of checking the configuration of analyses before launching them.

### Low-cost teaching and learning

When the undergraduate module BL4273 at the University of St Andrews ran previously, in Semester 1 of academic year 2008–2009, it used the server approach. Students used desktop computers running Windows in a computer classroom to connect to a 64-bit IBM System x3755 8877 server with 4 AMD CPU cores and 16 GB RAM, running Debian Linux. When purchased in 2008, this server cost £19,537 after educational discount, including 20 TB external storage (14 TB usable as RAID5), uninterruptable power supply (UPS), tape library for backup, and 3 years’ hardware support. It was housed in a secure air-conditioned server room, where it required the University’s IT Services department for installation and maintenance (and for setting up the tape library in a different building) and the University’s Estates department to set up a new 15A electrical connection for the UPS. Although this did not happen, there was always the fear of down-time at some crucial stage in teaching, which is more problematic for a single server being used by all students than for (say) one desktop computer in a classroom. As well as teaching, the server was used for research purposes, leading to worries about conflicting resource use between students and researchers.

To use *4273π*, the investment in hardware per set of equipment, including the Raspberry Pi but excluding the monitor, before any quantity or educational discount is ~ £147-£159, depending on what exactly is bought and from where (Additional file 1: Table S1; Background). This is far cheaper than the server used in 2008–2009, but is not extremely cheap. However, these are mostly one-off expenses, since the equipment may be re-used; some components are likely already present in an educational establishment; the maximum cost of any one part is no more than £31.20, allowing cheap repairs compared to ‘the laptop approach’; and the cost of some parts of the equipment (e.g. SD card) continues to fall noticeably. Among its other advantages, the Raspberry Pi approach is a low-cost method for bioinformatics teaching and learning.

### Open learning

By including an explicit Open Access licence, and removing or replacing material incompatible with this from *4273π Bioinformatics for Biologists*, we have been able to share it with anyone interested, the world over, in such a way that they can – with minimal care – re-use and adapt it without accusation of plagiarism or copyright violation. This approach is broadly in common with the pioneering EcoEd Digital Library [36] and related portals [37], but is in contrast to most of the teaching material that can be found by an online search, for which the licence is unclear. We expect our approach will lead to mutual benefits, for example the contribution of corrections or teaching material by others. As Open Access publication is becoming more standard for research, we predict that Open Access will become more standard for teaching material.

## Availability and requirements

**Project name:** 4273*π*

**Project home page:**http://eggg.st-andrews.ac.uk/4273pi

**Operating Systems:** Linux

**Other requirements:** Raspberry Pi computer hardware

**Licence:***4273πBioinformatics for Biologists* has a Creative Commons Attribution licence (http://creativecommons.org/licenses/by/2.0)

**Any restrictions to use by non-academics:** no

## Endnotes

^a^Prices in British Pounds (GBP), including UK tax but excluding delivery charges, obtained on 26 June 2013. £1 converts to approximately $1.54 US Dollars, €1.18 Euro, or R93.04 Indian Rupees [38].

## Abbreviations

AMD: Advanced Micro Devices; APT: A package tool; BSc(Hons): Bachelor of Science with Honours; CPU: Central processing unit; ECTS: European credit transfer and accumulation system; GB: Gigabyte; GNU: GNU’s not UNIX!; GPU: Graphics processing unit; IBM: International Business Machines; IT: Information technology; JAR: Java archive; MHz: Megahertz; NCBI: National Center for Biotechnology Information; NEBC: NERC Environmental Bioinformatics Centre; NERC: Natural Environment Research Council; PC: Personal computer; RAID5: Redundant array of independent disks, level 5; RAM: Random-access memory; SCOTCAT: Scottish Credit Accumulation and Transfer; scp: Secure copy; SD: Secure digital; ssh: secure shell; TB: Terabyte; USB: Universal serial bus; UPS: Uninterruptable power supply; US: United States; VM: Virtual machine; X11: The X Window System.

## Competing interests

The authors declare that they have no competing interests.

## Authors’ contributions

DB conceived the study, and wrote the manuscript with contributions from other authors. DB, DEKF, PWHH, JBOM and MGR designed and wrote teaching material, which was edited by DB and HP. DB configured Raspbian and prepared the 4273*π* release, with contributions from SDS and HP. All authors read and approved the final manuscript.

## Supplementary Material

Additional file 1: Table S1 Example prices of Raspberry Pi peripherals we found to work well in practice. These are presented without any endorsement. A case for the Raspberry Pi (various models and suppliers; ~£5-£10), the Raspberry Pi itself (see main text) and a monitor are not shown. Standard consumer prices, including UK tax but excluding any delivery charge, were obtained from the Insight UK Web site (http://uk.insight.com) or via the Amazon UK Web site (http://www.amazon.co.uk) on 7 April 2013. Click here for file

Additional file 2 ***4273π Bioinformatics for Biologists *** **teaching material, Version 1.01.** The module handbook, lectures and practicals are included. The latest version, including Linux, software and BLAST databases, is available at the *4273π* Web site [25]. Click here for file

## References

[B1] Altschul SF Madden TL Schäffer AA Zhang J Zhang Z Miller W Lipman DJ Gapped BLAST and PSI-BLAST: a new generation of protein database search programs Nucleic Acids Res 1997 25 3389 3402 10.1093/nar/25.17.3389 9254694 PMC146917

[B2] NCBI BLAST home http://blast.ncbi.nlm.nih.gov/Blast.cgi

[B3] GNU operating systemhttp://www.gnu.org

[B4] Field D Tiwari B Booth T Houten S Swan D Bertrand N Thurston M Open software for biologists: from famine to feast Nat Biotechnol 2006 24 801 803 10.1038/nbt0706-801 16841067

[B5] Bassi S Gonzalez VC DNALinux virtual desktop edition Nature Precedings 2007 http://dx.doi.org/10.1038/npre.2007.670.1

[B6] Bio-Linux 7 USB memory stickshttp://nebc.nerc.ac.uk/tools/bio-linux/live-usbkey

[B7] Yu G Wang LG Meng XH He QY LXtoo: an integrated live Linux distribution for the bioinformatics community BMC Res Notes 2012 5 360 10.1186/1756-0500-5-360 22813356 PMC3461469

[B8] Raspberry Pi: An ARM GNU/Linux box for $25 http://www.raspberrypi.org

[B9] BBC News: The Raspberry Pi computer goes on general salehttp://www.bbc.co.uk/news/technology-17190918

[B10] Element14 Community: Raspberry Pihttp://www.element14.com/community/groups/raspberry-pi

[B11] RS Components: Raspberry Pi http://uk.rs-online.com/web/generalDisplay.html?id=raspberrypi

[B12] Roberts J Is the Raspberry Pi the future of computing? techradar.pro, from Linux Format Issue 156 2012 http://www.techradar.com/news/computing/pc/is-the-raspberry-pi-the-future-of-computing-1078276

[B13] ARM: The architecture for the digital worldhttp://www.arm.com

[B14] Latif L ARM sees its 32-bit chips being deployed in future servers: not everything needs 64-bit addressing. The Inquirer 2013 http://www.theinquirer.net/inquirer/news/2259386/arm-sees-its-32bit-chips-being-deployed-in-future-servers

[B15] 2000 Nickels: A Cray for $35 http://2000nickels.com/blog/2012/11/19/a-cray-for-35-dollars

[B16] Raspberry Pi: Operating system distributions http://www.raspberrypi.org/phpBB3/viewforum.php?f=18

[B17] Raspbianhttp://www.raspbian.org

[B18] Debianhttp://www.debian.org

[B19] Möller S Krabbenhöft HN Tille A Paleino D Williams A Wolstencroft K Goble C Holland R Belhachemi D Plessy C Community-driven computational biology with Debian Linux BMC Bioinformatics 2010 11 Suppl 12 S5 10.1186/1471-2105-11-S12-S5 21210984 PMC3040531

[B20] O’Boyle NM Noel O’Blog: Chemistrify your Raspberry Pi Part IIIhttp://baoilleach.blogspot.co.uk/2013/01/chemistrify-your-raspberry-pi-part-iii_19.html23767927

[B21] Raspberry Pi: Wayland previewhttp://www.raspberrypi.org/archives/4053

[B22] University of St Andrews: Undergraduate course catalogue 2012–2013 http://www.st-andrews.ac.uk/coursecatalogue/ug/2012-2013

[B23] Bradnam K Korf I Unix and Perl to the rescue! a field guide for the life sciences (and other data-rich pursuits) 2012 Cambridge: Cambridge University Press

[B24] Raspberry Pi: Downloadshttp://www.raspberrypi.org/downloads

[B25] 4273πhttp://eggg.st-andrews.ac.uk/4273pi4273

[B26] Korf I Gene finding in novel genomes BMC Bioinformatics 2004 5 59 10.1186/1471-2105-5-59 15144565 PMC421630

[B27] Birney E Clamp M Durbin R GeneWise and Genomewise Genome Res 2004 14 988 995 10.1101/gr.1865504 15123596 PMC479130

[B28] EMBL-EBI: GeneWise input form http://www.ebi.ac.uk/Tools/psa/genewise

[B29] Marygold SJ Leyland PC Seal RL Goodman JL Thurmond J Strelets VB Wilson RJ the FlyBase Consortium FlyBase: improvements to the bibliography Nucleic Acids Res 2013 41 D751 D757 10.1093/nar/gks1024 23125371 PMC3531214

[B30] Gardiner A Barker D Butlin RK Jordan WC Ritchie MG *Drosophila* chemoreceptor gene evolution: selection, specialization and genome size Mol Ecol 2008 17 1648 1657 10.1111/j.1365-294X.2008.03713.x 18371013

[B31] Chambers R Vestiges of the natural history of creation 1844 London: John Churchill

[B32] Yang Z PAML 4: Phylogenetic analysis by maximum likelihood Mol Biol Evol 2007 24 1586 1591 10.1093/molbev/msm088 17483113

[B33] Keane TM Creevey CJ Pentony MP Naughton TJ Mclnerney JO Assessment of methods for amino acid matrix selection and their use on empirical data shows that ad hoc assumptions for choice of matrix are not justified BMC Evol Biol 2006 6 29 10.1186/1471-2148-6-29 16563161 PMC1435933

[B34] Maddison WP Maddison DR Mesquite: a modular system for evolutionary analysis. Version 2.75 2011 http://mesquiteproject.org

[B35] Guindon S Dufayard JF Lefort V Anisimova M Hordijk W Gascuel O New algorithms and methods to estimate maximum-likelihood phylogenies: assessing the performance of PhyML 3.0 Syst Biol 2010 59 307 321 10.1093/sysbio/syq010 20525638

[B36] EcoEd digital libraryhttp://ecoed.esa.org

[B37] EvoEd digital library: DRD partners and people http://evoed.evolutionsociety.org/index.php?P=DRD_People

[B38] XE Currency converterhttp://www.xe.com/currencyconverter

