# Supplementary material for: 4273π: Bioinformatics education on low cost ARM hardware
Source: BMC Bioinformatics. 2013 Aug 12;14:243. doi: 10.1186/1471-2105-14-243 (PMC3751261; doi:10.1186/1471-2105-14-243)
Supplement: Additional file 1: Table S1 — Example prices of Raspberry Pi peripherals we found to work well in practice. These are presented without any endorsement. A case for the Raspberry Pi (various models and suppliers; ~£5-£10), the Raspberry Pi itself (see main text) and a monitor are not shown. Standard consumer prices, including UK tax but excluding any delivery charge, were obtained from the Insight UK Web site (http://uk.insight.com) or via the Amazon UK Web site (http://www.amazon.co.uk) on 7 April 2013. [file 1471-2105-14-243-S1.pdf]

4273π: Bioinformatics Education on low cost ARM hardware – Additional File 1

Daniel Barker, David E.K. Ferrier, Peter W.H. Holland, John B.O. Mitchell, Heleen Plaisier, Michael G. Ritchie and Steven D. Smart

Table S1. Example prices of Raspberry Pi peripherals we found to work well in practice. These are presented without any endorsement. A case for the Raspberry Pi (various models and suppliers; ~£5-£10), the Raspberry Pi itself (see main text) and a monitor are not shown. Standard consumer prices, including UK tax but excluding any delivery charge, were obtained from the Insight UK Web site (http://uk.insight.com) or via the Amazon UK Web site (http://www.amazon.co.uk) on 7 April 2013.

**Item**	**Cost**	**Supplier and code**
StarTech.com Micro USB cable	£3.59	Insight SRKAA06U90
Microsoft Wired Desktop 600 keyboard	£18.38	Amazon B001QSZ8BG
Logitech B110 optical mouse	£8.39	Insight LTJA06IM6
Belkin Hi-Speed USB 2.0 4-Port mobile hub[1]	£21.59	Insight F5U404PERBLK
CostMad 2A 5V USB charger	£5.99	Amazon B005NZKTRG
Sandisk 32GB 45MB/S SD Extreme Video memory card	£26.99	Amazon B004Q3C98S
Videk 2m HDMI to DVI cable[2]	£7.19	Insight SRKAA058OA
Kingston DataTraveler 100 G2 - USB flash drive - 32 GB	£20.39	Insight KTGA07H0T

[1]For connection to the UK mains, also requires a continental European 2-pin to UK three-pin mains adapter (various models and suppliers; ~£1-£5).
[2]For a monitor with a DVI-d socket. Alternative cables are required for monitors with VGA or HDMI sockets (various models and suppliers).

Table S2. Anonymous student feedback on BL4273 at the University of St Andrews, Semester 1, academic year 2012-2013. Feedback forms were handed out and returned at the final session before the exam. Free-format sections from all forms returned are transcribed below. Where the student entered no response to the question, the cell is left blank. Students are numbered arbitrarily, but consistently throughout the table.

**Student**	**Suggest up to three aspects of this module that you found especially rewarding.**	**Could this module be improved and if so, how?**	**If this module included tutorials and/or seminars, how helpful were these to your understanding of the subject and your motivation?**	**Please add any general comments you would like to make about the module, e.g. rate at which material was delivered, use of visual aids, methods of assessment etc.**
1				
2	- I liked the broad coverage of bioinformatics as opposed to something a bit more specialized.<br>- The practical handouts were very clear and well-written.<br>- It was just a really fun module overall - v. glad I took it.	I was a bit frustrated by how slow internet browsing was on the Raspberry Pi. I think it was quite cool to use it though, but if I had to complain about something it would be that. Also, I would have liked to make more use of textbooks.	N/A? The post-project seminar we are about to have seems like a great idea though.	- Lectures could have been faster, practicals could have possibly been slower with more explanation at each step.<br>- Excellent module overall.
3	• learning practical skills<br>• doing a real mini-research project.<br>• relates to my Honours project!	• use of another system than Raspberry Pi, or in addition to Raspberry Pi.	N/A.	• Raspberry Pi are annoyingly slow for specific things like browsing internet. Use of another system to do practicals would have been good.<br>• Overall great module.
4	The Raspberry Pis worked well.	A little more coverage of the Perl programming prior to the project might be helpful, but it was good that the solutions to the project goals weren't spoonfed to us.		
5	Fantastic idea<br>Current and up to date<br>Highly relevant to evolutionary biology	Teach people Perl more	All seminars + practicals were very useful + Gripping however more perl could have been taught as it was integral to CA [continuous assessment].	Bioinformatics is great and shouldn't be limited to one small module<br>I think that it is really the hot topic in evolutionary biology
