# Supplementary material for: 4273π: Bioinformatics education on low cost ARM hardware
Source: BMC Bioinformatics. 2013 Aug 12;14:243. doi: 10.1186/1471-2105-14-243 (PMC3751261; doi:10.1186/1471-2105-14-243)
Supplement: Additional file 2 — 4273π Bioinformatics for Biologists teaching material, Version 1.01. The module handbook, lectures and practicals are included. The latest version, including Linux, software and BLAST databases, is available at the 4273π Web site [25]. [file 1471-2105-14-243-S2.zip › 4273pi_course_material/week2/practical_linux_perl_blast.pdf]

4273π Bioinformatics for Biologists

Practical, Week 2: Linux, Perl and protein BLAST

Daniel Barker, School of Biology, University of St Andrews
Email db60@st-andrews.ac.uk

© 2013 D. Barker. This is an Open Access document distributed under the terms of the Creative Commons Attribution License (http://creativecommons.org/licenses/by/2.0), which permits unrestricted use, distribution, and reproduction in any medium, provided the original work is properly cited.

4273π, Version 1.01. http://eggg.st-andrews.ac.uk/4273pi

The learning objectives of this practical are to:

- consolidate your knowledge of Linux on the Raspberry Pi;
- introduce the `rsync` command for file transfer and backup; and
- extend your knowledge of running BLAST at the command-line.

At the end of the practical, you should be confident in constructing your own command-lines, installing Raspbian Linux software packages, and running BLAST searches with custom databases and queries. You should also be able to back up your work from the Raspberry Pi to a USB stick.

Raspbian Linux and software packages

Updating and configuring Rasbpian Linux

Raspbian (and Debian) Linux use a tool called APT to manage software packages. Since 4273π is closely based on Raspbian, it uses the same mechanism. The two crucial commands APT provides are `apt-get` and `apt-cache`.

First, use `apt-get` to update the list of software packages your computer knows about. Make sure the Raspberry Pi can reach the Internet, start a new LXTerminal and enter this:

```
sudo apt-get update
```

The `apt-get` program will automatically find the latest list of packages in the default location in which Raspbian packages are made available online, and download it.

Then, install configuration software specific to the Raspberry Pi:

```
sudo apt-get install raspi-config
```

(If the software is already present, this command will do no harm.)

Now, upgrade all the Raspbian packages that are currently installed:

```
sudo apt-get upgrade
```

If you are asked whether you want to continue, check that the specific question is reasonable. If so, press `y` followed by ENTER.

Let's increase the speed at which the Raspberry Pi runs its CPU and graphics hardware (GPU). Enter this command:

```
sudo raspi-config
```

Use the arrow keys to highlight 'overclock', then press ENTER. Read the warning and press ENTER to select the only option ('<OK>'). Use the arrow keys and ENTER to choose 'Modest' overclock, then press ENTER again to select '<OK>'. Finally, in the main menu, use TAB to select '<Finish>'. When asked 'Would you like to reboot now', answer 'Yes'. The Raspberry Pi will re-start. Its CPU and GPU will now run at up to 800 MHz and 300 MHz, respectively – modest increases upon the defaults (700 MHz and 250 MHz, respectively). More extreme settings are possible but may not work well. For example, in my experience 'Turbo' is fast but will cause the Raspberry Pi to crash now and then – a bad idea!

Finding and installing software packages

Later in the module, we will require MySQL database software. This comes in two parts, 'client' and 'server'. To install the MySQL server, do:

```
sudo apt-get install mysql-server
```

MySQL maintains its own password system. It will be easier to remember the password if we use the same password for MySQL and for Linux. When asked for the MySQL root password, set it to:

```
4273pi
```

To install the MySQL client, do:

```
sudo apt-get install mysql-client
```

We will also require software implementing the 'Markov cluster algorithm', MCL. In LXTerminal, use `apt-cache search` to look for a suitable package:

```
apt-cache search mcl
```

You will see there is a package for the MCL software and another package for its documentation. Install these two pacakges using `apt-get`, along the same lines as above.

System load

You will have noticed a green graph on the bottom-right of the screen, indicating how busy the CPU is. This is some help, but does not indicate what is *causing* it to be busy. The program `top` will show the main programs that are running. Start a new LXTerminal and enter this command:

```
top -d 20
```

This will list running 'processes', with those using most CPU time first, and will update every 20 seconds. Leave this running. (Should you want to exit `top`, press `q`.)

Command-line shortcuts

To avoid re-typing a long command at the prompt in LXTerminal, you can use the up and down arrow keys to flick through your recent commands. To run a command again, find it and press ENTER. Or, you can use the left and right arrow keys to move within the command and edit it, before pressing ENTER. CTRL-a will take you to the start of the line, and CTRL-e will take you to the end of the line.

Another approach is to select the text of a previous command with the mouse, right-click and choose 'Copy'. Right-click the LXTerminal where you want to put the text, and choose 'Paste'. It can be helpful to see a list of recently-issued commands, which you get by running the following:

```
history
```

A final and extremely useful tip: to avoid typing a long file, directory name or command, you can press TAB and it will be completed automatically. For example, type the following and then press TAB:

```
cd ~/42
```

This will automatically be expanded to

```
cd ~/4273pi/
```

at which point you can press ENTER. If there is any ambiguity, the name will not be completed – but you can press TAB twice, to see a list of the possible completions.

Files and directories

When you run LXTerminal, you are interacting with a program, known as the 'shell'. The shell has the concept of a 'working directory'. A directory is just a folder in the file system. The working directory is where the shell will look, by default, for files. You can list the files in the working directory as follows:

```
ls
```

When you start LXTerminal, your working directory is `/home/pi`. This is your ‘home directory’ and has an abbreviation, `~`.

`cd` changes the working directory. Without any further parameters (‘arguments’), `cd` will change to your home directory. On the Raspberry Pi, the following commands are all the same:

```
cd /home/pi
cd /home/pi/
cd ~
cd ~/
cd
```

Use `cd` and `ls` to navigate the contents of your home directory and the directories it contains. Use File Manager to verify that you are seeing exactly the files you should.

Manual pages

Linux provides a succinct outline of each major command. These are known as ‘manual pages’, and are shown with the `man` command. Take a look at the manual page for a few commands, e.g.

```
man ls
man cd
man apt-get
man man
```

To navigate within a manual page, SPACE takes you down one ‘page’ and the minus sign takes you back one ‘page’. Press `q` to quit. You can search for a word using forward-slash. For example, do:

```
man shutdown
/-h
```

and press ENTER. This will take you to the part of the manual page where the `-h` command-line option is mentioned.

Environment variables

The shell communicates various default settings to programs, using environment variables.

For example, last week you ran `blastall`, and asked it to search the `swissprot` database. How did the `blastall` program know where to find this database? The answer is, it looked at the `BLASTDB` environment variable.

You do not have to change environment variables at this stage, but it can be helpful to look at their values. Try these commands:

```
echo value of BLASTDB environment variable is: $BLASTDB
echo $HOME
echo $PATH
```

Question. What are the `PATH` and `PERL5LIB` environment variables used for? Search the Web to find out.

Forcing programs to quit

Occasionally you will start a program and then want to stop it prematurely. E.g. perhaps you realise you started a BLAST search with the wrong query sequence, and there is no point in waiting for it to finish.

If running a program at the command-prompt, if it has not completed already, you may force it to quit by pressing CTRL-C.

A less direct way to force a program to quit is to use the `kill` command. Look up the manual pages for `kill` and for `ps`.

As an exercise, experiment with CTRL-C and `kill` to force `top` to quit. (Be careful not to kill other processes – they might be doing something important!)

`rsync` – creating backups

It is always important to back up your work, but even more so with the Raspberry Pi. The SD card, used on the Raspberry Pi instead of a hard disk, is very fragile. It is crucial that you back up your files regularly. Loss of files, due to inadequate backing up, will not be accepted as a reason for late coursework.

Insert a 32 GB USB stick into the powered USB hub (not directly into the Raspberry Pi). If the USB stick is manufactured by Kingston, it appears on the Raspberry Pi file system as `/media/KINGSTON`. For other manufacturers, it will still be found in `/media/`, but the name will be different. Adapt the following instructions accordingly.

If the USB stick does not appear in `/media`, restart the Raspberry Pi. It should then appear.

The `rsync` command creates a 'mirror' of a directory, either from one computer system to another, or between directories on one system. We will be using it to mirror your home directory from the SD card to the USB stick.

Create a complete backup of your home directory on the USB stick, as follows:

```
rsync -rav --delete ~/ /media/KINGSTON/pi/
```

The options `-rav` mean, copy sub-directories (`r`); preserve as much information on files as possible (`a`); and run 'verbosely', displaying actions on-screen (`v`). `--delete` option means, delete any files in the designation which are not present in the source. (This makes no difference the first time; but for subsequent back-ups, will ensure that files you have recently deleted in your home directory will also be deleted in the back-up.) Then, the source directory is given (`~/`); followed by the destination directory (`/media/KINGSTON/pi/`, which will be created if necessary, so long as `/media/KINGSTON/` does exist).

It is crucial to get the source and destination directories the right way around!

You should launch the above `rsync` command now, near the start of the practical. Leave it running in its own LXTerminal.

Run the above `rsync` command again, whenever you have spent time creating new files on the Raspberry Pi. `rsync` detects which files have changed. So, next time, it will copy fewer files and will run much faster.

The USB stick may be ejected via the menu that appears on right-clicking it in File Manager (found under Accessories in the 'Start' menu). You can then insert the USB stick into any desktop or laptop computer running Linux, Windows or Mac OS X. This can be a helpful way to access the module's files on a computer connected to a classroom printer, for example.

As a side-effect of having the USB stick usable among these varying types of operating system, file 'permissions' may be lost. Where you had to do `chmod +` to make a file executable, for example, the backup copy will lose that information. However, the name and contents of the file will be preserved in the backup copy.

When you are not using it to create a new backup, you should keep the USB stick in a safe place, different from where you keep the Raspberry Pi with its SD card.

Genome-wide protein sets and BLAST

Initial examination of genome-wide protein sets

Use `cd` to change to the `~/4273pi/week2` directory. Use `ls` to list the files there. You should see `human_hv_1.fa`, which contains all proteins coded by the genome of human herpesvirus 1 (Herpes simplex virus 1); and `bovine_hv_5.fa`, which contains all proteins coded by the genome of bovine herpesvirus 5. Since they are intended contain all primary products of translation for the genomes, the sequences in these files may be referred to as 'genome-wide protein sets'. In the current case, they were downloaded from the RefSeq database, using Entrez online at the NCBI. They are text files containing sequences in Fasta format.

<u>Question</u>. How many proteins are encoded by the two genomes? First, answer this by looking through the files with `nedit`.

Now, run the following commands in your LXTerminal window:

```
grep '>' human_hv_1.fa
grep '>' bovine_hv_5.fa
```

The `grep` program searches for pieces of text, or 'patterns', within files. It displays all lines which match the pattern. We have asked `grep` to search for >, which is the start of the header line for each protein. So, it will display the Fasta header for each protein – a far smaller quantity of data than the full file. Counting these manually would still be tedious. Of course, this is exactly the kind of job computers ought to do for us. Try these commands:

```
grep '>' human_hv_1.fa | wc -l
grep '>' bovine_hv_5.fa | wc -l
```

Instead of showing matches on the screen, we are now using the output of `grep` as input to another program, `wc`. This is known as 'piping' the output from one program to another, and is achieved with the vertical line or pipe character, `|`. The 'standard output' of the program to the left of the pipe is used as 'standard input' by the program to the right. Pipes are very useful in Linux and UNIX, allowing the user to 'wire together' a series of simple programs to create a complex, customised procedure.

To discover what `wc -l` does, look at the manual page for `wc`:

```
man wc
```

<u>Question</u>. According to `grep` and `wc -l`, how many proteins are encoded by the two genomes? Are these the same counts you came up with by manual counting in `nedit`? If not, whose counts do you believe – yours, or the computer's?

<u>Creating BLAST databases</u>

Although a genome-wide protein set in Fasta format *is* a database in a general sense, BLAST cannot use it as such. First, the sequences must be converted to a format BLAST can use. The necessary command is `formatdb`, and you may run it as follows:

```
formatdb -i human_hv_1.fa
formatdb -i bovine_hv_5.fa
```

Each `formatdb` command will take a few seconds to run. `formatdb` logs a summary of its actions in to a text file named `formatdb.log` in the current directory. Take a look at the contents of `formatdb.log` to confirm that no errors were reported. `formatdb` creates some new files required by BLAST, which you can see if you list files with `ls`. (They have names ending in `.phr`, `.pin` and `.psq`.)

Searching the BLAST databases

To search the bovine herpesvirus 5 database using each human herpesvirus 1 sequence in turn as a query, do:

```
blastall -p blastp -i human_hv_1.fa -d bovine_hv_5.fa
```

As last week, `-p` specifies the BLAST 'program' (`blastp` meaning 'search a protein database with protein query or queries'), `-i` specifies a file in Fasta format holding the query sequence(s), and `-d` specifies the name of the database. Since the BLAST database you made is in the current directory, it is not necessary to change your `BLASTDB` environment variable for BLAST to find it.

The BLAST search results in too much text to see conveniently in LXTerminal. Using the same approach as last week (i.e. with > to redirect the standard output), repeat the BLAST search, but this time, keep the output in a text file. Look at it with `nedit`.

Question. How many matches to human herpesvisus 1 sequences does BLAST find, among the protein set for bovine herpesvirus 5? (Hint: for a rapid, accurate answer, you might like to use a combination of `blastall`'s `-m8` option and `wc -l`.)

Question. For how many distinct human herpesvirus 1 sequences does BLAST find a match, among the protein set for bovine herpesvirus 5? Hint: use the following command:

```
blastall -p blastp -i human_hv_1.fa -d bovine_hv_5.fa -m8 \
| awk '{ print $1 }' | sort | uniq | wc -l
```

Using manual pages and Web searches to find out about `awk`, `sort` and `uniq`, make sure you are happy that you know why this command gives you the right answer.

Question. For how many human herpesvirus 1 sequences does BLAST find no match among the protein set for bovine herpesvirus 5? (Hint: you already have two numbers that you can use – with some straightforward mental arithmetic – to give you the answer.)

It is not extremely useful to count all BLAST matches like this. By default, BLAST reports matches with an E-value (expect value) of up to 10. This E-value threshold is rather high. So, some matches found by the BLAST search may not actually indicate homology.

To report on only the more significant matches found by BLAST, we can make more sophisticated use `awk`. An `awk` program (within the single quotes in the above command) consists of a *condition*, followed by an *action*. The action is the part between braces. Above, we specified *no* condition. By default, with no condition specified, `awk` will perform the action for all input lines.

We will now impose a condition. With the `-m8` option, BLAST outputs the E-value in the 11th field. (You can verify this by running BLAST with the `-m9` option; column or field headings are then listed in order, and you will find that `e-value` is 11th.) So, to report only on the subset of BLAST matches with a low E-value, we may use a condition,

involving the 11th field of BLAST's output. The following will cause `awk` to *only* generate a line of output where the 11th field of its input is less than or equal to 0.05:

```
blastall -p blastp -i human_hv_1.fa -d bovine_hv_5.fa -m8 \
| awk '$11 <= 0.05 { print $1 }' | sort | uniq | wc -l
```

Consequently, only BLAST matches with E-value ≤ 0.05 are counted.

Question. For how many distinct human herpesvirus 1 sequences does BLAST find a match with an E-value ≤ 0.001, among the protein set for bovine herpesvirus 5? Use an `awk` condition to obtain your answer.

As an alternative to using an `awk` condition, we can just tell BLAST to only report the most significant matches in the first place. Find documentation for BLAST's `-e` or 'Expect' command-line option, by means of Web searches and by using the `blastall` command without any options.

Question. For how many distinct human herpesvirus 1 sequences does BLAST find a match with an E-value ≤ 0.001, among the protein set for bovine herpesvirus 5? Instead of an `awk` condition, use BLAST's `-e` option to obtain your answer.

Question. Given that the answers are the same, what are the relative merits of the two approaches to changing the E-value threshold: an `awk` condition, or BLAST's `-e` option?

Now repeat the above BLAST searches, but this time, use the bovine herpesvirus 5 genome-wide protein set as your query file, and the human herpesvirus 1 protein set as your BLAST database.

In some cases, there is a *reciprocal best match* between two proteins (one from each genome). E.g., call the best-scoring bovine herpesvirus 5 protein found, for a human herpesvirus 1 query protein, protein X. Then when protein X is used as a query, in a search of the human herpesvirus 1 protein database, the original human herpesvirus protein is found.

In the current case, for example, if match quality is assessed as bit score, human herpesvirus 1 protein NP_044638.2 and bovine herpesvirus 5 protein YP_003662488.1 are reciprocal best matches between the two protein sets.

Reciprocal best matches are of interest because the sequence has been relatively well conserved between the two genomes. Detecting reciprocal-best matches is one approach to predicting which sequences in the two genomes are *orthologous*. The definition of orthology depends on phylogenetic trees (Fitch 1970, 2000). We have not reconstructed any phylogenetic trees in the current exercise. However, *predictions* of orthology based on BLAST analyses are easier to obtain, and in many cases are not incorrect.

Question. Sketch out the outline of an approach, using BLAST with Linux commands and/or Perl, to automatically identify all reciprocal-best matches between these two genome-wide protein sets. (You do not have to actually write a Perl script or make this approach work. Just think about what specific steps would be involved, and the approaches you might use to achieve them.)

References

Fitch, W.M. (1970) Distinguishing homologous from analogous proteins. *Systematic Zoology* 19: 99-113.

Fitch, W.M. (2000) Homology a personal view on some of the problems. *Trends in Genetics* 16: 227-231.
