# Supplementary material for: 4273π: Bioinformatics education on low cost ARM hardware
Source: BMC Bioinformatics. 2013 Aug 12;14:243. doi: 10.1186/1471-2105-14-243 (PMC3751261; doi:10.1186/1471-2105-14-243)
Supplement: Additional file 2 — 4273π Bioinformatics for Biologists teaching material, Version 1.01. The module handbook, lectures and practicals are included. The latest version, including Linux, software and BLAST databases, is available at the 4273π Web site [25]. [file 1471-2105-14-243-S2.zip › 4273pi_course_material/week3/practical_linux_perl_protfams.pdf]

4273π Bioinformatics for Biologists

Practical, Week 3: Linux, Perl and delimiting gene/protein families

Daniel Barker, School of Biology, University of St Andrews
Email db60@st-andrews.ac.uk

© 2013 D. Barker. This is an Open Access document distributed under the terms of the Creative Commons Attribution License (http://creativecommons.org/licenses/by/2.0), which permits unrestricted use, distribution, and reproduction in any medium, provided the original work is properly cited.

4273π, Version 1.01. http://eggg.st-andrews.ac.uk/4273pi

The learning objectives of this practical are to:

- generally, to gain experience of using existing Perl scripts to perform a research-level analysis in bioinformatics; and
- specifically, to become comfortable with the procedure followed, to use BLAST and OrthoMCL to predict orthologous groups among genomes.

At the end of the practical, you should be able to use BLAST and OrthoMCL to predict orthologous groups for any group of genome-wide protein sets.

Introduction

BLAST is a useful program, but it is not always easy to interpret the output. Often we wish to know, 'does species A contain the same protein-coding gene as species B'? But, at which point do proteins cease to be 'the same'? Proteins are often not identical across species, yet we talk of 'human monoamine oxidase A' and 'cow monoamine oxidase A'. BLAST will give us a score, an E-value and a percentage identity for the match between two proteins, but will not tell us whether they meet this criterion of 'sameness' or not.

What do we even mean by 'the same' gene or protein? Usually we mean orthologous. Orthologous sequences diverged due to a speciation event. This is in contrast to paralogous sequences, which diverged due to duplication within a single genome. Orthology is defined and discussed in two excellent papers by Fitch (1970, 2000). Orthology is one means to divide genes or proteins into 'families' of related sequences.

One way to predict orthology is to use the program OrthoMCL (Li *et al*. 2003).

OrthoMCL is a series of Perl scripts. It processes BLAST output using the MCL package you installed last week, to predict orthologous groups. Each orthologous group contains at least one protein from each species. Thus, OrthoMCL goes beyond the raw output of BLAST, and attempts an answer to the question, 'does species A contain the same protein-coding gene as species B'?

If there is strong evidence that the sequence has duplicated in one or both species since the species diverged, OrthoMCL will also indicate this, by including more than one sequence in a single orthologous group for the species in question. So OrthoMCL also attempts an answer to the question, 'how many copies of the protein-coding gene from species A are contained in the genome of species B'?

Although orthology describes a relationship between genes, in practice we use proteins for the necessary sequence comparisons performed by BLAST. For protein-coding genes, protein sequence comparisons are more sensitive (due to 20 residues, not 4 as with nucleotides) and more accurate (because there is no possibility for BLAST to infer spurious relationships by allowing frameshift indels).

Like most bioinformatics programs, OrthoMCL outputs mere predictions, not absolute truth. There are situations where it will give the wrong answer, both in terms of incorrectly predicting orthology and in terms of incorrectly predicting copy number.

The important concept of orthology is dealt with in greater detail, later in this module. For the moment, please accept that OrthMCL is both imperfect and useful (Chen et al. 2007).

Download software

OrthoMCL is not available as a package you can obtain with `apt-get`. However, it is freely available online. Download and the latest version of OrthoMCL software from:

http://orthomcl.org

Save the file containing the supported version, `orthomclSoftware-v2.0.5.tar.gz` at the time of writing, in the `~/4273pi/week3` directory. Also download the OrthoMCL User Guide, `UserGuide.txt`.

Procedure

Use `cd` to change into the `~/4273pi/week3` directory.

Open the OrthoMCL User Guide, `Userguide.txt`, in `nedit`. Read this carefully. Then go back to the start and work through 'Steps in detail', in order. Follow the

instructions in the User Guide. Some additional notes, to be used in conjunction with the User Guide, are below.

At every step, where you have to run an OrthoMCL command (these all begin with `orthomcl`), you can enter the command without further command-line arguments. This will show some brief, but often crucial, instructions on how to use the command 'for real'. After launching commands, carefully examine output to the LXTerminal window and any log files, for evidence of problems.

Step 1

Skip this step. You installed MySQL last week.

Step 2

Skip this step. You installed the `mcl` package last week.

Step 3

The command to unpack the software is actually:

```
tar xzvf orthomclSoftware-v2.0.5.tar.gz
```

And this gives the directory `orthomclSoftware-v2.0.5`.

The directory containing `orthomcl.config.template`, which you will copy (as `orthomcl.config`) and edit as described in the User Guide, is actually:

```
orthomclSoftware-v2.0.5/doc/OrthoMCLEngine/Main/
```

To create a MySQL database named 'orthomcl', firstly, start the MySQL client:

```
mysql -u root -p4273pi
```

Secondly, create the database by entering this command at the MySQL client's prompt:

```
create database orthomcl;
```

And finally, press CTRL-D to exit the MySQL client. Should you wish to delete the database at some later stage, the (irreversible!) command to enter, in the MySQL client, would be:

```
drop database orthomcl;
```

In `orthomcl.config`, three lines require changing, so that they end up looking like this:

```
dbConnectString=dbi:mysql:orthomcl
dbLogin=root
dbPassword=4273pi
```

This will allow OrthoMCL to communicate with the MySQL database you just created.

Step 4

(Runs as described in the user guide.)

Step 5

Use an appropriate `cd` command to make sure you are in your `my_orthomcl_dir` directory. Use `mkdir` to make a `compliantFasta` directory within this, and use `cd` to change to this new directory. Copy `bovine_hv_5.fa` and `human_hv_1.fa` from `~/4273pi/week2/` to the current directory.

Choose your own different three- or four-letter abbreviations to represent 'human herpesvirus 1' and 'bovine herpesvirus 5'. For our files, the ID field is 4 (i.e. the fourth part of the Fasta ID, which for proteins from the RefSeq database, is an accession number typically beginning with `NP_` or `YP_`).

Step 6

After running `orthomclFilterFasta`, check that the file `poorProteins.fasta` is empty. This is the list of proteins which were too short or contained too many stop codons for further processing. There should be none.

Step 7

Along the lines of last week's practical, use `formatdb` to create a BLAST database for the sequences in `goodProteins.fa`.

Look at the OrthoMCL Algorithm Document, online, at the URL given in the OrthoMCL User Guide. Run 'all-versus-all BLAST' as described in 'Phase 2 – BLAST' in the Algorithm Document, but omitting the `-z protein_database_size` option. Use protein-protein BLAST (`-p blastp` option of `blastall`), with `goodProteins.fa` as input (`-i` option of `blastall`), `goodProteins.fa` as database (`-d` option of `blastall`), tabular output (`-m8` option of `blastall`); and redirect the output to a file (using > as you did last week).

Step 8

Firstly, run `orthomclBlastParser` without any command-line options, to see instructions for use. When running it 'for real', send output to a file, `similarSequences.txt`, using > (not >> as in the OrthoMCL User Guide; that appends text, rather than deleting any existing file and creating a new one; appending can be confusing if you have to re-run the analysis).

Steps 9, 10, 11, 12

(Run as described in the manual.)

Step 13

Instead of 1000 on the `orthomclMclToGroups` command-line, you can use 1. This will cause your orthologous groups to be given ID tags starting with number 1. The 'prefix' doesn't matter but has to be present. You might choose `g` for 'group', or alternatively spell it out in full, `group`.

Finally

Use `nedit` to take a look at the `groups.txt` file. What does it reveal? Use `bl2seq` to look at pairwise alignments between some members of the same orthologous group. (First run `bl2seq` without further command-line arguments, to see brief instructions on how to use it.) Does it 'seem reasonable' that the sequences are related?

References

Chen F, Mackey AJ, Vermunt JK, Roos DS (2007) Assessing performance of orthology detection strategies applied to eukaryotic genomes. *PLoS ONE* 2: e383.

Fitch, W.M. (1970) Distinguishing homologous from analogous proteins. *Systematic Zoology* 19: 99-113.

Fitch, W.M. (2000) Homology a personal view on some of the problems. *Trends in Genetics* 16: 227-231.

Li L., Stoeckert C.J. and Roos, D.S. (2003) OrthoMCL: identification of ortholog groups for eukaryotic genomes. *Genome Research* 13: 2178-2189.
