# Supplementary material for: 4273π: Bioinformatics education on low cost ARM hardware
Source: BMC Bioinformatics. 2013 Aug 12;14:243. doi: 10.1186/1471-2105-14-243 (PMC3751261; doi:10.1186/1471-2105-14-243)
Supplement: Additional file 2 — 4273π Bioinformatics for Biologists teaching material, Version 1.01. The module handbook, lectures and practicals are included. The latest version, including Linux, software and BLAST databases, is available at the 4273π Web site [25]. [file 1471-2105-14-243-S2.zip › 4273pi_course_material/week1/practical_linux_perl.pdf]

4273π Bioinformatics for Biologists

Practical, Week 1: Linux and Perl

Daniel Barker, School of Biology, University of St Andrews
Email db60@st-andrews.ac.uk

© 2013 D. Barker. This is an Open Access document distributed under the terms of the Creative Commons Attribution License (http://creativecommons.org/licenses/by/2.0), which permits unrestricted use, distribution, and reproduction in any medium, provided the original work is properly cited.

4273π, Version 1.01. http://eggg.st-andrews.ac.uk/4273pi

The learning objective of this practical is to introduce you to the following:

- Raspberry Pi hardware
- The Linux operating system
- Creating, editing and viewing text files in Linux
- Running BLAST at the command-line
- Perl programming
- Running BLAST from within a Perl script

At the end of the practical, you should be ready to use textbooks and Web sites to discover and use further information on each of these subjects.

Work through the material. Probably this will take longer than the time available in the practical class. Continue to experiment with Linux, Perl and BLAST in your own time, before the next practical.

What is the Raspberry Pi?

The Raspberry Pi is a small computer board, developed by the Raspberry Pi Foundation. It has an ARM processor running at about 0.7 GHz, graphics hardware, and either 256 MB or 512 MB of RAM. It uses an SD card instead of a hard disk, generates so little heat that a fan is not required, and is powered by a mobile phone charger. Aside from these points, the Pi can be regarded as broadly equivalent to a desktop computer. On this module, we will use 4273π, a customised version of Raspbian Linux. Raspbian is an operating system recommended by the Raspberry Pi foundation. Raspbian is the Raspberry Pi version of Debian Linux, which is very widely used, in bioinformatics and other areas.

Raspbian, and other versions of Linux, may be regarded as a free version of the UNIX operating system. We have customised our version of Raspbian considerably, to include software, data and documents useful on this module, and refer to this customised version as 4273π. This is named after the undergraduate module at the University of St Andrews, BL4273, in which it was first used, in September–December 2012.

The Raspberry Pi is rather fussy about power and voltage. We have experimented with various peripherals and have had success with a specific set: charger and cable, SD card, keyboard, mouse, USB hub, case. Without any endorsement, these are listed on the 4273π Web site, http://biology.st-andrews.ac.uk/cegg/4273pi.aspx.

Starting the Raspberry Pi

Begin with the power supply and USB hub **disconnected** from the mains.

Carefully insert the SD card, power supply and Ethernet cable into the appropriate sockets on the Pi. Note that the case may not be very strong. Greater care is required than with most computers.

Connect the USB hub to one of the Pi's USB sockets. Connect the mouse to the Pi's other USB socket. Plug the keyboard into the USB hub.

Connect the monitor to the Pi's HDMI socket and switch the monitor on.

Now, connect the USB hub and power supply to the mains.

Starting up

You should see a start-up screen, containing various announcements about the progress of start-up. After a minute or so, you will be prompted for your username and password.

Your username is: **pi**

Your password is: **4273pi**

Once you have successfully logged in, enter the following command to start the graphics system, X11:

```
startx
```

You will now see a reasonably familiar 'desktop'. Several programs can be launched, either by double-clicking icons on the left or via the 'start' menu in the bottom left.

To check the Pi is working OK, start the Web browser IceWeasel, via the 'start' menu. You should be able to access most Web sites in the usual way. The main difference you may notice is, some videos will not display correctly (or at all).

Shutting down

On Linux, most 'serious work' is done by entering commands into Terminal software. The terminal on the Pi is LXTerminal. Start LXTerminal now.

To shut down the Pi, you have to behave as a system administrator, or 'super-user'. This is just a mechanism to prevent you accidentally launching programs which may have more serious consequences than usual. To do something as 'super-user', begin your command with the word `sudo`.

To shut down, type this command in LXTerminal, or paste it from this document, then press ENTER:

```
sudo shutdown -h now
```

You will see various messages about the status of the shut-down. Eventually, these cease and a single red LED is the only light on the Pi. At this point, disconnect the mains and USB hub from the mains supply.

Having verified you can shut down the Pi, start it up again, to continue with the practical.

Creating a text file

We will use the `nedit` text editor. Start editing a new file called `test.txt`, by entering this command in LXTerminal:

```
nedit test.txt &
```

`nedit` warns you that you're creating a new file, which is fine. You now see something like a basic word processor. You can type text, save the file and exit, in the usual sort of way.

Once you have created a text file, you can display its contents, using the `cat` command (in LXTerminal):

```
cat test.txt
```

When you have finished, you can delete the file:

```
rm test.txt
```

`rm` deletes a file permanently, without any straightforward way to 'undelete' if you change your mind.

Exercise. Try omitting the ampersand when launching `nedit`. I.e. what happens if, instead of the above, you type:

```
nedit test.txt
```

Running BLAST on the Raspberry Pi

BLAST is a rapid way to search a large number of sequences (database) for similarity to a sequence of interest (the query). Sequences with high similarity to each other are likely to be homologous and to have similar functions and, in the case of proteins, similar three-dimensional structures.

The BLAST program from the NCBI, the high-quality SwissProt protein sequence database and the entire GenPept protein sequence database (known for historical reasons as 'nr') are all installed on your Raspberry Pi. The databases were downloaded from the NCBI FTP site. Because they are files on your Raspberry Pi's own SD card, they will become out of date as time goes by and new sequences are added to the databases at the NCBI. However, at the time of writing, they are current.

Change into this week's directory:

```
cd ~/4273pi/week1
```

The file `test.fa` contains a protein sequence. Take a look at it, using `nedit` or `cat`.

Now, search for similar sequences in the SwissProt database.

```
blastall -p blastp -d swissprot -i test.fa > test_blast.txt
```

This may take a minute or two to finish.

`blastall` is the program for running BLAST. `-p blastp` means 'search a protein database with a protein sequence', also known as the BLASTP program. `-d swissprot` means 'search the database named `swissprot`'. `-i test.fa` means 'use the sequence in `test.fa` as the query'. Finally, `> test_blast.txt` tells Linux to send the output to a file, `test_blast.txt`, instead of to the screen. (Messages about major errors will, however, still be sent to the screen.)

Examine the output in `nedit`. Are you able to propose an identity for your sequence?

Scroll down a few pages. You will see the output from BLAST is extensive. Now run the BLAST search again, but request tabular output from BLAST:

```
blastall -p blastp -d swissprot -i test.fa -m9 > test_m9.txt
```

Repeat this with a slight variation:

```
blastall -p blastp -d swissprot -i test.fa –m8 > test_m8.txt
```

The `–m8` and `–m9` options are documented briefly in the summary of options you get if you enter the command

```
blastall
```

on its own.

Take a look at the output files. Can you relate them to each other and to `test_blast.txt`? What are the relative merits of full BLAST output or tabular BLAST output, for (a) human readers and (b) computational processing of results?

Exercise. In your own time, repeat the above BLAST commands, but searching the `nr` database (i.e. GenPept) instead of `swissprot`. Warning! `nr` is a very large database, so each BLAST search may take some hours to complete on the Raspberry Pi.

Perl Scripts

Perl is a widely used and freely available programming language, popular for bioinformatics.

A First Perl Script

Use `nedit` to create a text file, named `first.pl`, with the following contents. There must be no blank lines or spaces at the start.

```
#!/usr/bin/perl -w

use strict;

print "Hello\n";
```

This is a Perl script. What do you think it does?

Next, make the file executable (i.e. make it possible to use it as a program), as follows:

```
chmod +x first.pl
```

Now you can run the script:

```
./first.pl
```

If it works – congratulations! Next, run the script but send the output to a file rather than the screen:

```
./first.pl > output.txt
```

Take a look at the output:

```
cat output.txt
```

Now delete it:

```
rm output.txt
```

To return to your first Perl script. The first line of the file is:

```
#!/usr/bin/perl -w
```

This tells Linux to run this program with Perl, using the `-w` option. The `-w` option causes Perl to output warnings when it spots likely mistakes in your program. Every Perl script you write should begin with this line.

The next part, '`use strict;`', will entirely prevent the program working if certain error-prone aspects of the Perl language are used. This is a good idea. The semi-colon ends the Perl statement.

It is recommended to always begin a Perl program with the `-w` option and `use strict`. They can save a lot of time later, by preventing certain problems entirely and warning you about others. Note, `use strict` may prevent example programs from some textbooks from working. If so, Perl will report the problem when you run the script. It may be a useful exercise to 'correct' the program!

Finally, in the print statement you might wonder what `\n` means. This represents the 'newline' (end-of-line) character. Without this, the output of the script will be a bit of a mess.

Variables and Conditions

We rarely want a program to perform a single identical action under all conditions. Try this more complicated Perl script:

```
#!/usr/bin/perl -w

use strict;

my $i = 1;
while ($i <= 12) {
    my $j = 11 * $i;
    print "$i elevens are $j\n";
    $i = $i + 1;
}
```

Here we create a variable, `$i`. Perl **scalar** variables (holding a single value, e.g. a number or a piece of text) always have names beginning with `$`. We then use a ‘`while` loop’ to print out the eleven times table. The ‘condition’ for the `while` loop is enclosed by round brackets. The ‘conditional’ part is enclosed by curly brackets. If the condition is true, the conditional part is performed. At the end of the conditional part, Perl checks the condition again. If it is still true, the conditional code is performed again. If the condition is false, the script continues after the closing curly bracket. Since we have nothing there, the script ends.

A note on the statement `$i = $i + 1`. This doesn’t make much sense mathematically! However, this shorthand is typical of programming languages. Really it means ‘calculate `$i + 1`; replace `$i` with this value’. In other words, in Perl, the equals sign is used for assigning values to variables (like `<-` in the R software environment). For the usual, mathematical equals sign, Perl uses two equals signs right next to each other, ==, as we will see in the next example. It is crucially important not to mix up = and ==. Often the result will be a valid Perl program, but not one that does what you want! This confusion is a major source of errors.

The Perl in the last example may be OK, but you will have noticed a grammatical error in the English of the output! This could be corrected by using Perl’s `if` and `else`:

```
#!/usr/bin/perl -w

use strict;

my $i = 1;
while ($i <= 12) {
    my $j = 11 * $i;
    if ($i == 1) {
        print "$i eleven is $j\n";
    } else {
        print "$i elevens are $j\n";
    }
    $i = $i + 1;
}
```

<u>Exercise</u>. Replace the number ‘11’ in the above script with another variable, set to 11 (or another value) near the top of the script. Give this variable a meaningful name.

Variables can have longer names than `$i` or `$j`, so long as the first part of the name, after the `$`, is a letter. Unlike in R, Perl variables cannot have a full stop within their name. An underscore is allowed. Put a comment near the definition of this variable, explaining its purpose. Comments begin with a hash, `#`. When there is a hash, from that point to the end of the line will be ignored. This provides a useful way to document your Perl scripts so that you, or others, can understand them later.

Arrays

It is often useful to store a list of values together. The computer terminology for a list is an **array**. In Perl, names of arrays begin with an `@` sign. The following script outputs various times tables, one after the other:

```
#!/usr/bin/perl -w

use strict;

my @tables = ( 9, 10, 11 );    # array of 3 scalars

foreach my $table (@tables) {
    my $i = 1;
    while ($i <= 12) {
        my $j = $table * $i;
        print "$i x $table = $j\n";
        $i = $i + 1;
    }
    print "\n";
}
```

The `foreach` statement sets a scalar, `$table`, to equal each element of the array `@tables` in turn. The instructions surrounded by curly brackets are performed on each.

Exercise. Use the Web, or a textbook, to find out about Perl `for` loops and `until` loops. Change the example above to use (a) a `for` loop, and then (b) an `until` loop, instead of `foreach`.

File Input

The above Perl scripts do not use any input – everything they require is in the script itself. In practice though, it is often useful to read data from a file. For example, we could specify the times tables we require in a text file, rather than in the Perl script itself. This would make the script more flexible and more accessible to non-specialist users.

Create a text file, `tables.txt`, with the following contents:

```
7 8 9 10 3
11 12
```

Modify your Perl script to be as follows:

```
#!/usr/bin/perl -w

use strict;

my @tables = ();
open(TABLES_IN_FH, "tables.txt") or die "error";
while (<TABLES_IN_FH>) { # while there is an unread line
    chomp;               # remove end-of-line character
    my @elements = split;     # split on any spaces/tabs
    push @tables, @elements;  # add numbers to the list
}

foreach my $table (@tables) {
    my $i = 1;
    while ($i <= 12) {
        my $j = $table * $i;
        print "$i x $table = $j\n";
        $i = $i + 1;
    }
    print "\n";
}
```

Now, run the script. If all goes well it will read the numbers from `tables.txt` and act accordingly.

Then, delete the `tables.txt` file, run the script again, and see what happens. Is there a useful error message? Can you improve it?

Bringing it All Together: Running BLAST from a Perl Script

Instead of typing a command, you may launch it from Perl using the `system` function:

```
#!/usr/bin/perl -w

use strict;

die if system("blastall -p blastp -d swissprot "
    . "-i test.fa -m8 > script_out.txt");

open(BLAST_RES_FH, "script_out.txt") or die;

while (<BLAST_RES_FH>) {
    my @elements = split;
    my $hit_id = $elements[1];
    my $e_value = $elements[10];
    if ($hit_id =~ /\|Q55629\.1\|/) {
        my $output_line = join("\t", @elements);
        print "found match to Q55629.1\n";
        print "$output_line\n\n";
    }
}
```

Perl's `system` function is extremely useful for automating repetitive tasks. We can arrange for a script to launch a large number of analyeses, which overall would be impossible (or at least, tedious and error-prone) to run individually at the command prompt.

Further Reading

See books on UNIX and Perl in the reference list of the 'Bioinformatics, Sequences and Genomes' lecture this week. Perl is covered briefly in a bioinformatics context by Mount (2004, Chapter 12) and by Krane and Raymer (2003, Appendix 1). For further details of BLAST see Korf et al. (2003). The latter covers two versions of BLAST. The one installed with 4273π is NCBI BLAST.

A brief guide to some of the most useful Linux commands is given in an Appendix at the end of this hand-out. Please read through this now.

References

Korf, I., Yandell, M. and Bedell, J. (2003) *BLAST* (Sebastopol, California: O'Reilly).

Krane, D.E. and Raymer, M.L. (2003) *Fundamental Concepts of Bioinformatics* (San Francisco: Benjamin Cummings).

Mount, D.W. (2004) Bioinformatics: Sequence and Genome Analysis (Cold Spring Harbour: Cold Spring Harbour Laboratory Press).

Appendix: Some useful Linux commands

CTRL-D	This transmits an end-of-file signal, and if typed at the command prompt will close the terminal window. However, it will not shut down the Raspberry Pi.
`cat` *filename*	Display contents of file named *filename*.
`cd` *dirname*	Change into directory (folder) named *dirname*. All commands will then regard this directory as the default location to work in.
`chmod` *filename*	Change the access permissions for file *filename*. You are unlikely to want to do this apart from `chmod +x` *filename*, which gives the file named *filename* execute permission so that it can run as a program.
`cp` *file1 file2*	Copy file *file1* to *file2*. If *file2* already exists, its previous contents will be destroyed. If *file2* is a directory (folder), a copy of *file1* is placed within that directory.
`head` *filename*	Display the start of the file named *filename*.
`head -20` *filename*	Display the first 20 lines of file named *filename*. You can use other numbers instead of 20.
`ls`	List files in the current directory (folder).
`ls -Fl`	List files in the current directory (folder), indicating which files are directories (name is shown followed by a slash) and which are executable (name is shown followed by a star), and giving information on files including their size.
`ls -Fl` *dirname*	As above but list contents of directory (folder) named *dirname*.
`man` *command*	Display the Linux manual for *command*, if there is one. Navigate the manual as with the `more` command, below.
`mkdir` *dirname*	Make a directory (folder) named *dirname.*
`more` *filename*	Display contents of file named *filename*, one part at a time. Useful for files that cannot fit easily in your terminal window. Press SPACE to see the next part, MINUS to see the previous part, or `q` to quit.
`mv` *file1 file2*	Move *file1* to directory and/or file name *file2*. The `mv` command can be used to re-name or to move a file. If *file2* gives the name of a file that already exists, its previous contents will be destroyed. If *file2* gives the name of a directory (folder) that exists, *file1* will be moved into that directory.

`nedit` *filename* — Open file named filename in the `nedit` text editor. If filename does not exist already, saving the file in `nedit` will create it.

`nedit -read` *filename*

Open file named *filename* in the `nedit` text editor, in such a way that it is more difficult to save changes to the file. Use this to look at important files you wish to preserve unchanged.

`passwd` — Change your password. You will be asked for your current password, and then for the new password, twice. The default password on your Raspberry Pi is rather short. This is because we assume you are the only user of the SD card; and the 4273π SD card image includes a software firewall, reducing the opportunity for hostile incoming traffic on the network. But in general, passwords ought to be at least eight characters long and should include at least one character that isn't a letter (e.g. perhaps a number or an underscore). Avoid a password which is a word or similar to a word. It is more secure to think of a sentence, then take the first (or second, etc.) letter from each word in the sentence as the basis of your password. Be warned, if you set your password to something you subsequently forget, you will be denied access to your Raspberry Pi. (You can re-create a functioning SD card from the 4273π SD card image, but any of your files will be lost.)

`rm` *filename* — Delete the file named *filename.* Be warned, there is no 'trash can' – this command is effectively irreversible, unless one has the resources of the CIA to unpick the contents of the SD card.

`rm -r` *dirname* — Remove directory (folder) named *dirname*, and all of its contents. Be warned, wirh `rm` there is no 'trash can' – this command is effectively irreversible. Careless use of this command can lead to destruction of a lot of your hard work!

`tail` *filename* — Display the end of the file named *filename*.

`tail -20` *filename* — Display the last 20 lines of file named *filename*. You can use other numbers instead of 20.

`unzip` *dir.zip* — Un-zips the zipfile named *dir.zip* (see `zip`, below). Since this can lead to existing files being over-written, it is safest to do this in an empty directory, e.g. this series of commands:

```
mkdir tmpdir
cd tmpdir
unzip ../work.zip
```

The zip file, *dir.zip*, is left as it was.

`zip -r` *dir dir* — Creates a zipfile from the directory (folder) named *dir*. The zipfile will have the same name as the directory, but with `.zip` added to the end. The directory is left as it was.
