# Supplementary material for: 4273π: Bioinformatics education on low cost ARM hardware
Source: BMC Bioinformatics. 2013 Aug 12;14:243. doi: 10.1186/1471-2105-14-243 (PMC3751261; doi:10.1186/1471-2105-14-243)
Supplement: Additional file 2 — 4273π Bioinformatics for Biologists teaching material, Version 1.01. The module handbook, lectures and practicals are included. The latest version, including Linux, software and BLAST databases, is available at the 4273π Web site [25]. [file 1471-2105-14-243-S2.zip › 4273pi_course_material/week1/lecture_bioinformatics_seqs_genomes.pdf]

Bioinformatics, Sequences and Genomes

4273π Bioinformatics for Biologists

Lecture, Week 1

Daniel Barker, School of Biology, University of St Andrews

Email db60@st-andrews.ac.uk

© 2013 D. Barker. This is an Open Access document distributed under the terms of the Creative Commons Attribution License (http://creativecommons.org/licenses/by/2.0), which permits unrestricted use, distribution, and reproduction in any medium, provided the original work is properly cited.

4273π, Version 1.01. http://eggg.st-andrews.ac.uk/4273pi

What is bioinformatics?

- Use of computers in biology
- DNA, RNA, proteins
  - sequences, structure, function, expression
- Amount of data is large
  - Release 112 of the EMBL-Bank nucleotide database (June 2012) contains **429,512,389,024** nucleotides
- Degree of automation should be as high as possible

Nucleotide database growth

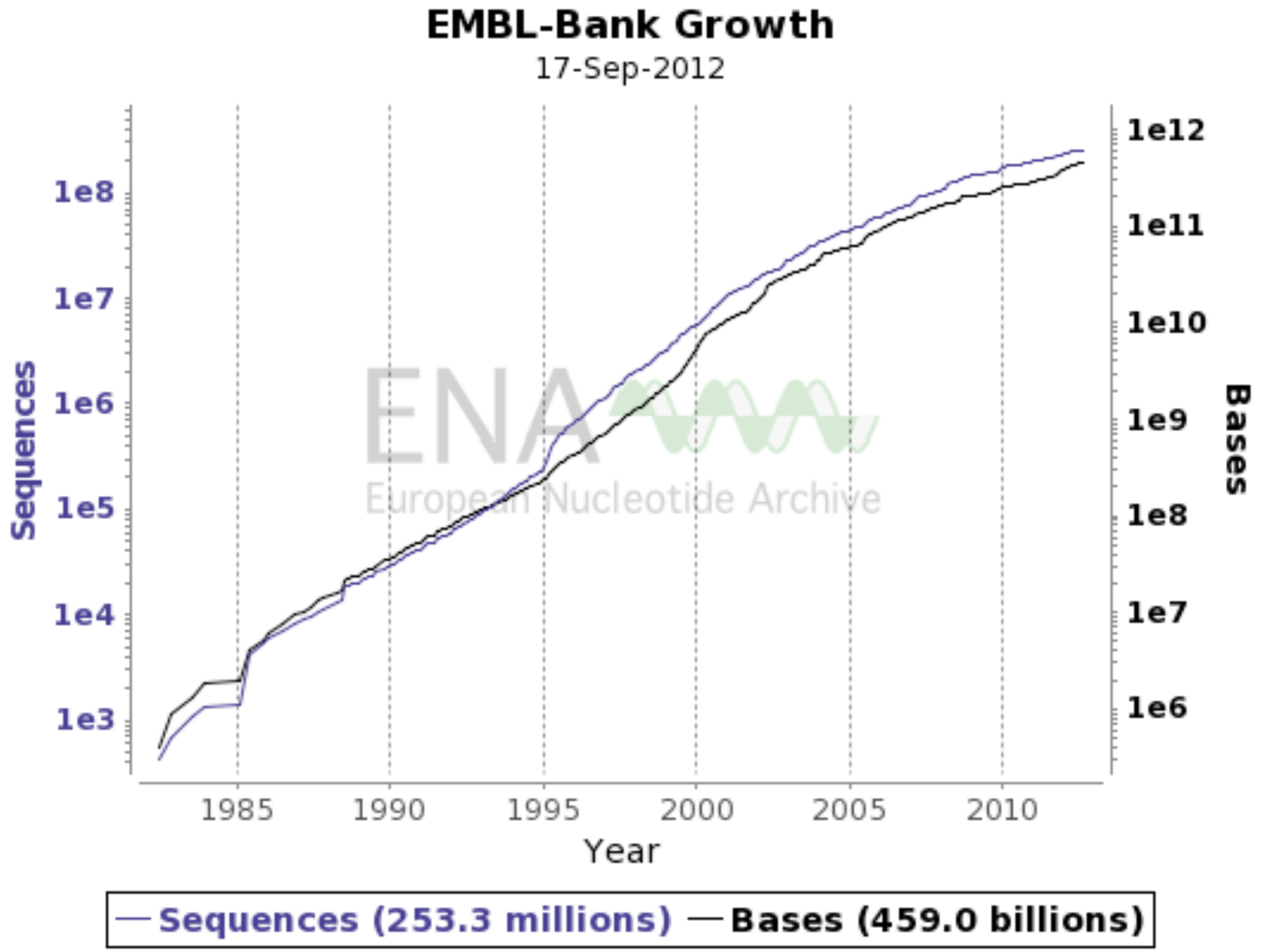

Source: http://www.ebi.ac.uk/embl/Services/DBStats

The major nucleotide sequence databases

- GenBank (USA), EMBL (EU), DDBJ (Japan)
- These three databases share their data.

- To access the data, we can use **Entrez** in the USA
  - http://www.ncbi.nlm.nih.gov/Entrez

A typical DNA record in Entrez

accession.version

```
LOCUS       X13028                  2785 bp    DNA     linear   PLN 13-MAY-1994
DEFINITION  Yeast CTA1 gene for catalase A.
ACCESSION   X13028 M36510
VERSION     X13028.1  GI:3604
KEYWORDS    catalase; catalase A; CTA1 gene.
SOURCE      Saccharomyces cerevisiae (baker's yeast)
  ORGANISM  Saccharomyces cerevisiae
            Eukaryota; Fungi; Ascomycota; Saccharomycotina; Saccharomycetes;
            Saccharomycetales; Saccharomycetaceae; Saccharomyces.
REFERENCE   1  (bases 1 to 2785)
  AUTHORS   Cohen,G., Rapatz,W. and Ruis,H.
  TITLE     Sequence of the Saccharomyces cerevisiae CTA1 gene and amino acid
            sequence of catalase A derived from it
  JOURNAL   Eur. J. Biochem. 176 (1), 159-163 (1988)
   PUBMED   3046940
COMMENT     On Jan 25, 2006 this sequence version replaced gi:171325.
FEATURES             Location/Qualifiers
     source          1..2785
                     /organism="Saccharomyces cerevisiae"
                     /mol_type="genomic DNA"
                     /db_xref="taxon:4932"
```

Source: NCBI Entez, http://www.ncbi.nlm.nih.gov/gquery

```
                     /clone="pUC19"
     CDS             820..2367
                     /note="unnamed protein product; catalase A (AA 1-515)"
                     /codon_start=1
                     /protein_id="CAA31443.1"
                     /db_xref="GI:3605"
                     /db_xref="GOA:P15202"
                     /db_xref="InterPro:IPR002226"
                     /db_xref="InterPro:IPR011614"
                     /db_xref="PDB:1A4E"
                     /db_xref="SGD:S000002664"
                     /db_xref="UniProtKB/Swiss-Prot:P15202"
                     /translation="MSKLGQEKNEVNYSDVREDRVVTNSTGNPINEPFVTQRIGEHGP
                     LLLQDYNLIDSLAHFNRENIPQRNPHAHGSGAFGYFEVTDDITDICGSAMFSKIGKRT
                     KCLTRFSTVGGDKGSADTVRDPRGFATKFYTEEGNLDWVYNNTPVFFIRDPSKFPHFI
                     HTQKRNPQTNLRDADMFWDFLTTPENQVAIHQVMILFSDRGTPANYRSMHGYSGHTYK
                     WSNKNGDWHYVQVHIKTDQGIKNLTIEEATKIAGSNPDYCQQDLFEAIQNGNYPSWTV
                     YIQTMTERDAKKLPFSVFDLTKVWPQGQFPLRRVGKIVLNENPLNFFAQVEQAAFAPS
                     TTVPYQEASADPVLQARLFSYADAHRYRLGPNFHQIPVNCPYASKFFNPAIRDGPMNV
                     NGNFGSEPTYLANDKSYTYIQQDRPIQQHQEVWNGPAIPYHWATSPGDVDFVQARNLY
                     RVLGKQPGQQKNLAYNIGIHVEGACPQIQQRVYDMFARVDKGLSEAIKKVAEAKHASE
                     LSSNSKF"
ORIGIN
        1 gaattcttag aaggtgaaga aatagtacta gattcatatg attgttataa taatggtgaa
       61 ttgttgcctc aactaatact tttggtccaa atcttgacaa ttctttgcca aattccaggt
```

```
 121 ttatgcaaac tggacataaa agcaatggaa aggcaagtgg agagaattgt aaagaagtgt
 181 ttacaattga tagaaggtgc ccgcgccact acaaactgta gtgccacatg gaaacgttgt
 241 attatgaagc gtctagccga ttaccccata aaaaagtgcg tttctatcga aaaaccttcg
 301 aaaggaaact cattaacaag ggaagaacta agagatgtta tggctcggag agttttgaaa
 361 agcgaaatag attcgctgca agtttgtgaa gaaaccatcg acaagaatta caaggttatt
 421 cctgatgaaa agctgctaac taatatttta aagagaaagt tgacagagga agaaaaaagc
 481 tctgtcaaac gtccttgcgt gaagaagtga gcggttgttc taaccactat ttaaagccgc
 541 aattagtaat gcaaaaagtt ggccggaatt agccgcgcaa gttggtgggg tcccttaatc
 601 cgaaaaagga cggctttaac aaatataaac tccgaaaatc cccacagtga cagaattgga
 661 gaaacaacca gttttgatat cgccatacat ataaagagat gtagaaagca ttcttcactg
 721 taatgtccaa atcgtacatt tgaatttctt gtaggtttat ttaaaaggta agttaaataa
 781 atataatagt acttacaaat aaatttggaa ccctagaaga tgtcgaaatt gggacaagaa
 841 aaaaatgaag taaattactc tgatgtaaga gaggatagag ttgtgacaaa ctccactggt
 901 aatccaatca atgaaccatt tgtcacccaa cgtattgggg aacatggccc tttgcttttg
 961 caagattata acttaattga ttctttggct catttcaaca gggaaaatat tcctcaaagg
1021 aatccacatg ctcatggttc tggtgccttc ggctattttg aagtaaccga tgacattact
1081 gatatctgcg ggtctgctat gtttagtaaa attgggaaaa gaacgaaatg tctaacaaga
1141 ttttcgactg tgggtggtga taaaggtagt gccgacacgg ttcgtgatcc aagggggttt
1201 gccaccaaat tctacactga agaaggtaat ttagattggg tctacaataa tacaccggta
1261 ttctttatca gagacccttc caagttccct cactttatcc acacacagaa gagaaaccca
1321 caaaccaacc taagggatgc tgacatgttt tgggatttcc tcaccactcc tgaaaatcag
1381 gtggccattc atcaagtaat gatccttttt tcagaccgtg gtacccctgc caactaccgt
1441 agtatgcatg gttattctgg tcatacctat aaatggtcca ataaaaacgg agattggcat
1501 tatgtgcaag ttcatatcaa aaccgatcaa ggaataaaga atttgaccat agaagaggct
1561 accaaaattg cgggatccaa tccagattac tgccagcagg atttatttga ggctattcag
```

```
1621 aatggaaact atccttcctg gacagtttat attcaaacaa tgaccgaacg cgatgccaaa
1681 aaattaccat tttcagtctt tgatttgact aaagtatggc ctcaggggca attcccttta
1741 cggcgtgtgg gtaagattgt tttgaacgag aatccactga acttcttcgc acaggtggaa
1801 caagctgcct tcgcccccag taccacggtt ccttaccaag aagcaagcgc tgatccagta
1861 ttacaggccc gtttgttttc atatgcggat gctcatagat acaggctagg tcctaacttc
1921 catcaaatac ccgtaaactg tccatatgca tctaaatttt tcaatcccgc tatcagagat
1981 ggaccgatga atgttaacgg caacttcggc tcagaaccta catatttggc caacgataaa
2041 tcgtacacgt atatccaaca ggacagaccc attcaacaac accaagaggt atggaatggg
2101 ccagctatcc cttatcattg ggcaacatcc ccaggtgatg tagatttcgt gcaagcaaga
2161 aatctctacc gcgttttggg taaacaacct ggacagcaaa agaacttggc atataacatc
2221 ggcattcatg tagaaggcgc ctgtcctcaa atacagcagc gcgtttatga tatgtttgct
2281 cgtgttgata agggactatc tgaggcaatt aaaaaagtag ctgaggcaaa acatgcttct
2341 gagctttcga gtaactccaa attttgaaac gctcaagtaa caaatgagtg gcgttgtttc
2401 cacgacaatt atttatgata gtgtgtattt ttaacacatt ttatttatta caatttatgt
2461 attttgttat gaattattta tttatacgac taataggtga tgctcatatt ctcgtgttag
2521 aaagttaaaa aaattatcat ttcacacata ggaaagctcg tcgcgccggg gaaaaagctg
2581 aggaatctct attattaggg gtaaagttca acacattcag tatgagataa gtgtgtcttc
2641 aagagagatg cagcactgag tagggaacca agaaacgatg tctgaattac tagatagctt
2701 tgagacagag tttgcgaaat tttataccga cagcaatctg gaagagacaa accttcaaaa
2761 atgtcttgat catactcatg aattc
//
```

Same sequence in **Fasta format**

```
>gi|3604|emb|X13028.1| Yeast CTA1 gene for catalase A
GAATTCTTAGAAGGTGAAGAAATAGTACTAGATTCATATGATTGTTATAATAATGGTGAATTGTTGCCTC
AACTAATACTTTTGGTCCAAATCTTGACAATTCTTTGCCAAATTCCAGGTTTATGCAAACTGGACATAAA
AGCAATGGAAAGGCAAGTGGAGAGAATTGTAAAGAAGTGTTTACAATTGATAGAAGGTGCCCGCGCCACT
ACAAACTGTAGTGCCACATGGAAACGTTGTATTATGAAGCGTCTAGCCGATTACCCCATAAAAAAGTGCG
TTTCTATCGAAAAACCTTCGAAAGGAAACTCATTAACAAGGGAAGAACTAAGAGATGTTATGGCTCGGAG
AGTTTTGAAAAGCGAAATAGATTCGCTGCAAGTTTGTGAAGAAACCATCGACAAGAATTACAAGGTTATT
CCTGATGAAAAGCTGCTAACTAATATTTTAAAGAGAAAGTTGACAGAGGAAGAAAAAAGCTCTGTCAAAC
GTCCTTGCGTGAAGAAGTGAGCGGTTGTTCTAACCACTATTTAAAGCCGCAATTAGTAATGCAAAAAGTT
GGCCGGAATTAGCCGCGCAAGTTGGTGGGGTCCCTTAATCCGAAAAAGGACGGCTTTAACAAATATAAAC
TCCGAAAATCCCCACAGTGACAGAATTGGAGAAACAACCAGTTTTGATATCGCCATACATATAAAGAGAT
GTAGAAAGCATTCTTCACTGTAATGTCCAAATCGTACATTTGAATTTCTTGTAGGTTTATTTAAAAGGTA
AGTTAAATAAATATAATAGTACTTACAAATAAATTTGGAACCCTAGAAGATGTCGAAATTGGGACAAGAA
AAAAATGAAGTAAATTACTCTGATGTAAGAGAGGATAGAGTTGTGACAAACTCCACTGGTAATCCAATCA
ATGAACCATTTGTCACCCAACGTATTGGGGAACATGGCCCTTTGCTTTTGCAAGATTATAACTTAATTGA
TTCTTTGGCTCATTTCAACAGGGAAAATATTCCTCAAAGGAATCCACATGCTCATGGTTCTGGTGCCTTC
GGCTATTTTGAAGTAACCGATGACATTACTGATATCTGCGGGTCTGCTATGTTTAGTAAAATTGGGAAAA
GAACGAAATGTCTAACAAGATTTTCGACTGTGGGTGGTGATAAAGGTAGTGCCGACACGGTTCGTGATCC
AAGGGGGTTTGCCACCAAATTCTACACTGAAGAAGGTAATTTAGATTGGGTCTACAATAATACACCGGTA
TTCTTTATCAGAGACCCTTCCAAGTTCCCTCACTTTATCCACACACAGAAGAGAAACCCACAAACCAACC
TAAGGGATGCTGACATGTTTTGGGATTTCCTCACCACTCCTGAAAATCAGGTGGCCATTCATCAAGTAAT
GATCCTTTTTTCAGACCGTGGTACCCCTGCCAACTACCGTAGTATGCATGGTTATTCTGGTCATACCTAT
```

Source: NCBI Entez, http://www.ncbi.nlm.nih.gov/gquery

AAATGGTCCAATAAAAACGGAGATTGGCATTATGTGCAAGTTCATATCAAAACCGATCAAGGAATAAAGA
ATTTGACCATAGAAGAGGCTACCAAAATTGCGGGATCCAATCCAGATTACTGCCAGCAGGATTTATTTGA
GGCTATTCAGAATGGAAACTATCCTTCCTGGACAGTTTATATTCAAACAATGACCGAACGCGATGCCAAA
AAATTACCATTTTCAGTCTTTGATTTGACTAAAGTATGGCCTCAGGGGCAATTCCCTTTACGGCGTGTGG
GTAAGATTGTTTTGAACGAGAATCCACTGAACTTCTTCGCACAGGTGGAACAAGCTGCCTTCGCCCCCAG
TACCACGGTTCCTTACCAAGAAGCAAGCGCTGATCCAGTATTACAGGCCCGTTTGTTTTCATATGCGGAT
GCTCATAGATACAGGCTAGGTCCTAACTTCCATCAAATACCCGTAAACTGTCCATATGCATCTAAATTTT
TCAATCCCGCTATCAGAGATGGACCGATGAATGTTAACGGCAACTTCGGCTCAGAACCTACATATTTGGC
CAACGATAAATCGTACACGTATATCCAACAGGACAGACCCATTCAACAACACCAAGAGGTATGGAATGGG
CCAGCTATCCCTTATCATTGGGCAACATCCCCAGGTGATGTAGATTTCGTGCAAGCAAGAAATCTCTACC
GCGTTTTGGGTAAACAACCTGGACAGCAAAAGAACTTGGCATATAACATCGGCATTCATGTAGAAGGCGC
CTGTCCTCAAATACAGCAGCGCGTTTATGATATGTTTGCTCGTGTTGATAAGGGACTATCTGAGGCAATT
AAAAAAGTAGCTGAGGCAAAACATGCTTCTGAGCTTTCGAGTAACTCCAAATTTTGAAACGCTCAAGTAA
CAAATGAGTGGCGTTGTTTCCACGACAATTATTTATGATAGTGTGTATTTTTAACACATTTTATTTATTA
CAATTTATGTATTTTGTTATGAATTATTTATTTATACGACTAATAGGTGATGCTCATATTCTCGTGTTAG
AAAGTTAAAAAAATTATCATTTCACACATAGGAAAGCTCGTCGCGCCGGGGAAAAAGCTGAGGAATCTCT
ATTATTAGGGGTAAAGTTCAACACATTCAGTATGAGATAAGTGTGTCTTCAAGAGAGATGCAGCACTGAG
TAGGGAACCAAGAAACGATGTCTGAATTACTAGATAGCTTTGAGACAGAGTTTGCGAAATTTTATACCGA
CAGCAATCTGGAAGAGACAAACCTTCAAAAATGTCTTGATCATACTCATGAATTC

What is a genome?

- Sum total of heritable nucleic acids in an individual (or organelle, or virus particle)
  - DNA for some viruses and all (other) life
  - RNA for most viruses

‘Raw’ genome data

- Genome sequence
- Transcriptome sequence
- Gene expression data
- Variation data (polymorphisms, repeats, allele frequencies)
- Subsequent bioinformatic analysis
  - Genome annotation
  - Functional annotation

Growth of genome sequence databases

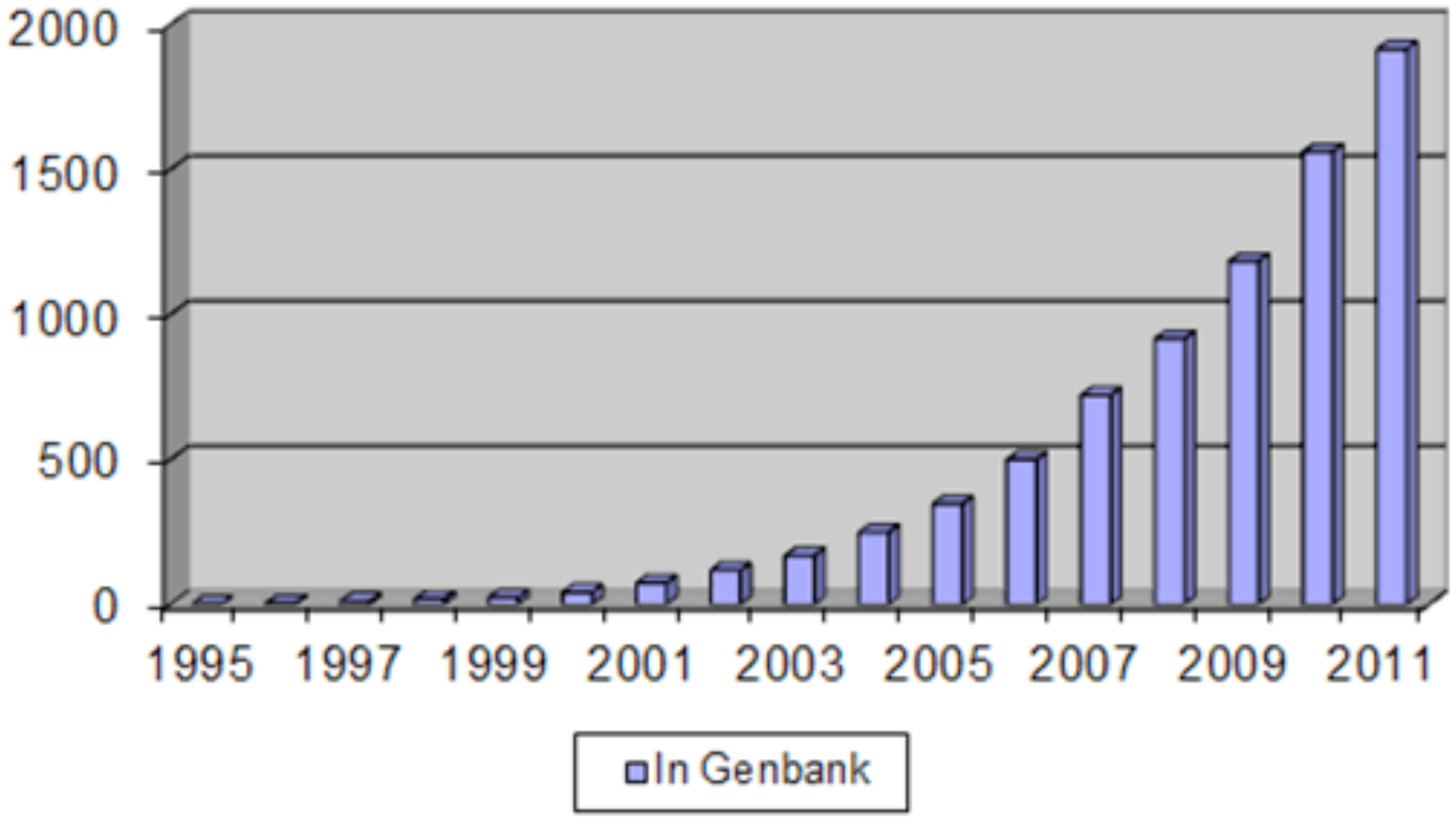

To October 2011. Source: GOLD database, http://www.genomesonline.org

Genome sequencing

- 'Decent job' sequencing a eukaryotic genome: £20,000 to £1,000,000.
- 'Decent job' sequencing a eukaryotic transcriptome: £1,000 to £2,000.
- Technology is moving fast. Sequencing a eukaryotic genome to **draft** standard will soon cost < £1,000.
- (Doing a 'proper job' of sequencing a genome – to '**finished**' standard – remains extremely expensive.)

Genome analysis

Stage:	Methods:
• Genome sequencing	(laboratory)
• **Genome assembly**	**computers**
• **Genome annotation**	**computers**
• **Computational genome analysis**	
• **single-genome analyses**	**computers**
• **comparative analyses**	**computers**
• (Validate computational predictions	laboratory)

DNA sequencing → *Reads*

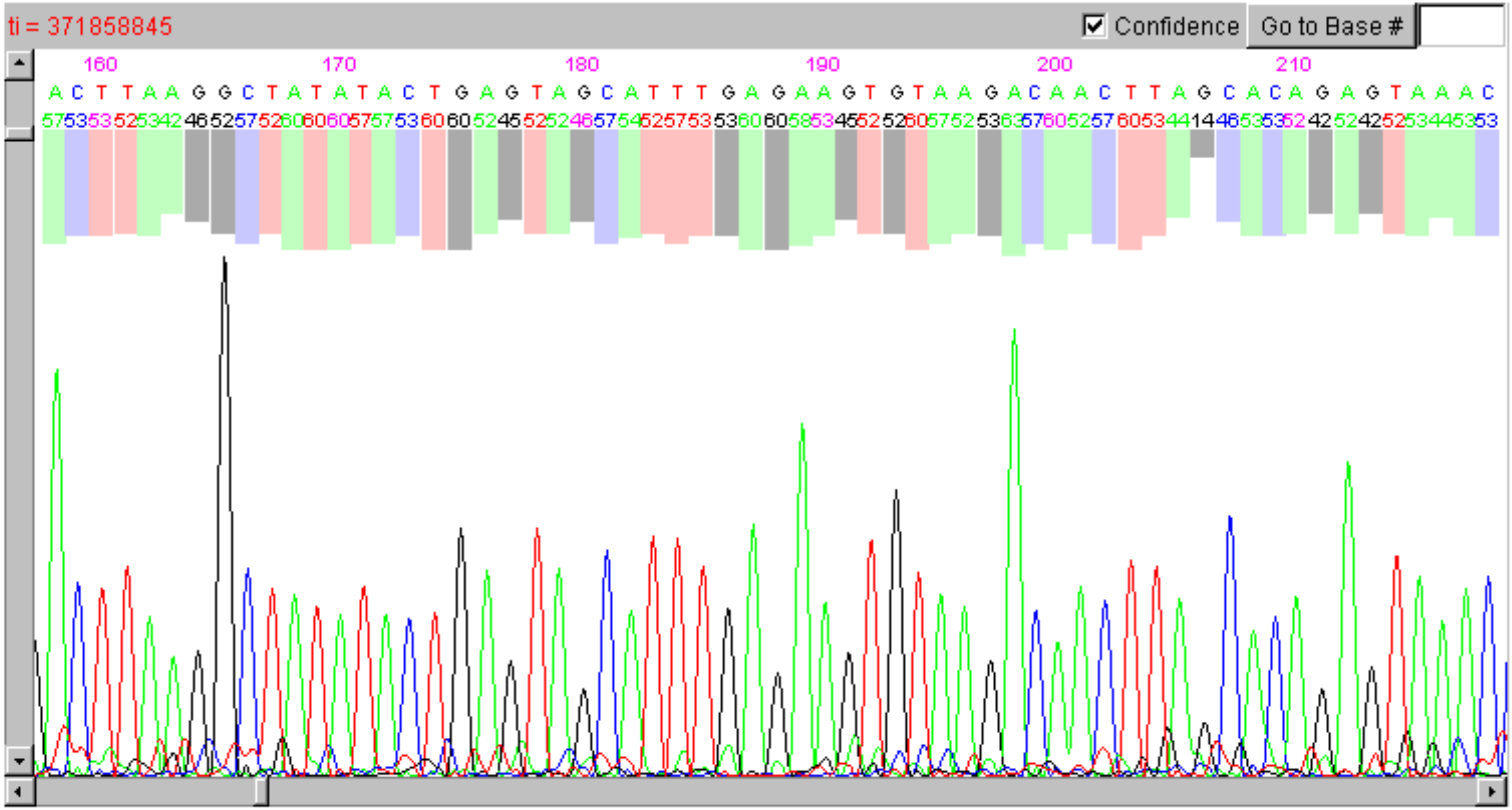

Source: National Center for Biotechnology Information Traces Archive, www.ncbi.nlm.nih.gov/Traces

Genome assembly → *Genomes*

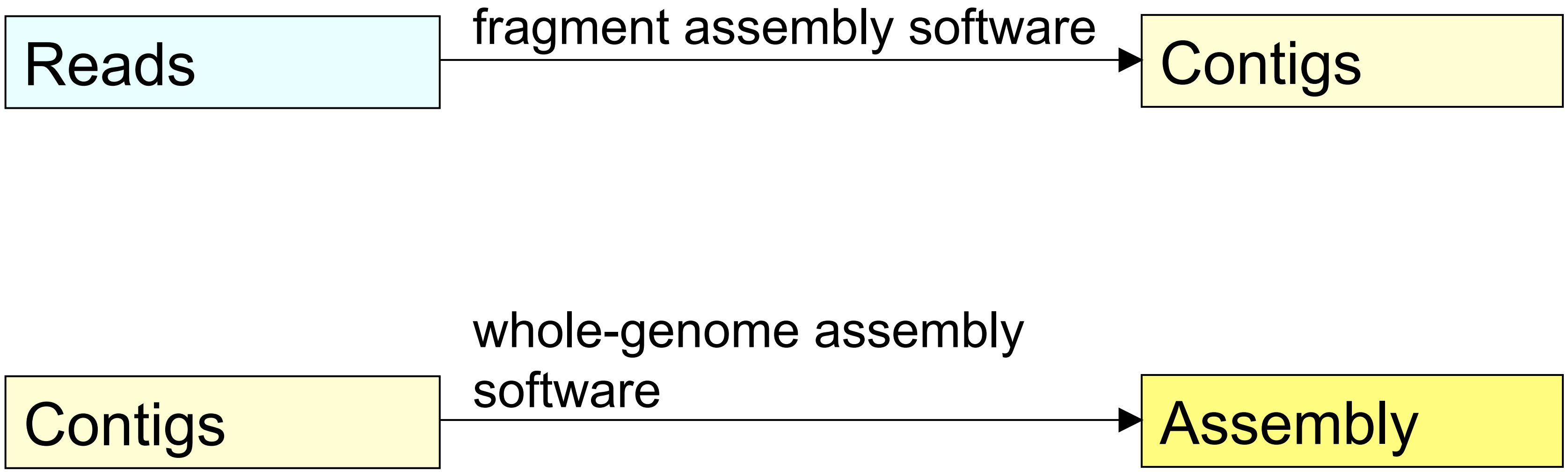

Genome annotation → *Genes*

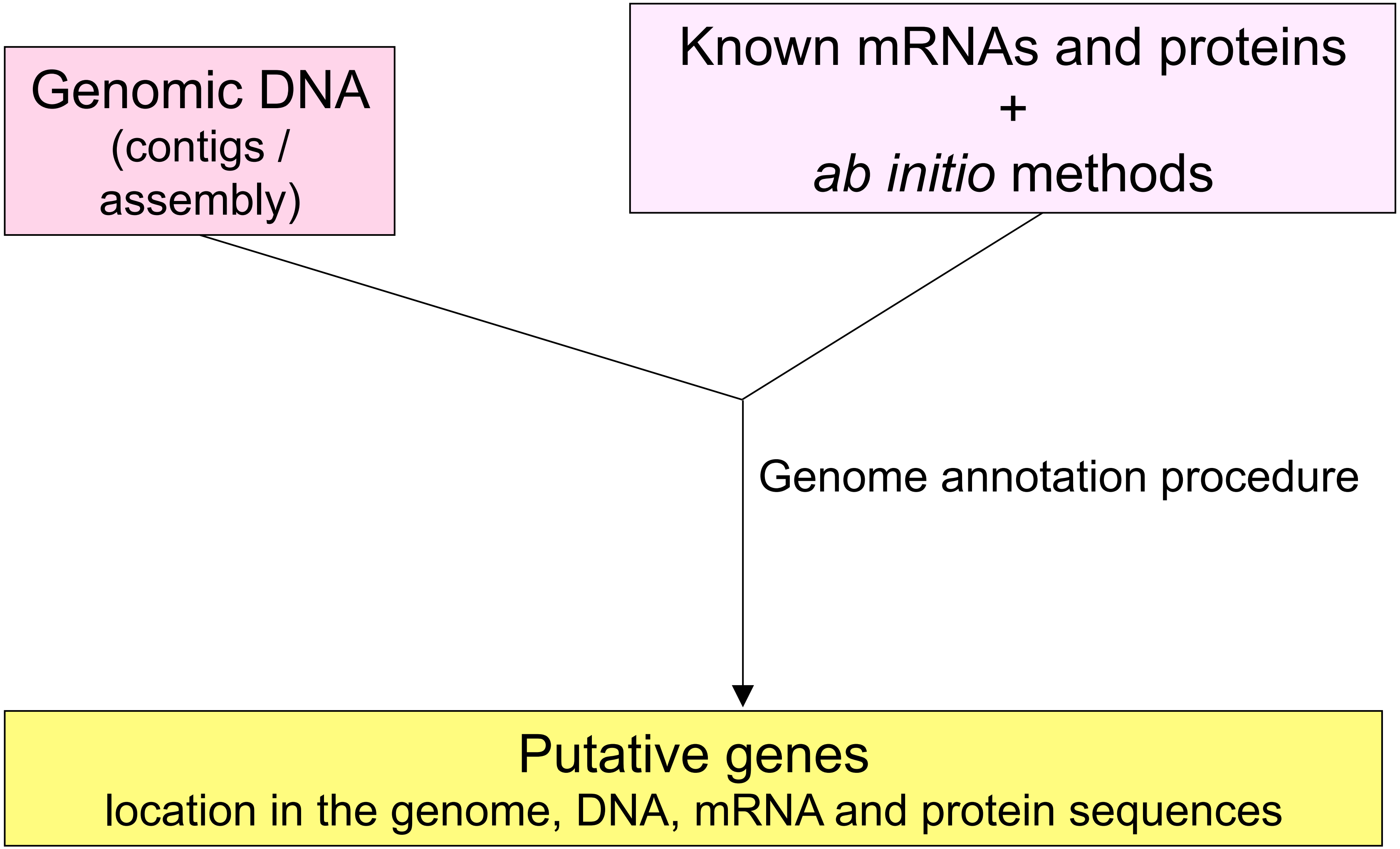

Genes → *Biology*

- How do genes vary?
  - Correlate with environmental, phenotypic and genomic features
- How are gene products affected?
  - Secondary and tertiary structure
  - Active sites
  - Protein-protein and protein-DNA interaction surfaces
- What are the consequences?
  - Disease
  - Virulence
  - Drug susceptibility and resistance
  - Fitness and adaptation
  - Speciation

Protein sequences

- Most protein sequences are obtained by ‘conceptual translation’ of coding DNA.
- It is usually DNA which is sequenced in the laboratory, rather than protein.
- The protein database available via Entrez includes translations of all ‘annotated’ coding sequences in the DNA database.
- Additionally it includes **SwissProt**, a high-quality (but smaller) protein sequence database.

Protein sequence databases

- **GenPept** (via Entrez)
  - Translation of all coding sequences in Genbank
  - Automatically generated without review
  - High volume, unpredictable quality

- **Swissprot** (via Entrez and various sites)
  - Whole-genome data: genomic DNA
  - **Manually curated**
  - Low volume, high quality

Protein sequence in Fasta format

```
>gi|3605|emb|CAA31443.1| unnamed protein product [Saccharomyces cerevisiae]
MSKLGQEKNEVNYSDVREDRVVTNSTGNPINEPFVTQRIGEHGPLLLQDYNLIDSLAHFNRENIPQRNPH
AHGSGAFGYFEVTDDITDICGSAMFSKIGKRTKCLTRFSTVGGDKGSADTVRDPRGFATKFYTEEGNLDW
VYNNTPVFFIRDPSKFPHFIHTQKRNPQTNLRDADMFWDFLTTPENQVAIHQVMILFSDRGTPANYRSMH
GYSGHTYKWSNKNGDWHYVQVHIKTDQGIKNLTIEEATKIAGSNPDYCQQDLFEAIQNGNYPSWTVYIQT
MTERDAKKLPFSVFDLTKVWPQGQFPLRRVGKIVLNENPLNFFAQVEQAAFAPSTTVPYQEASADPVLQA
RLFSYADAHRYRLGPNFHQIPVNCPYASKFFNPAIRDGPMNVNGNFGSEPTYLANDKSYTYIQQDRPIQQ
HQEVWNGPAIPYHWATSPGDVDFVQARNLYRVLGKQPGQQKNLAYNIGIHVEGACPQIQQRVYDMFARVD
KGLSEAIKKVAEAKHASELSSNSKF
```

Single-letter amino acid codes

G glycine
P proline
A alanine
V valine
L leucine
I isoleucine
M methionine
C cysteine
F phenylalanine
Y tyrosine

W tryptophan
H histidine
K lysine
R arginine
Q glutamine
N asparagine
E glutamic Acid
D aspartic Acid
S serine
T threonine

Genome databases

- Contain sequence and annotation
- Often ‘better’ than general databases, for the genome in question
- There are many … for example
- Ensembl
    - http://www.ensembl.org
- Entrez Genome
    - http://www.ncbi.nlm.nih.gov/genome

Gene expression databases

- Unigene: ESTs
  - http://www.ncbi.nlm.nih.gov/unigene
- GEO
  - http://www.ncbi.nlm.nih.gov/geo

Further protein databases

- RefSeq, Integr8: genome-oriented
  - http://www.ncbi.nlm.nih.gov/RefSeq
  - http://www.ebi.ac.uk/integr8
- PDB: structures
  - http://www.rcsb.org/pdb

General course aims

- To see what bioinformatics is like.
- To reach the point where, in a biology research project, you can begin to do the necessary bioinformatics for that project.
  - Underlying principles, data, software, computers, scripting.
  - Linux operating system, command-line software, Raspberry Pi hardware.
- Enjoy!

Course contents

- Six lectures
- **Eight practicals, 2 hours each**
- One seminar

- One practical project to complete and write up in your own time (50% of final module grade)
- One exam (50% of final module grade)

Recommended reading: main journals

- ***Bioinformatics***
- *PLoS Computational Biology*
- *Nucleic Acids Research*
- *Genome Research*

Papers more than a year old in *Bioinformatics*, and more than six months old in *Genome Research*, are available free online. More recent papers in these journals often require a subscription or purchase.

PLoS Computational Biology and *Nucleic Acids Research* are open access journals, with all published content available free of charge online.

Recommended reading: main books

- **Bradnam, K. and Korf, I. (2012) *UNIX and Perl to the Rescue! A field guide for the life sciences (and other data-rich pursuits)*. (Cambridge: Cambridge University Press)**
- **Dear, P.H., editor (2007) *Bioinformatics* (Bloxham, Oxfordshire: Scion).**
- Baxevanis, A.D. and Ouellette, B.F.F., editors (2005) *Bioinformatics: A practical Guide to the Analysis of Genes and Proteins*, 3rd edition (Hoboken, New Jersey: John Wiley).
- Schwartz, R.L., Phoenix, T. and foy, b d (2008) *Learning Perl*, 5th edition (Sebastopol, California: O'Reilly).

Recommended reading: other books

- **Robbins, A. (2006).** ***UNIX in a Nutshell*****, 4th edition (Sebastopol, California: O'Reilly).**
- **Christiansen, T., foy, b d and Orwant, J. (2012)** ***Programming Perl*****, 4th edition (Sebastopol, California: O'Reilly).** [3rd ed. is also OK.]
- Brown, T.A. (2007) *Genomes*, 3rd edition (New York: Garland Science).
- Cristianini, N. and Hahn, M.W. (2007) *Introduction to Computational Genomics: A Case Studies Approach* (Cambridge: Cambridge University Press).
- Kernighan, B.W. and Pike, R. (1984) *The UNIX Programming Environment* (Englewood Cliffs: Prentice-Hall).
- Orengo, C.A., Jones, D.T. and Thornton, J.M. (2003) *Bioinformatics: Genes, Proteins and Computers* (Oxford: BIOS Scientific).
- Sunnerhagen, P. and Piškur, J., editors (2006) *Comparative Genomics: Using Fungi as Models* (Heidelberg: Springer).

Further reading for the keen: genre-defining classics

- Durbin, R., Eddy, S., Krogh, A. and Mitchison, G. (1998) *Biological Sequence Analysis: Probabilistic Models of Proteins and Nucleic Acids* (Cambridge: Cambridge University Press).
- Baldi, P. and Brunak, S. (2001) *Bioinformatics: The Machine Learning Approach*, 2nd edition (Cambridge, Massachusetts: MIT Press).
- Deonier, R.C., Tavaré, S. and Waterman, M. (2005) *Computational Genome Analysis: An Introduction* (New York: Springer).

These three books are **beyond the scope of this module**. But you may wish to take a look.
