# Supplementary material for: 4273π: Bioinformatics education on low cost ARM hardware
Source: BMC Bioinformatics. 2013 Aug 12;14:243. doi: 10.1186/1471-2105-14-243 (PMC3751261; doi:10.1186/1471-2105-14-243)
Supplement: Additional file 2 — 4273π Bioinformatics for Biologists teaching material, Version 1.01. The module handbook, lectures and practicals are included. The latest version, including Linux, software and BLAST databases, is available at the 4273π Web site [25]. [file 1471-2105-14-243-S2.zip › 4273pi_course_material/week5/practical_gene_family_evolution.pdf]

4273π Bioinformatics for Biologists

Practical, Week 5: Gene Family Evolution

Peter W.H. Holland, Department of Zoology, University of Oxford
Email peter.holland@zoo.ox.ac.uk

© 2013 Peter W.H. Holland. This is an Open Access document distributed under the terms of the Creative Commons Attribution License (http://creativecommons.org/licenses/by/2.0), which permits unrestricted use, distribution, and reproduction in any medium, provided the original work is properly cited.

4273π, Version 1.01. http://biology.st-andrews.ac.uk/cegg/4273pi.aspx

Introduction

The Emx genes are a family of homeobox genes implicated in embryogenesis, particularly in head and brain development. The first Emx gene was discovered in *Drosophila melanogaster* and called *empty spiracles* (*ems*). Soon after this initial discovery two Emx genes were discovered in mammals (humans and mice), and named *EMX1* and *EMX2* (human), or *Emx1* and *Emx2* (mouse). These two genes are also expressed in brains.

Some relevant references dealing with the early characterisation of these genes are;

Dalton, D., Chadwick, R. and McGinnis, W. (1989) Expression and embryonic function of empty spiracles: a *Drosophila* homeo box gene with two patterning functions on the anterior-posterior axis of the embryo. *Genes Dev.* 3, 1940-1956.
Holland P.W.H. (1992) Mice and flies head to head. *Nature* 358, 627-628.
Simeone A. *et al*. (1992) *Nature* 358, 687-690.
Cecchi, C. and Boncinelli, E. (2000) Emx homeogenes and mouse brain development. *Trends Neurosci.* 23, 347 -352.

For the terms paralogy, orthology and a clear definintion of relationships between duplicated genes see,

Sharman AC (1999) *Seminars in Cell and Developmental Biology* 10, 561-563.
Mendivil Ramos, O. and Ferrier DEK. (2012) *International Journal of Evolutionary Biology*, vol. 2012, article id 846421.

As with the fruit fly, *Drosophila*, most other invertebrates also have a single Emx gene. It seems logical that the mammalian genes evolved by gene duplication from a single ancestral Emx gene. The aim of this practical is to determine when and how this duplication occurred.

Imagine you have cloned two Emx genes from zebrafish. You want to know whether they are 'orthologues' of mammalian Emx1 and Emx2. We will call your zebrafish genes *EmxA* and *EmxB* to start with (not Emx1 and Emx2), just so we don't bias our views of how they relate to mammalian Emx1 and Emx2. You are going to conduct a series of tests to determine how these two zebrafish genes are related to mammalian Emx1 and Emx2. The purpose of this exercise is not only to answer this question, but also to demonstrate how the wrong conclusions can sometimes be drawn. Therefore please do the exercises in the order shown.

Here are the two zebrafish gene sequences (or more accurately the two predicted protein sequences encoded by these genes).

Zebrafish EmxA
MFQHNKKCFTIESLVGKDSNSSNAAADEPIRPTALRFTESIHPSPFGSCFQNSGRTLYS
SSPEMMFTDPSTHSTNSGLSLRHLQIPTQPFFSPHQRDTLNFYPWVLRNRYLGHRFQG
DDSSPENLLLHGPFSRKPKRIRTAFSPSQLLRLERAFEKNHYVVGAERKQLANGLCLT
ETQVKVWFQNRRTKHKRQKLEEESPDPQQKRKGSQHVSRWRVATQQGSPEDIDVIS
ED

Zebrafish EmxB
MFQPTPKRCFTIESLVAKDNPLPSSRSEEPIRPAALSYANSSQMNPFLNGFHSSGRGVY
SNPDLVFAEAVSHPPNSAVPVHSVPPPHALAAHPLSSSHSPHPLFASQQRDPSTFYPW
LIHRYRYLGHRFQGNETSPESFLLHNALARKPKRIRTAFSPSQLLRLEHAFEKNHYVV
GAERKQLAHSLSLTETQVKVWFQNRRTKFKRQKLEEEGSDSQQKKKGTHHINRWRL
ATKQGSPEEIDVTSDDVNRWRLATKQSSPEAIDVTSED

These are also given in the text file `task1.txt` in the `~/4273pi/week5` directory.

Task 1

Usually the first thing anyone does when they clone a 'new' gene is to do a BLAST search. A BLAST search is a quick way of seeing how 'similar' your test DNA or protein sequence is to all the other sequences known in the world.

BLAST is a useful, but crude, method for identifying what gene family a gene belongs to. Unfortunately many people use BLAST output as an indicator of exactly what gene they have cloned. To say that another way, BLAST is a useful method to tell you, 'Hey, your gene is a sort of Emx gene', but it is not very good at saying 'It is an Emx2' for example. In any case we will try it to see how it works in this example.

There are many ways of doing BLAST searches. Here we will use the simplest and quickest. It may well not be the best! You will use a BLAST method called BLASTP to search your two fish protein sequences against a massive database of proteins encoded by the human genome. You will use amino acid sequences for the BLAST, NOT nucleotide sequences. This is very important for cross-species comparisons.

Go to,

http://www.ncbi.nlm.nih.gov

In the 'popular resources' menu click on the 'BLAST' option. Then in the list of 'BLAST assembled genomes' click on the 'human' option. This takes you to a page where you can BLAST your query sequences against human sequences.

Near the top of the page there is a 'program' menu (set of tabs). Choose the 'BLASTP' option here. In the 'Choose search set' box check that the 'Refseq protein' is selected.

Now paste in your first zebrafish sequence, EmxA, being sure to do this in FASTA format (ask if you are not sure what this is) and hit the 'BLAST' button. Once the BLAST search is complete after several seconds the results will appear in a new page. There are several output windows in the BLAST results. The most informative one, particularly for our purposes, is the 'Alignments' window. Scroll down the page to find this window.

What is the top BLAST hit? Record its details (name, accession number, score value, Expect value, and identities value). Also make a brief note of the next 5 BLAST hits.

Now do the same for EmxB.

Once you have your BLAST results make some notes on the following questions and we will discuss them in the practical.

1) Do you think that the top BLAST match is the best match (sometimes a more significant *E* value does not equate to a longer match)?
2) Based on this BLAST information, and this information only, which zebrafish gene is an orthologue of human *EMX1* and which is an orthologue of *EMX2*?
3) Why did we use amino acid sequences rather than nucleotides?
4) What might be the problems with using BLAST for identifying the gene you have cloned?

Task 2

Since BLAST can be a poor method to determine exactly what your 'new' gene is, we need to do some more careful sequence comparisons. The next step is to build a phylogenetic tree: an evolutionary tree of the genes. If we want to know how our zebrafish genes relate to the

mouse and human genes then we need to include the genes from all of these species plus an OUTGROUP. The closest useful outgroup is likely to be amphioxus. So next we will reconstruct a phylogenetic tree using the two fish sequences, two mouse sequences, two human sequences and the one amphioxus sequence.

We will do this a quick and easy way, which is certainly not the best, most rigorous and accurate approach. It is sufficient to illustrate the principles and ideas though.

The long-winded way that we are NOT going to use would be to make a multiple alignment of all of the amino acid sequences, with a program such as MAFFT or CLUSTALW. This alignment should then be edited by eye, using editing programs such as BioEdit or Seal. At this stage ambiguously aligned regions can also be removed. The final step is to build a phylogenetic tree, ideally using the most sophisticated methods such as Maximum Likelihood or Bayesian statistics. There are various programs available to do this, such as PhyML, TREEPUZZLE and MrBayes.

For the sake of speed I have provided an edited alignment for you, `task2.txt`. We will use a quick online tree-building program (TreeTop), at

http://www.genebee.msu.su/services/phtree_reduced.html

Paste the alignment into the box, select the 'rectangular' option in the 'picture formats' menu and hit the 'submit' button. The results should come through in a matter of seconds. Scroll to the bottom of the results page to see a graphical output of the phylogenetic tree.

So now you have a phylogenetic tree, which you should use to deduce when gene duplications occurred, and which fish gene is related to which mammalian gene. You need to view your tree with AmphiEmx (amphioxus) as the most basal or outgroup sequence (like C in the examples below).

Caution: Remember that trees can be fallible and need not be read literally in every case; reconstruction programs can make mistakes; they do not tell us what actually happened in evolution. Also, remember that any node in a tree can rotate. In other words, these two example trees shown below are identical.

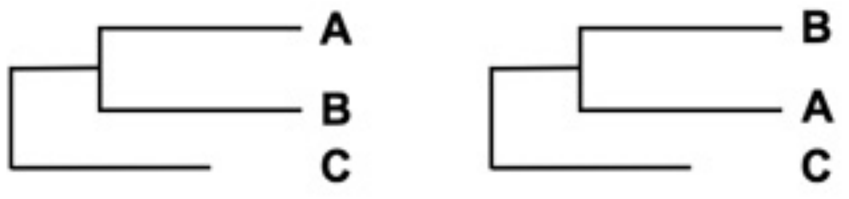

Make notes on the following, for discussion.
1) Have you changed your opinion about the identity of the two fish genes?
2) How does EmxB relate to the mammalian genes? What about EmxA?

Task 3

You may have a good idea of what EmxA and EmxB are, or you could have a confusing picture. To get more insight we will now include three additional sequences. These are three Emx genes cloned from a dogfish. So now we will reconstruct a phylogenetic tree using these three shark sequences along with all of the previous sequences.

Again I have prepared an edited alignment for you to paste into TreeTop (`task3.txt`).

Now consider the following questions.
Have you changed your opinion about the identity of the two zebrafish genes?
Is one, both, or neither zebrafish gene orthologous to the mammal genes?
How do they relate to the dogfish genes?
How many gene duplications occurred?
Does gene loss feature in the evolutionary history of these genes?

Task 4

Another important source of information that helps to clarify the modes of gene evolution is genomic location and gene neighbours. This can be considered in terms of paralogous regions within genomes and syntenic regions between genomes.

You will examine the locations in the human genome of the two human genes, EMX1 and EMX2. We will use a database called OMIM to find the neighbouring genes, and then look to see if any other regions of the human genome share any similarity. This can be a useful way to deduce gene loss. The loss of a gene was once thought to be an invisible event. Hopefully you will see that by a combination of genome scanning and molecular phylogenetics, gene loss can be detected, and more information about the pattern of gene duplication in evolutionary history discovered.

To access the OMIM database go to,

http://www.ncbi.nlm.nih.gov

Under 'Resource List', find the link to 'Online Mendelian Inherhitance in Man (OMIM)' and click it. In the search box enter 'EMX1' and hit 'go'. Under the top gene there will be a 'Gene map locus'. Make a note of it and then click on it. This takes you to an OMIM page with some tabulated information about your query gene. Click on the genomic location in this table to be taken to a genome browser. Near the top of the genome browser there are buttons

to zoom in or zoom out. Use the zoom out '10x' button two or three times so that you can now see your query gene and a decent number of its neighbours. Make a note of the neighbouring genes. Repeat this process for EMX2. Make a note of any genes that seem to be paralogous between your two searches (related to each other the same way that EMX1 and EMX2 are).

To make life easier we will concentrate on just a few of these EMX neighbours. In particular we will focus on some of the neighbouring homeobox genes. Use OMIM to find the gene map positions of VAX1 and VAX2, LBX1 and LBX2. Make a note of each of the map positions.

Now try this for a homeobox family of which humans have three members. Search for TLX1, TLX2 and TLX3.

Now add in HMX1, HMX2, MSX1 and MSX2. We can also start to extend this beyond homeobox genes. Search for FGFR1, FGFR2, FGFR3 and FGFR4 (Fibroblast Growth Factor Receptors), noting the map positions as you go.

Draw up a summary table or diagram and relate this back to the rest of the practical. One of these FGFR genes doesn't quite fit so neatly. Which one is it, and why might this be?

What does all of this tell us about EMX gene evolution?

For further background on vertebrate genome evolution and duplication, and the amphioxus Emx genes see,

Putnam, N.H. *et al*. (2008) The amphioxus genome and the evolution of the chordate karyotype. *Nature* 453, 1064-1071.
Williams, N.A. and Holland, P.W.H. (2000) An amphioxus Emx homeobox gene reveals duplication during vertebrate evolution. *Mol. Biol. Evol*. 17, 1520-1528.
Minguillón. C. *et al*. (2002) Gene duplications in the prototypical cephalochordate amphioxus. *Gene* 287, 121-128.

Acknowledgements

I thank David Ferrier (University of St Andrews) for help in testing this practical and for suggesting additional relevant references.
