# Supplementary material for: 4273π: Bioinformatics education on low cost ARM hardware
Source: BMC Bioinformatics. 2013 Aug 12;14:243. doi: 10.1186/1471-2105-14-243 (PMC3751261; doi:10.1186/1471-2105-14-243)
Supplement: Additional file 2 — 4273π Bioinformatics for Biologists teaching material, Version 1.01. The module handbook, lectures and practicals are included. The latest version, including Linux, software and BLAST databases, is available at the 4273π Web site [25]. [file 1471-2105-14-243-S2.zip › 4273pi_course_material/week5/lecture_outline_gene_family_evolution.pdf]

4273π Bioinformatics for Biologists

Lecture Outline, Week 5: Gene Family Evolution

David E.K. Ferrier, School of Biology, University of St Andrews
Email dekf@st-andrews.ac.uk

© 2013 D.E.K. Ferrier. This is an Open Access document distributed under the terms of the Creative Commons Attribution License (http://creativecommons.org/licenses/by/2.0), which permits unrestricted use, distribution, and reproduction in any medium, provided the original work is properly cited.

4273π, Version 1.01. http://eggg.st-andrews.ac.uk/4273pi

Note. This outline includes notes and references that could be used to create a set of slides. To allow use of the Creative Commons Attribution License, actual slides for this lecture are not distributed with 4293π.

1. - Emphasis on sorts of bioinformatics that being done and the data available rather than particular computer programs.
- How problems approached and the sort of data needed for advances.

2. Largely concentrate on homeobox genes as an ideal example of a gene family.

Homeobox was first discovered in the genes of the fly Hox cluster.
Genome organisation of importance to evo-devo.
Colinearity – rather mysterious phenomenon.

3. Loads of homeobox genes in animals (& plants & fungi & protists).
2 main animal classes – ANTP and PRD.
Galliot, B., de Vargas, C. & Miller, D. (1999) *Dev. Genes Evol*. 209, 186-197

Sequence similarity--> origin by duplication

4. 133 homeobox genes in amphioxus
11 classes - not absolutely based on phylogeny (use other motifs)
Phylogenetic tree of all amphioxus homeobox genes.
From Takatori *et al*. (2008) *Dev. Genes Evol.* 218, 579-590.

5. Developmental control genes expanding during animal evolution
(but be aware of independent duplications/expansions, and gene losses).

Independent expansions of homeoboxes in animals, plants & fungi.

Table of, ANTP-class homeobox gene number in animal genomes.

= Table 2 from,
Ferrier, D.E.K. (2009) Evolution of Hox complexes. In *Hox gene studies from the 20th to the 21st century*, Ed. J. Deutsch. Advances in experimental medicine and biology, vol. 689. Landes Bioscience.

6. How can duplicated genes be retained?
- The DDC model
Figure adapted from Fig.1 of Force *et al*. (1999) *Genetics* 151, 1531-1545.

7. Example of DDC.
Mouse and zebrafish Hoxb1 genes,
Fig.2 from Prince & Pickett (2002) *Nature Reviews Genetics* 3, 827-837.

8. Terminology for duplicated genes.
(From Sharman (1999) *Seminars in Cell & Developmental Biology* 10, 561-563.)
Pro-orthologue - a gene that is orthologous to the ancestor of the whole set of paralogues of the gene in question.

Semi-orthologue - One of the descendants of an orthologue of the gene in question, after that gene has duplicated.

Trans-homologue - one of the duplicated genes descended by one lineage from an ancestral gene, another descendent of which also duplicated on a different lineage to give the gene in question and its paralogues.

For a more extensive review of terminology see,
O. Mendivil Ramos & DEK Ferrier (2012) *Int. J. Evol. Biol.*, article id 846421
doi: 10.1155/2012/846421

9. Choosing taxa at key phylogenetic positions
Figure of animal phylogeny.
- amphioxus and chordate evolution.

10. Amphioxus
- (*Branchiostoma floridae*), a key taxon for understanding deuterostome, chordate and vertebrate evolution.
- Basal lineage of chordates.
- Genome sequenced.

11. Deuterostome phylogeny, with whole genome duplication at the origin of vertebrates (2R) and then again at the base of the teleosts (3R). Also, widespread genome rearrangements in urochordates.

Amphioxus = basal lineage chordate.
Less derived genome with regards to duplications and rearrangements.
- important when trying to understand ancestral genome organisation as with homeobox genes.

12. The prototypical Hox cluster of amphioxus.
Amphioxus has retained both homeobox genes and homeobox gene clusters.

Ferrier et al. (2000) *Evol. & Devel.* 2, 284-293.
Powers & Amemiya (2004) *Curr. Biol.* 14, R183-4.
Kuraku et al. (2008) *PNAS* 105, 6679-6683.
Figure adapted from Ferrier (2004) *Curr. Biol.* 14, R210-211

AmphiHox14 = first ever Hox14 gene. Where from? Not obviously duplicated from any particular other posterior Hox gene.
AmphiHox14 was precursor to discovery of vertebrate Hox14 genes. So gene loss important in tetrapods. But still not clear orthology to AmphiHox14 from trees.
Now agnathan Hox14, but still some ambiguity!
Gene loss important, & taxon sampling and new gene discovery can drastically change views.

13. The Whole Genome of amphioxus, and the complete amphioxus Hox cluster.
L.Z.Holland *et al.* (2008) *Genome Research* 18, 1100-1111.

Now AmphiHox15. Only now we have complete picture of cluster composition.
Gene orientations all the same and cluster is 'clean' of non-Hox genes.
One of the very few prototypical Hox clusters available.
Cautionary tale- don't take genome projects at face value!
Double-checks (e.g. chromosome Fluorescent In Situ Hybridisation (FISH)).

14. Hox = one of most striking examples for 2R (& 3R).

Few clusters characterised, & many of those very derived (broken & shuffled).
Some genomes are less derived than others.
Gene loss extensive.
Fig2 from Lemons & McGinnis (2006) *Science* 313, 1918-22.

15. Amphioxus has a prototypical chordate genome.
All of the homeobox families present in the last common ancestor are still present in the amphioxus lineage.

Takatori et al (2008) *Dev. Genes Evol.* 218, 579-590
and
Fig.3 from, Holland *et al.* (2008) *Genome Res*. 18, 1100-1111.

Method - careful, iterative tBLASTn searches, with catalogues built from several genomes, encompassing the most divergent members of the gene family.

16. Supplementary Fig.64 Dot plot of orthologous gene pair locations in amphioxus versus human.
From, Putnam, NH. et al. (2008) *Nature* 453, 1064-1071.

Whole genome comparisons between 'less-derived' genomes. The use of synteny mapping.

There are17 ancestral Chordate Linkage Groups (CLGs).

17. Quadruple conserved synteny.
Along the 17 CLGs, they usually match to 4 regions of the human genome (& chicken).

Fig.3 from Putnam *et al*. (2008) *Nature* 453, 1064-1071.

18. Synteny and reconstruction of ancestral genomes.

The example of the amphioxus ParaHox gene cluster
Figures adapted from,
Brooke *et al*. (1998) *Nature* 392, 920-922. &
Ferrier *et al*. (2005). *Current Biology* 15, R820-2
Ferrier & Holland (2001). *Nature Reviews Genetics* 2, 33-38.

- Sister to Hox (deduced from gene phylogenies & expression → Colinearity).
- Conserved to mammals.

19. Synteny reveals chordate ParaHox evolution via whole cluster duplication and gene loss.

Fig.2 from, Ferrier *et al*. (2005) *Curr Biol* 15:R820-R822

- Clusters have been dismantled, by gene loss,
- But 1 has been conserved in tetrapods... conserved due to constraint??

(Genes can move away, and can move into paralogons, as well as being lost).
~Jigsaw puzzle

20. Choosing taxa at key phylogenetic positions
Animal phylogeny figure.
Polychaetes to go deeper into animal ancestry. Towards the Urbilaterian.

21. Reconstruction of the Urbilaterian genome (around the ParaHox cluster).

Figure adapted from Fig.2 of Hui *et al*. (2009) *BMC Biology* 7:43

We can start reconstructing the genome of the ancestral bilaterian around this important cluster of developmental control genes.

Gene model prediction (Fgenes, Genscan, genewise...), staggered windows & tBLASTx --> sequence align & phylogenies to decide on which particular genes are the human orthologues (incl. paralogues).

22. Reconstruction of the Urbilaterian genome (around the ParaHox cluster).

Figure adapted from Fig.5 from, Hui et al (2009) *BMC Biology* 7:43
Artificial amalgam of Platynereis genomic contigs.
(Gene neighbour order not significant).
Logic = if genes neighbours in two lineages, then neighbours in ancestor.
Polychaetes less derived --> more robust reconstruction of starting point for diversification

of bilaterian animals.

23. Choosing taxa at key phylogenetic positions
Going deeper (non-bilaterians).

24. Sea anemone genome reveals ancestral eumetazoan gene repertoire and genomic organization.
Fig.3B from, N.H.Putnam et al. (2007) *Science* 317, 86-94.

Whole genome sequence. Synteny analyses mapping whole scaffolds to entire human chromosomes.
But didn't find a ParaHox cluster!
From ProtoHox hypothesis, anything with Hox should have ParaHox.
- and controversy as to whether Cnidaria do contain Hox (& ParaHox) genes.
Phylogenetics is suggestive, but ambiguous.

25. Cnidarians do have a ParaHox locus.
Figure adapted from Fig.2 from, Hui, Holland & Ferrier (2008) *Evol. Devel.* 10:725-730.

scaffold 27 = 1.5Mb -->33 genes with clear human orthologues (=68 human genes, incl. paralogues).
No statistically significant linkage with a human ParaHox chr. (cf. Putnam *et al.* tendency for scaffold 27 genes to be syntenic with human chr.15).
But 115kb region is now statistically significant. SCALE of analysis crucial
Also carries through to lophotrochozoan genomes (scaffold numbers).

Refutes cnidarian independent duplication hypothesis

26. And if you want to go further...
see, O. Mendivil Ramos, D. Barker & D.E.K. Ferrier (2012)
Ghost loci imply Hox and ParaHox existence in the last common ancestor of animals.
Current Biology 22, 1951-1956.

27. Origin of the ANTP class of homeobox genes via a Mega-cluster.
Fig3 from, Garcia-Fernàndez (2005) *Nature Reviews Genetics* 6, 881-892.
Adapted from Pollard and Holland (2000). *Current Biology* 10, 1059-1062.

ANTP class. Presumed origins via tandem duplications.
Jigsaw puzzle between genomes, and between paralogy regions in vertebrates.
Splits along divergent lineages - but when & how?
What remained of the Mega-cluster in the ancestral bilaterian (from which most of the animal kingdom subsequently evolved)?

28. Genomic jigsaw puzzles to reconstruct ancestral states.

Fig1 From Pollard and Holland (2000). *Current Biology* 10, 1059-1062.

29. Using paralogy to reconstruct ancestral states.
Fig.2 from, Pollard and Holland (2000). *Current Biology* 10, 1059-1062.
Paralogues.

Overlapping domains - jigsaw.
Paralogous non-homeobox genes linked to Hox, EHGbox, NKL and ParaHox gene clusters in humans.

30. The ancestral Mega-homeobox gene cluster
The hypothesis:
Figure adapted from Fig3 from, Pollard and Holland (2000).
Current Biology 10, 1059-1062.

Context of Mega cluster.
Tandem duplication, gene phylogenies and genomic neighbours.

31. Homeoboxes in the genome of *Tribolium castaneum*.
Red Flour Beetle.
Relatively non-derived genome - homeobox content - Hox cluster.

32. The Urbilaterian Super-Hox cluster
Fig.1 from Butts, Holland & Ferrier (2008) *Trends Genet.* 24, 259-262
Component of Mega cluster in bilaterian ancestor?
Assumption - tight linkage/clustering is inherited from ancestor, not secondary coming together.

For update on Mega-cluster hypothesis see,
Hui, J.H.L.et al. (2012) *Molecular Biology and Evolution* 29, 157-165.

33. The Last Common Eubilaterian (LCE) (>550MYA)

From Hox (& ParaHox) cluster,
to homeobox content as a whole (& gene loss),
to the SuperHox cluster, and remains of the Mega cluster in the LCE,

to evolutionary dynamics of animal genomes.

This pan-bilaterian comparative genomics permits reconstruction of this ancestor's genome, and by extension with respect to clustered homeobox genes, the ancestral developmental mechanisms.

We do not yet know enough about nature and rates of genome evolution across animals (translocations, fusions, fissions) to distinguish constrained, functionally important regions from evolutionary relics.
