# Supplementary material for: 4273π: Bioinformatics education on low cost ARM hardware
Source: BMC Bioinformatics. 2013 Aug 12;14:243. doi: 10.1186/1471-2105-14-243 (PMC3751261; doi:10.1186/1471-2105-14-243)
Supplement: Additional file 2 — 4273π Bioinformatics for Biologists teaching material, Version 1.01. The module handbook, lectures and practicals are included. The latest version, including Linux, software and BLAST databases, is available at the 4273π Web site [25]. [file 1471-2105-14-243-S2.zip › 4273pi_course_material/week7/practical_PAML.pdf]

4273π Bioinformatics for Biologists

Practical, Week 7: PAML

Daniel Barker, School of Biology, University of St Andrews
Email db60@st-andrews.ac.uk

© 2013 D. Barker. This is an Open Access document distributed under the terms of the Creative Commons Attribution License (http://creativecommons.org/licenses/by/2.0), which permits unrestricted use, distribution, and reproduction in any medium, provided the original work is properly cited.

4273π, Version 1.01. http://eggg.st-andrews.ac.uk/4273pi

The learning objectives are:

- To consolidate your theoretical understanding of $\omega = d_N / d_S$.
- To learn how to use PAML software to estimate ω from a multiple alignment and phylogeny of sequences.
- Revision: likelihood and log likelihood.
- Use of the likelihood ratio test, to obtain p-values from log likelihoods.

Before the practical class, in your own time: Download and Install PAML

If completed correctly first time, this procedure takes approximately an hour. Please complete it before the practical class.

Go to:

http://abacus.gene.ucl.ac.uk/software/paml.html

Click the 'Downloading and setting up PAML' link, and download `paml4.7.tgz`. Save it in the `~/4273pi/week7/` directory. At the command prompt in LXTerminal, change to this directory. Then, uncompress PAML:

```
tar xfz paml4.7.tgz
```

The software is provided as source code in the C programming language, which has to be compiled for use. In the case of PAML, as with SNAP in Week 6, this is achieved conveniently by `make`. Enter the following commands (based on instructions on the PAML Web site):

```
cd paml4.7
rm bin/*.exe
cd src
make -f Makefile
```

Output will appear on screen, which we will ignore. When the command prompt returns, move the executable files into a sensible location:

```
mv baseml basemlg chi2 codeml evolver mcmctree pamp yn00 ../bin
```

Any error message at this point indicates something has gone wrong – in which case you might delete `~/4273pi/week7/` and begin again with the `tar` command, above.

Launch a PAML analysis to be interpreted later

To avoid waiting, we will launch a PAML analysis right now, without any explanation. By the time it completes, you should be ready to interpret its results.

Open an LXTerminal and change directory to `~/4273pi/week7/paml4.7/examples/HIVNSsites`. Run PAML's `codeml` program, as follows:

```
~/4273pi/week7/paml4.7/bin/codeml codeml.ctl
```

There should be no error message, and output should start to appear on the screen.

Open another terminal window and take a look at `README.txt` in this same directory.

Introduction

This practical session involves using the PAML program (Yang 2007). PAML can do many things, and is excellent for studies of synonymous and nonsynonymous substitution ratios (Yang 2006, Chapter 8). It obtains these from a multiple alignment of codons, and a phylogenetic tree for the sequences in that alignment.

As you will remember from this week's lecture, ω is the ratio of the normalized nonsynonymous substitution rate d_N to the normalized synonymous substitution rate d_S for protein-coding sequences, i.e.

$$\omega = d_N / d_S \qquad (1)$$

Substitution mutations may happen at roughly the same rate across the genome, but not all of these will be observed in extant organisms. Some substitutions will reduce fitness, and

tend to be eliminated from the gene pool; a few will increase fitness, and tend to spread throughout the gene pool; and some will have little or no effect on fitness and will persist, or not, depending on other factors (e.g. genetic drift).

Values of ω for a gene, or for part of a gene-family phylogeny, or for part of a gene tell us something about how evolution has been occurring in that gene, or that part of the phylogeny, or that part of the gene (Table 1).

**Kind of evolution**	ω
Neutral evolution	1
Purifying selection	< 1
Positive selection	> 1

Table 1. Expected values of ω for different kinds of evolution in a protein-coding sequence.

In *neutral evolution*, nonsynonymous mutations are observed in sequences as often as would be expected by chance. This is highly unlikely in functional coding sequence, and might suggest that our 'protein-coding sequence' is in fact from a pseudogene.

For protein-coding sequence, *purifying selection* is the normal state of affairs. In purifying selection, nonsynonymous mutations are observed less often than would be expected by chance. In other words, there is selective pressure for the amino acid sequence to remain the same. Presumably the mutations are happening at the same rate as in neutrally evolving sequences, but tend to reduce the fitness of the organism, hence tend to be eliminated from the gene pool over time.

Positive selection (adaptive evolution) is an interesting state of affairs. Here, natural selection is acting to favour the survival of nonsynonymous mutations. Genes under positive selection are often involved in interactions between species or individuals; for example mate recognition, or an 'arms race' between the immune system and pathogens.

→ **QUESTION 1**. Why, for protein-coding sequences in general, is purifying selection ($\omega < 1$) the typical state of affairs?

→ **QUESTION 2**. Why might some pathogen sequences be evolving under positive selection ($\omega > 1$)?

PAML is a complex program which can do many things. You will find its documentation in `~/4273pi/week7/paml4.7/doc`. Take a quick look at the manual.

Background

Site models in PAML allow ω to vary among sites (codons). By comparing the ability of different models to explain our observed sequences, we may test hypotheses such as whether or not a given gene is under positive selection. If a gene *is* under positive

selection, the models help us find out which specific sites within the gene are evolving in this way.

PAML model M1a is known as the *nearly-neutral* model. In model M1a, there are two possible values of ω allowed for sites: a value estimated from the data $0 < \omega_0 < 1$, and a fixed value $\omega_1 = 1$. PAML model M2a is known as the *selection* model. Model M2a allows three possible values of ω: $0 < \omega_0 < 1$, $\omega_1 = 1$, and $\omega_2 > 1$.

If our sequences are evolving without positive selection in any of their sites, model M1a may be thought of as a reasonable approximation of reality. Certainly, for such sequences M2a is not expected to explain our data significantly better than model M1a, because in reality no sites actually do have $\omega > 1$. But if some sites *are* evolving under positive selection, model M2a *could* explain our sequences significantly better than model M1a, because allowing $\omega > 1$ in the model will more closely match the underlying biological reality.

When PAML fits a model, it optimizes the values of ω_0 and (if present in the model) ω_2. It gives a log likelihood for the model.

For a given model structure and given data *D*, and the values for the model parameters constituting a hypothesis *H*, likelihood *L* is proportional to the probability of the observed data *D*, if *H* were correct. I.e., $L = kP(D \mid H)$. *L* is a function of the parameter values *H* and is only defined up to an arbitrary positive constant, *k*, which may be assumed to be a property of the data at hand. (This means that likelihoods obtained for different data cannot be compared.)

In practice, *L* tends to be obtained as a number very close to zero. Problems can occur when computers handle very small numbers. So we usually use its natural logarithm, or *log likelihood*. Log likelihood may be written $\ln L$ or ℓ. One may convert ℓ to *L* in the usual way:

$$L = e^{\ell} = \exp(\ell) \qquad (2)$$

For a given set of observed data, a higher *L*, hence a more positive ℓ, indicates a better fit between the hypothesis and the data. When comparing log likelihoods, then, a more negative ℓ indicates a worse explanation of the observed data. All else being equal, we might prefer the hypothesis with the most positive ℓ.

There is a difficulty, though, in that hypotheses with larger numbers of free parameters will always describe the data better than (or at least as well as) simpler models. In the phylogeny reconstruction practical, you used a model selection criterion (e.g. BIC) to decide the appropriate model. Model selection criteria involve an expectation that a parameter has to be truly useful to be included in the final model, with 'truly useful' indicating that it has to increase log likelihood beyond some minimum threshold. Where one hypothesis is a null hypothesis and the other is an alternative hypothesis, model selection may be done; but it may, instead, be more useful to obtain a *p*-value and interpret it in the conventional way. One popular mechanism to obtain a *p*-value from log likelihoods is a *likelihood ratio test*.

First, some background. Where one hypothesis may be regarded as a 'special case' of another, the hypotheses are said to be described by *hierarchical models*. Only hierarchical models are suitable for a likelihood ratio test. For example, PAML model M1a may be regarded as a special case of M2a in which ω_2 is constrained to equal 1. The 'special case' model (e.g. M1a) represents our *null hypothesis* and the other, more general model (M2a) represents our *alternative hypothesis*. The alternative hypothesis always has a larger number of free parameters. The likelihood ratio test seeks to discover whether addition of these parameters significantly increases the fit between the model and reality, for the data at hand. Compared to the AIC model selection criterion, a likelihood ratio test is stricter about the relevance of additional parameters.

The log likelihood of the null hypothesis may be written ℓ_0, and the likelihood of the alternative hypothesis, ℓ_1. The *likelihood ratio statistic*, $2\Delta\ell$, is then:

$$2\Delta\ell = 2\ln(L_1 / L_0) = 2(\ell_1 - \ell_0). \qquad (3)$$

Because it is impossible for the fit of the more general model to be worse than the fit of the simpler model, L_1 / L_0 is never less than 1. Consequently, $2\Delta\ell$ is never less than 0. $2\Delta\ell$ represents the strength of evidence for the alternative hypothesis compared to the null hypothesis. At one extreme, $2\Delta\ell = 0$ indicates there is *no* evidence for the alternative hypothesis compared to the alternative hypothesis. Higher $2\Delta\ell$ indicates increasingly strong evidence for the alternative hypothesis *vs* the null hypothesis.

Making some assumptions the details of which are beyond the scope of this module (Wilks 1938), *if* the null hypothesis were true, we would expect $2\Delta\ell$ to follow a χ^2 distribution with degrees of freedom equal to the difference in number of free parameters between the alternative model and the null model. (If the alternative hypothesis were true, the shape of the χ^2 distribution would be the same, but its location would be shifted towards the right.) This assumption about the distribution under the null hypothesis allows us to convert $2\Delta\ell$ to a *p*-value, using standard statistics software. On the Raspberry Pi, you may use R. If, for example, it happened that $2\Delta\ell = 5.7$ and the alternative model had one more free parameter than the null model, you could start R:

```
R
```

Then request the *p*-value corresponding to $\chi^2 = 5.7$ when there is one degree of freedom:

```
1 - pchisq(5.7, 1)
```

Then exit R by pressing `CTRL-D` and entering `n` when asked if you want to save the workspace.

R would report that $p = 0.01696491$.

→ **QUESTION 3**. Check this by running R yourself.

This *p*-value is the probability of observing $2\Delta\ell$ at least as great as the one we did observe, *if the null hypothesis were true*. If $p < 0.05$, we would conventionally conclude that there *is* convincing evidence for the alternative hypothesis.

In general, to convert $2\Delta\ell$ to a *p*-value in R, use this R command:

`1 - pchisq(`$2\Delta\ell$`,` *df*`)`

Where *df* gives the degrees of freedom.

This procedure is widely used in biology. It may be summarized, in a practical and general way, as follows:

1) define your question in terms of comparing the relative ability of two hierarchical models, a null model (special case) with an alternative model (more general case), to explain the observed data;
2) for the observed data, use whatever software it takes, to obtain the log likelihood for the null model (ℓ_0) and the log likelihood for the alternative model (ℓ_1);
3) use simple arithmetic to calculate the likelihood ratio statistic, $2\Delta\ell$;
4) use a statistics package to convert $2\Delta\ell$ to a *p*-value by finding the probability of $\chi^2 \geq 2\Delta\ell$, when degrees of freedom equal the difference in number of free parameters between the alternative and null models.

The HIV protein coat

You are now ready to examine the test for positive selection in the V3 region of the HIV-1 *env* gene, which you launched with PAML's `codeml` program at the start of the practical. *env* codes for the protein coat of the HIV virus.

→ **QUESTION 4**. Why might the gene coding for the coat of a virus be under positive selection?

→ **QUESTION 5**. If the coat of a virus were under positive selection, what implications would this have for the development of drugs to fight the virus?

PAML analyses are configured by means of *control files*, which are just text files containing specific instructions to set up the PAML analysis. These are difficult to get right but fortunately, one has been prepared already, as part of the PAML package. You have used one already, when you launched the `codeml` command earlier: it is `codeml.ctl` in the directory `~/4273pi/week7/paml4.7/examples/HIVNSsites`.

Open an LXTerminal and change directory to `~/4273pi/week7/paml4.7/examples/HIVNSsites`. Take a look at the control file. Particularly note the lines

```
model = 0
```

and

```
NSsites = 0 1 2
```

→ **QUESTION 6**. What do these lines cause PAML to do? (See the `codeml` section of the PAML manual `pamlDOC.pdf`, particularly its Table 2 and the paragraph beginning 'The *site models* …', on p. 30.)

→ **QUESTION 7**. With this control file, which PAML model will represent our null hypothesis and which will represent our alternative hypothesis?

Also look at the other two files, `HIVenvSweden.txt` and `HIVenvSweden.trees`.

→ **QUESTION 8**. What do these files seem to contain?

As well as output to the screen, the important output from `codeml` also gets written to a text file, which will be called `mlc` (as requested in the control file `codeml.ctl`). You can find the crucial log likelihoods for the three models fitted, by using the `Search` menu in `nedit` to `Find` (and `Find Again`) the following:

```
lnL
```

This gives the log likelihood ℓ, which will be a negative number. The model is given in a heading, a few lines above. A few lines below, the values of parameters such as ω that PAML estimated, for this model, are shown under the heading `Detailed output identifying parameters`.

→ Use this handout and/or the `codeml` section of the PAML manual to interpret the `codeml` output to obtain contents for cells of Table 2.

**Model**	**Number of values of ω allowed**	ℓ	**Estimate(s) for ω parameter(s)**
M1a			
M2a			

Table 2. Characteristics of PAML M1a and M2a, their log likelihoods and fitted values of $\omega = d_N / d_S$ for the HIV-1 *env* sequence case study.

→ **QUESTION 9**. Which of the two models from Table 2 most successfully explains the data?

→ **QUESTION 10**. On purely statistical criteria (without considering the biology), is this a surprise, or not?

→ **QUESTION 11**. What is $2\Delta\ell$ for a comparison of the alternative hypothesis represented by M2a *vs* the null hypothesis M1a, for these data? (Use Equation 3 and your log likelihoods for Table 2.)

→ **QUESTION 12**. What are the appropriate degrees of freedom (d.f.) for converting this $2\Delta\ell$ to a *p*-value? (See the `codeml` section of the PAML manual).

→ **QUESTION 13**. Use R to obtain a *p*-value from your $2\Delta\ell$ and d.f.

→ **QUESTION 14**. Is this *p*-value significant?

→ **QUESTION 15**. What do you conclude about the evolution of the V3 region of the *env* gene in HIV-1?

If positive selection has been detected in a gene, it is of interest to discover which specific codons are affected. One approach is to plot a 'sliding window' of $\omega = d_N / d_S$ along the length of the coding region (e.g. Steinway et al. 2010). The alternative approach, taken by PAML, is to extract the probability of being under positive selection, for each codon. Positively-selected codons are not necessarily adjacent.

Scroll further down `mlc`. You will see `Naive Empirical Bayes (NEB)` analysis and below it, `Bayes Empirical Bayes (BEB)`. These are two broadly similar procedures, to find which specific codons show strongest evidence of positive selection. For the few sites apparently under strongest positive selection, the output at this point shows the probability that $\omega > 1$ at each site. As a 'rule of thumb', in a gene predicted to be under selection by a likelihood ratio test, sites with a probability of $\omega > 1$ exceeding 95% are those we would suspect of being under positive selection.

→ **QUESTION 16**. Which specific sites seem to be under positive selection, according to the naive empirical Bayes and Bayes empirical Bayes results?

→ **QUESTION 17**. The M2a *vs* M1a likelihood ratio test is only one possible test for positive selection. Look at the `codeml` section of the PAML manual. Copy the control file `codeml.ctl`, and modify it to perform a different likelihood ratio test for positive selection. Does it agree with the results of the M2a *vs* M1a test?

→ **QUESTION 18 (optional)**. Gather information from the scientific literature, and sequence and structure databases, to investigate the biological significance of the sites which your various analyses suggest are under positive selection.

→ **QUESTION 19 (in your own time)**. Use your own multiple alignment of of homologous proteins from several different species, obtained in the practical in Week 3. Download the nucleotide coding sequence (CDS) coding for each of the proteins you included in the multiple alignment. Convert the protein multiple alignment to a nucleotide multiple alignment, using the protein multiple alignment and unaligned nucleotides as input to `tranalign` in the EMBOSS package (which is installed on your Raspberry Pi). Test for positive selection by running `codeml` on the nucleotide multiple alignment and the phylogenetic tree you reconstructed (Practical 3). Note, PAML's tree and multiple alignment formats are a bit unusual. You will have to edit your tree and multiple

alignment (e.g. in `nedit`). Use the format of `HIVenvSweden.txt` and `HIVenvSweden.trees`, or the PAML manual, as a guide.

Acknowledgements

The HIV data are from one of the examples supplied with PAML, and were analysed by Yang et al. (2000). I am grateful to Jon Bennett (St Paul's School) for sharing with me teaching material, upon which this practical is based.

References

Steinway SN, Dannenfelser R, Laucius CD, Hayes JE and Nayak S (2010) JCoDA: a tool for detecting evolutionary selection. *BMC Bioinformatics* 11: 284.

Wilks, SS (1938) The large-sample distribution of the likelihood ratio for testing composite hypotheses. *Annals of Mathematical Statistics* 9: 60-62.

Yang, Z (2007) PAML 4: Phylogenetic analysis by maximum likelihood. *Molecular Biology and Evolution* 24: 1586-1591.

Yang, Z (2006) *Computational Molecular Evolution* (Oxford: Oxford University Press).

Yang, Z, Nielsen, R, Goldman, N and Pedersen, A-MK (2000) Codon-substitution models for heterogeneous selection pressure at amino acid sites. *Genetics* 155: 431-449.
