# Supplementary material for: 4273π: Bioinformatics education on low cost ARM hardware
Source: BMC Bioinformatics. 2013 Aug 12;14:243. doi: 10.1186/1471-2105-14-243 (PMC3751261; doi:10.1186/1471-2105-14-243)
Supplement: Additional file 2 — 4273π Bioinformatics for Biologists teaching material, Version 1.01. The module handbook, lectures and practicals are included. The latest version, including Linux, software and BLAST databases, is available at the 4273π Web site [25]. [file 1471-2105-14-243-S2.zip › 4273pi_course_material/week7/lecture_species_differences.pdf]

Looking at Species Differences

4273π Bioinformatics for Biologists
Lecture, Week 7

Michael G. Ritchie, School of Biology, University of St Andrews
Email mgr@st-andrews.ac.uk

© 2013 M.G. Ritchie. This is an Open Access document distributed under the terms of the Creative Commons Attribution License (http://creativecommons.org/licenses/by/2.0), which permits unrestricted use, distribution, and reproduction in any medium, provided the original work is properly cited.

4273π, Version 1.01. http://eggg.st-andrews.ac.uk/4273pi

How many genes do you have to change to produce a new species?

Wu & Hollocher (1998)

Humans and chimps differ by 1.7%

≈ 50 million base pairs (0.017 x $3x10^9$)

- are these all important? If only 1%, that is still 0.5 M bp

"our ignorance about the genetics of species differences spans many orders of magnitude"

SOURCES OF GENETIC VARIATION
at the population level

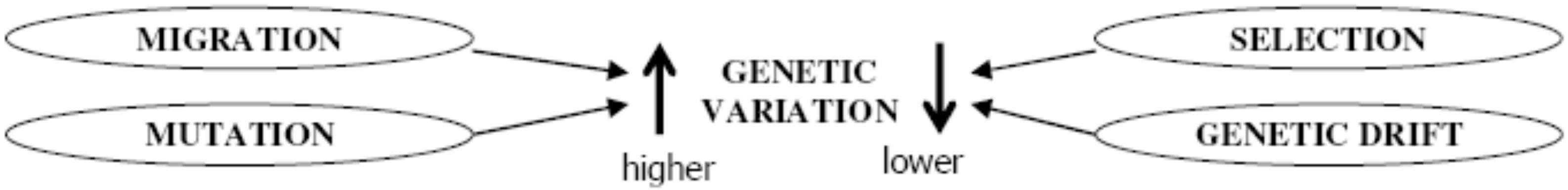

Simplest model of change: assume no immigration and no selection

Mutations: most neutral or deleterious
-> most observed variation is neutral
(Kimura's neutral theory)

Drift: main source of evolutionary change
by fixation or removal of mutations

Selection: acts to increase the frequency
of advantageous alleles

Neutral evolution (drift) of sequence – observed pattern:

fitness

ACCTCTTTAGGTACCTCTGTAGGTACCAAGTGTACCCTAG
ACCTCTTTAGGTACCTCTGTAGGTACCAAGTGTACCCTAG
ACCTCTTTAGGTACCTCTGTAGGTACCAAGTGTACCCTAG
ACCTCTTTAGGTACCTCTGTAGGTACCAAGTGTACCCTAG

↓

ACCTCTTTAGGTACCTCTGTAGGT**T**CCAAGTGTACCCTAG
ACCTC**C**TTAGGTACCTCTGTAGGTACCAAGTGTACCCTAG
ACCTCTTTAGGTACCT**A**TGTAGGTACCAAGTGTACCCTA**T**
AC**G**TCTTTAGGTACCTCTGTAGGTACCAAGTGTACCCTAG

The neutrality theory provides a “null hypothesis,” for measuring molecular evolution.

Adding positive selection:

What if one of the new mutations is beneficial
-> increases fitness of the individual?

fitness

ACCTCTTTAGGTACCTCTGTAAGTTCCAAGTGTACCCTAG
ACCTCCTTAGGTACCTCTGTAGGTACCAAGTGTACCCTAG
ACCTCTTTAGGTACCTATGTAGGTACCAAGTGTACCCTAT
ACGTCTTTAGGTACCTCTGTAGGTACCAAGTGTACCCTAG

selective sweep

ACCTCTTTAGGTACCTCTGTAAGTTCCAAGTGTACCCTAG
ACCTCTTTAGGTACCTCTGTAAGTTCCAAGTGTACCCTAG
ACCTCTTTAGGTACCTCTGTAAGTTCCAAGTGTACCCTAG
ACCTCTTTAGGTACCTCTGTAAGTTCCAAGTGTACCCTAG

hitchhiking

variation recovery

ACCTCTTTAGGTACCTCTGTAAGTTCCAAGTGTACCCTAG
ACCTCTTCAGGTACCTCTGTAAGTTCCAAGTGTACCCTAG
ACCTCTTTAGGTACCTCTGTAAGTTCCAAGTGTACGCTAG
ACCTCTTTAGGTACCTCTGTAAGTTCCAAGTGTACCCTAG

reduction of variability, local linkage

Why look for genes that diverge due to selection rather than drift?

- To test evolutionary theory
- To identify functional elements in genomes
- To identify genes showing adaptive divergence between species

Many genes and gene regions are conserved across diverse groups of species, and these typically have high functional importance (change is constrained) – detecting these is an important task.

Evolutionary biologists are more interested in detecting positive selection because these genes/regions may be responsible for evolutionary innovations and species divergence

Two major types of selection

- Positive or diversifying selection
  - leads to adaptive substitutions

- Negative or purifying selection
  - gets rid of deleterious mutations or low-frequency genes

Both reduce genetic diversity – so how do we detect them?

$\omega = d_N / d_S$

d_N = Non-synonymous substitution rate

d_S = Synonymous substitution rate

AAC -> plasticene

AAT -> plasticene

} synonymous

AAG -> playdoh - non-synonymous

If genes are evolving neutrally, synonymous/non-synonymous rate (ω) should be constant.

If an excess of non-synonymous ($\omega > 1$) => diversifying selection

If an excess of synonymous ($\omega < 1$) => purifying selection

Substitution rate variation and selection

Phylogenetic comparison of
synonymous (d_s)
and
nonsynonymous (d_n)
substitution rates ratio (ω): $\omega = d_n/d_s$

$d_s > d_n$	$d_s = d_n$	$d_s < d_n$
⇩	⇩	⇩
**$\omega < 1$**	**$\omega = 1$**	**$\omega > 1$**
⇩	⇩	⇩
negative (purifying) selection	neutral evolution	positive (diversifying) selection

PAML – what can it do?

PAML implements a number of sophisticated models,
which can be used to construct likelihood ratio tests of evolutionary hypotheses

Features include:

- estimating synonymous and nonsynonymous rates
- testing hypotheses concerning *d*N/*d*S rate ratios
- various amino acid-based likelihood analysis
- ancestral sequence reconstruction (DNA, codon, or AAs)
- various clock models
- simulating nucleotide, codon, or AA sequence data sets
- and more ……

Likelihood Ratio Test of sites under positive selection

If there two alternative models describing the dataset:

H_0: there are no sites at which $\omega > 1$

H_1: there are such sites (under selection)

To compare two models:

Calculate $2\Delta l = 2(l_1 - l_0)$

and compare it with a X^2 distribution

A gene is considered to have evolved under positive selective pressure if:
1. the LRT is significant
2. at least one of the ML estimates of $\omega > 1$.

<u>**References & case studies**</u>

Wu, C.-I. & Hollocher, H. 1998. Subtle is nature: the genetics of species differentiation and speciation. In: *Endless forms: species and speciation* (Ed. by D. A. Howard & S. H. Berlocher), pp. 339-351. Oxford: O. U. P.
Essay on species differences

Yang, Z. H. 1997. PAML: a program package for phylogenetic analysis by maximum likelihood. *Computer Applications in the Biosciences*, 13, 555-556.
PAML

Drosophila Genome Consortium 2007. Evolution of genes and genomes on the Drosophila phylogeny. *Nature*, 450, 203-218.
Genome wide comparative analysis of species differences

Pröschel, M., Zhang, Z. & Parsch, J. 2006. Widespread adaptive evolution of drosophila genes with sex-biased expression. *Genetics*, 174, 893-900.
Early study showing importance of sex-biased gene expression

Voight, B. F., Kudaravalli, S., Wen, X. Q. & Pritchard, J. K. 2006. A map of recent positive selection in the human genome. *Plos Biology*, 4, 446-458.
Loci under selection in humans?

References & case studies continued

Gardiner, A., Barker, D., Butlin, R. K., Jordan, W. C. & Ritchie, M. G. 2008. Drosophila chemoreceptor gene evolution: selection, specialization and genome size. *Molecular Ecology*, 17, 1648-1657.
Olfactory & gustatory loci in Drosophila, and ecological adaptation. Duplication important; neofunctionalisation.

Vieira, F., Sanchez-Gracia, A. & Rozas, J. 2007. Comparative genomic analysis of the odorant-binding protein family in 12 Drosophila genomes: purifying selection and birth-and-death evolution. *Genome Biology*, 8, R235.

Similar as Gardiner, but for odorant binding protein gene family
Feder, J. L., Egan, S. P. & Nosil, P. 2012. The genomics of speciation-with-gene-flow. *Trends in Genetics*, 28, 342-350.
Alternative approach; genome scans and islands of differentiation

Conclusions? For coding loci, purifying selection predominates, with some evidence of relaxed selection, e.g. on duplicates.

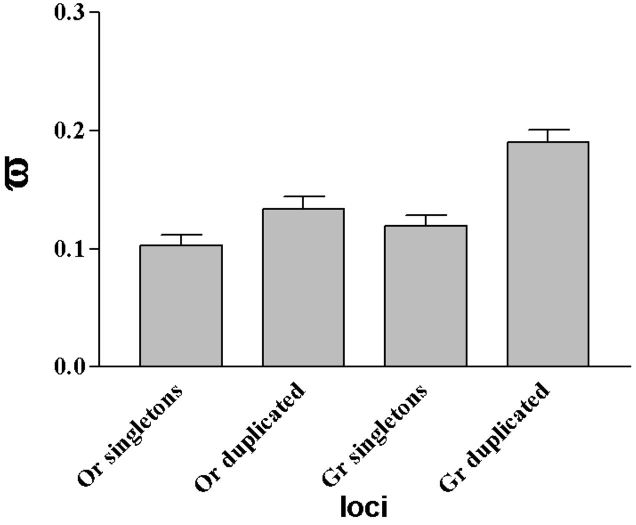

Gardiner *et al*. (2008).

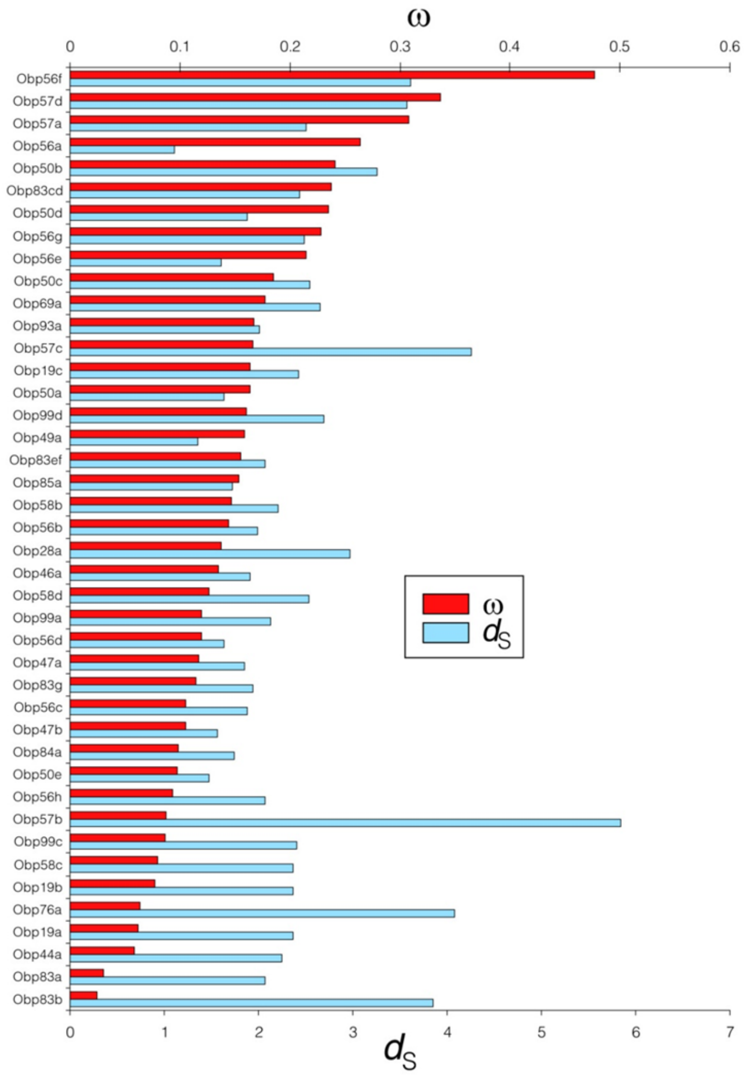

Vieira *et al*. (2007).

Why is ω rarely > 1?

- Becomes less reliable if d_S changes, e.g. at higher levels of divergence.
- Positive selection may be *rare* or very *transient*.
- Positive selection may be highly localised on regions of genes.
- Perhaps the more important changes are regulatory, not in coding regions?
- Combinations of bioinformatic studies and experimental manipulations much more powerful.
