# Supplementary material for: 4273π: Bioinformatics education on low cost ARM hardware
Source: BMC Bioinformatics. 2013 Aug 12;14:243. doi: 10.1186/1471-2105-14-243 (PMC3751261; doi:10.1186/1471-2105-14-243)
Supplement: Additional file 2 — 4273π Bioinformatics for Biologists teaching material, Version 1.01. The module handbook, lectures and practicals are included. The latest version, including Linux, software and BLAST databases, is available at the 4273π Web site [25]. [file 1471-2105-14-243-S2.zip › 4273pi_course_material/week8/practical_enzyme_func_evol.pdf]

4273π Bioinformatics for Biologists

Practical, Week 8: Enzyme function and evolution

John B.O. Mitchell, School of Chemistry, University of St Andrews.
Email jbom@st-andrews.ac.uk

© 2013 J.B.O. Mitchell. This is an Open Access document distributed under the terms of the Creative Commons Attribution License (http://creativecommons.org/licenses/by/2.0), which permits unrestricted use, distribution, and reproduction in any medium, provided the original work is properly cited.

4273π, Version 1.01. http://eggg.st-andrews.ac.uk/4273pi

1. Your task

Your task is to find out as much as you can about a given enzyme. Each student will be given a sequence, corresponding to a wild type, or possibly mutant, enzyme. You should use available online sources, including but not limited to a selection from those listed below, to gather data and knowledge. You should then produce a write-up approximately in the style of an online encyclopaedia article about the enzyme, and if relevant the specific sequence, highlighting the knowledge you've gained.

2. Types and sources of data

a) Sequence and Structure

For around 50 years, researchers have been describing enzymes with NC-IUBMB Enzyme Commission (EC) numbers, providing a simple (or perhaps simplistic) and hierarchical description of catalytic function. Over those five decades, sequencing, structural biology and bioinformatics have combined to revolutionise biochemical science, with millions of sequences deposited in UniProt and (as of autumn 2012) over 86,000 three dimensional structures in the Protein Data Bank (PDB). The major enzyme databases are well linked and hyperlinked to one another, so sequences in UniProt and X-ray or Nuclear Magnetic Resonance (NMR) structures from the PDB are quickly and easily available when browsing most other major databases. PDBsum provides excellent and visually rich summaries of the structures and interactions within the PDB, including the interactions in protein-ligand binding sites.

Uni-Prot (includes the carefully curated Swiss-Prot and the automatically annotated, unreviewed TrEMBL). http://www.uniprot.org/

Protein Data Bank "PDB" (3D X-ray & NMR structures). http://www.rcsb.org/pdb/home/home.do

PDBsum. http://www.ebi.ac.uk/pdbsum/

(b) Nomenclature, Classification and Metabolic Context

KEGG (Kyoto Encyclopaedia of Genes and Genomes) is an important resource, facilitating the understanding of enzymes in the metabolic pathway context. ExplorEnz is available for browsing and searching the full EC classification. It provides a supply of useful statistics; for example, there are currently (as of autumn 2012) 4867 extant EC numbers. The official NC-IUBMB website and IntEnz both act as portals to enzyme classification and nomenclature definitions and data.

KEGG: Kyoto Encyclopedia of Genes and Genomes. http://www.kegg.jp/

ExplorEnz-The Enzyme Database. http://www.enzyme-database.org/

IntEnz. http://www.ebi.ac.uk/intenz/

NC-IUBMB. http://www.chem.qmul.ac.uk/iubmb/enzyme/

(c) Catalytic Residues and General Information

BRENDA contains plentiful data on the chemistry, substrate specificity, kinetics, preparation and experimental sources of enzymes. Kinetic parameters may be particularly important in the coming years, since Bar-Even *et al.* have shown the rate acceleration and efficiency of real enzymes often to fall well below those of idealised, and arguably unrepresentative, case studies presented in many books and courses. The Catalytic Site Atlas (CSA) lists residues harbouring catalytic function within enzyme active sites, based on around a thousand original papers, with the related data inferred for roughly 27,000 homologues. The CSA has provided 3D templates which could be used to search a protein structure for spatial signatures of enzymatic activity, though that service is temporarily unavailable. These could be mined, for example, to find evidence for convergent evolution – two similar sets of reactive residues in evolutionarily unrelated sequences.

BRENDA, The Comprehensive Enzyme Information System. http://www.brenda-enzymes.info/

Catalytic Site Atlas (3D template searches currently not working). http://www.ebi.ac.uk/thornton-srv/databases/CSA/

(d) Chemical Reactions and their Mechanisms

Three databases describe, in somewhat different ways, the chemical mechanisms by which enzymes catalyse reactions. MACiE combines wide coverage of the available diversity of enzyme reactions with a full step-by-step description of each mechanism, up to the level of making, breaking, and changing the orders of covalent bonds. EzCatDB holds mechanisms for 828 enzyme-catalysed reactions (as of autumn 2012), these data being analysed within EzCatDB's unique RLCP classification system of mechanisms. The Structure-Function Linkage Database (SFLD) contains so-called "superfamilies", sets of divergently evolved enzymes usually of similar molecular function. The SFLD facilitates analysis of the evolutionary processes at three complementary levels: sequence, structure and chemical function.

EzCatDB. http://mbs.cbrc.jp/EzCatDB/

Structure−Function Linkage Database. http://sfld.rbvi.ucsf.edu/django/

MACiE. http://www.ebi.ac.uk/thornton-srv/databases/MACiE/

(e) Sequence Signatures

One of the most popular ways of predicting the function of a given sequence is to use sequence signatures. These are patterns, evolutionarily conserved and inferred from known protein sequences, which play the role of features in machine learning approaches to function prediction. Sequence signatures are often annotated with their functions as described in the original papers, protein structures, or mutants known for sequences encompassing the signatures. One of the major source databases for signatures is InterPro, an umbrella database formed from twelve other sources: CATH-Gene3D, HAMAP, PANTHER, Pfam, PIRSF, PRINTS, ProDom, PROSITE, SMART, SUPERFAMILY, and TIGRFAMs. From these signatures, InterPro curators select and refine the most reliable and useful sequence signatures. Such signatures can vary in length from short catalytic sites to entire protein domains of hundreds of residues, and in mutation tolerance from stringent to permissive. InterPro also hosts offline software and a browser-based web service (InterProScan) to match any given sequence against its library of sequence signatures.

InterPro. http://www.ebi.ac.uk/interpro/

(f) Phylogeny

The FunTree online application allows one to study the evolution of protein superfamilies, as defined using the CATH classification. FunTree uses structural alignments to generate so-called "structurally similar groups" (SSGs) as subsets of the superfamily, and to build phylogenetic trees that combine information from MACiE and the CSA. These allow structural superimpositions of enzymes at each node of the tree to be visualised.

FunTree. http://www.ebi.ac.uk/thornton-srv/databases/FunTree/

3. Sequences from UniProt

Students will be randomly assigned one of these:

P51178,
P49053,
P60527,
Q8KFN8,
B0SXA8,
P52700,
Q2IJC6.
