# Supplementary material for: 4273π: Bioinformatics education on low cost ARM hardware
Source: BMC Bioinformatics. 2013 Aug 12;14:243. doi: 10.1186/1471-2105-14-243 (PMC3751261; doi:10.1186/1471-2105-14-243)
Supplement: Additional file 2 — 4273π Bioinformatics for Biologists teaching material, Version 1.01. The module handbook, lectures and practicals are included. The latest version, including Linux, software and BLAST databases, is available at the 4273π Web site [25]. [file 1471-2105-14-243-S2.zip › 4273pi_course_material/week8/lecture_enzyme_func_evol.pdf]

Enzyme Function & Evolution

4273π Bioinformatics for Biologists

Lecture, Week 8

John B.O. Mitchell, School of Chemistry, University of St Andrews

Email jbom@st-andrews.ac.uk

© 2013 J.B.O. Mitchell. This is an Open Access document distributed under the terms of the Creative Commons Attribution License (http://creativecommons.org/licenses/by/2.0), which permits unrestricted use, distribution, and reproduction in any medium, provided the original work is properly cited.

4273π, Version 1.01. http://eggg.st-andrews.ac.uk/4273pi

Part 1: Theories of Enzyme Evolution

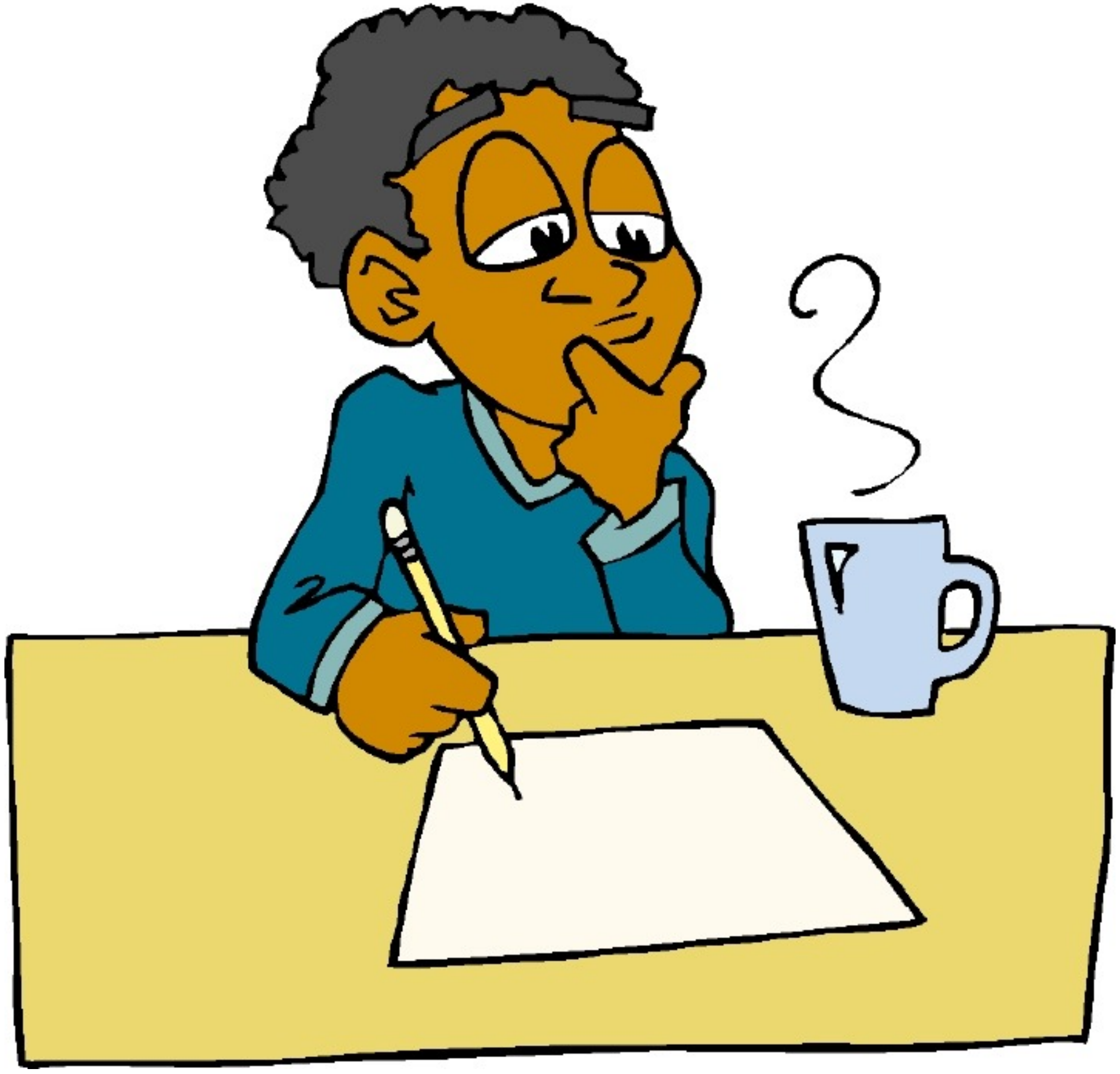

Overview

(1) Divergent retrograde evolution, recruiting adjacent enzymes in pathway and constrained by binding similar molecules as substrates or products.

Overview

(2) Divergent patchwork evolution, recruiting enzymes catalysing similar chemical reactions, typically from other pathways, constrained by supporting similar catalytic chemistry.

Overview

(3) Convergent evolution, reinventing similar chemistry in a different evolutionary family.

Theory of Retrograde Evolution (Horowitz,1945)

Pathways evolve backwards: the end product of the newly evolved reaction is the substrate of the existing one.

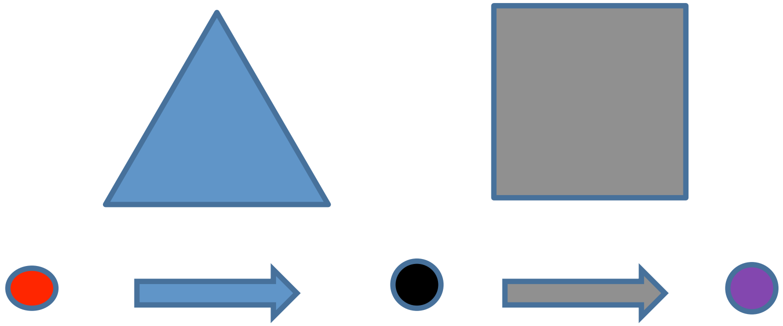

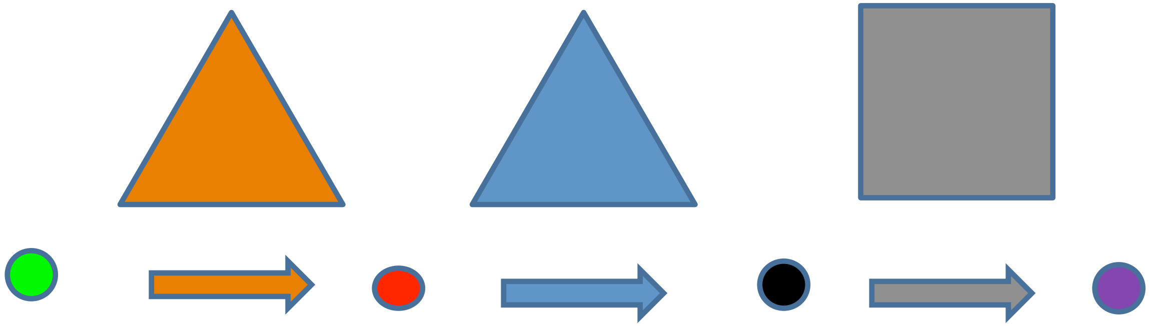

Retrograde
duplication

Successive reactions in the pathway would therefore be catalysed by homologous enzymes

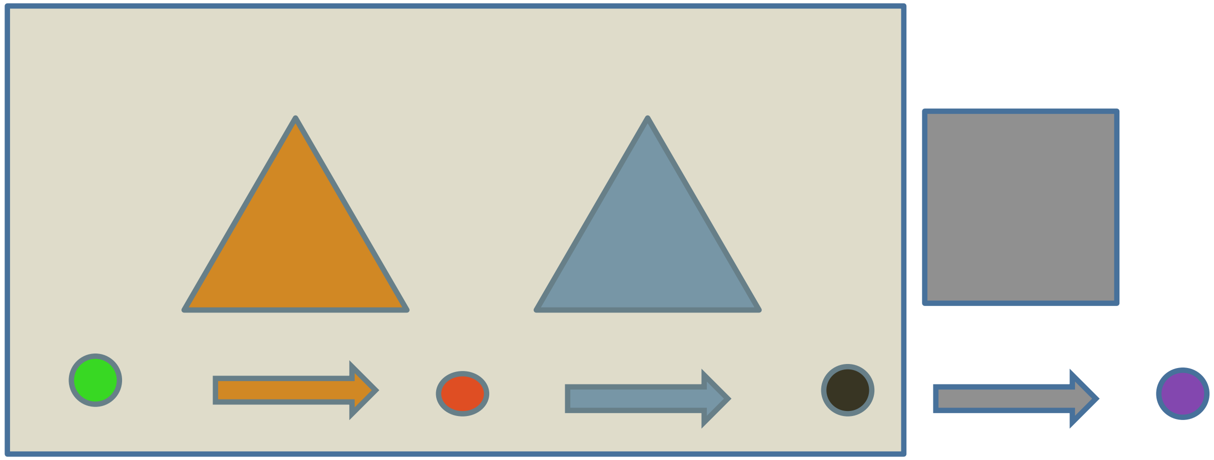

Retrograde duplication

Theory of Patchwork Evolution (Jensen,1976)

Recruitment of enzymes for new reactions was based on similarity of reactions catalysed and possibly on substrate ambiguity.

It did not necessarily require the sequential and backwardly evolving progression of steps.

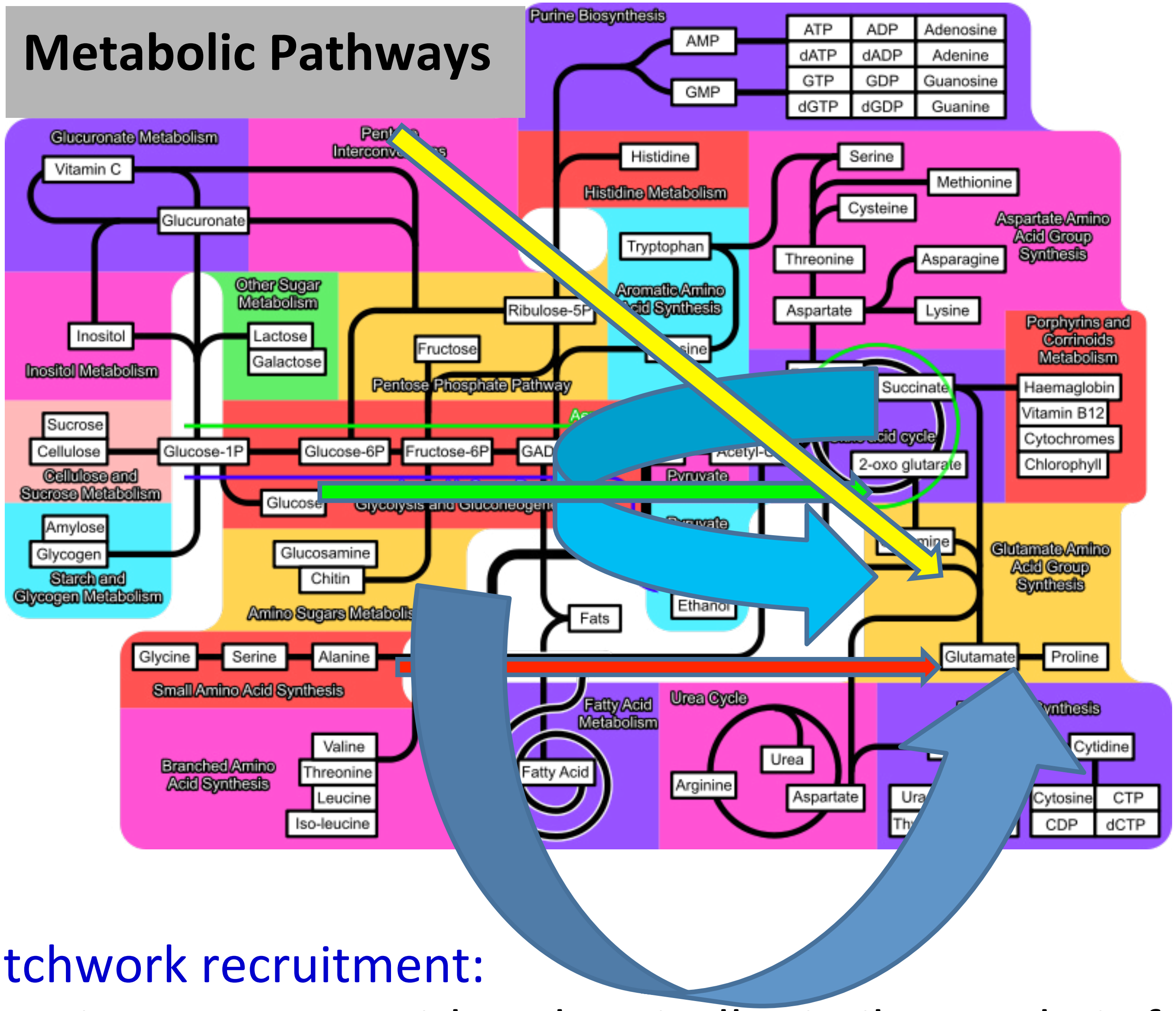

Patchwork recruitment:

Recruit an enzyme with a chemically similar catalytic function from a quite different pathway.

Image credit: http://commons.wikimedia.org/wiki/File:Metabolism_790px.svg

Retrograde evolution:

Recruit metabolically adjacent enzyme.

Patchwork evolution:

Recruit metabolically distant but catalytically similar enzyme.

The Importance of Moonlighting

A moonlighting enzyme has a second job.

The Importance of Moonlighting

Patchwork recruitment is most likely to occur when the original enzyme already has some low level of activity for catalysing a different reaction.

The Importance of Moonlighting

This allows the enzyme to be recruited to carry out the new function.

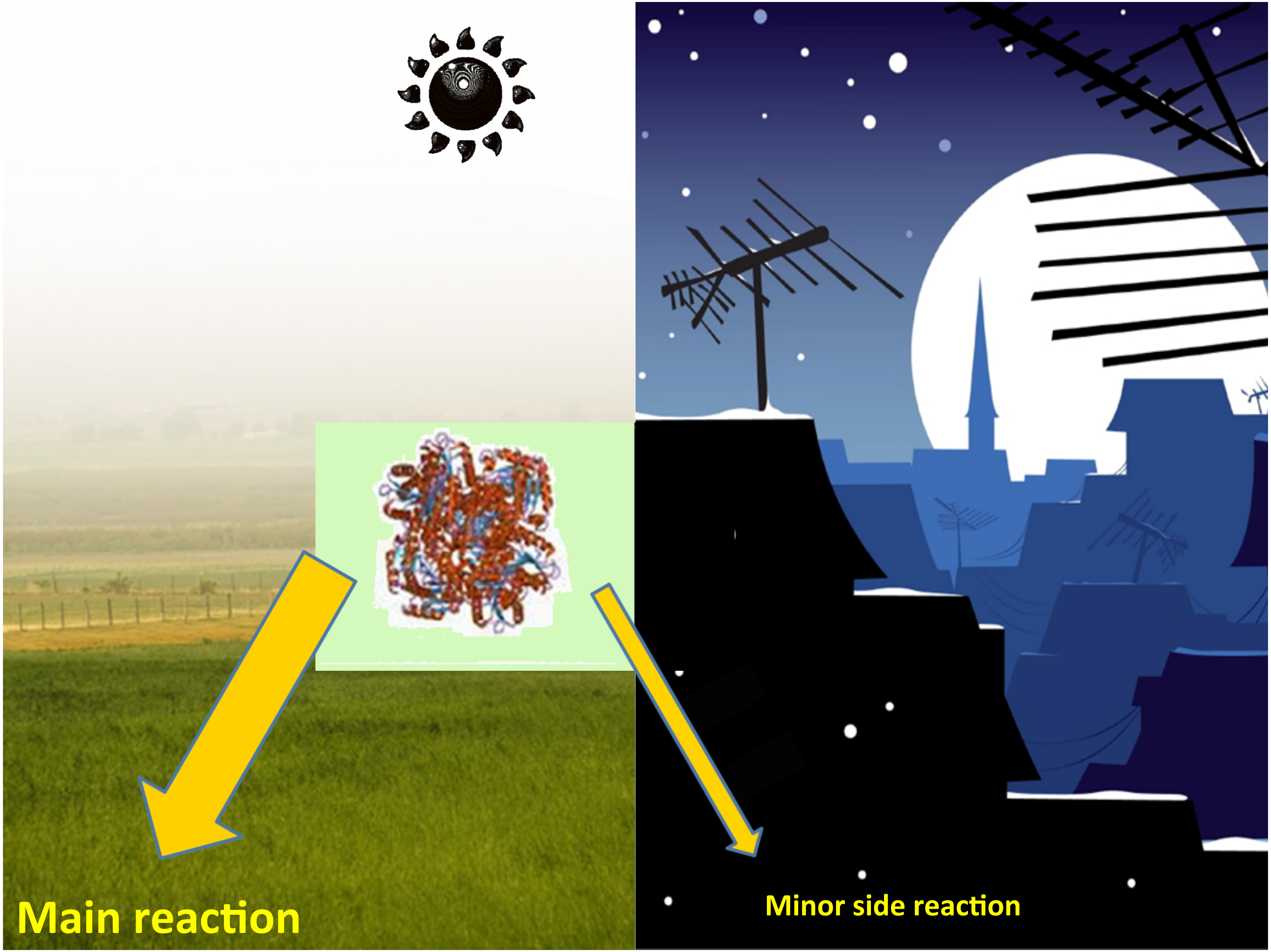
Main reaction
Minor side reaction

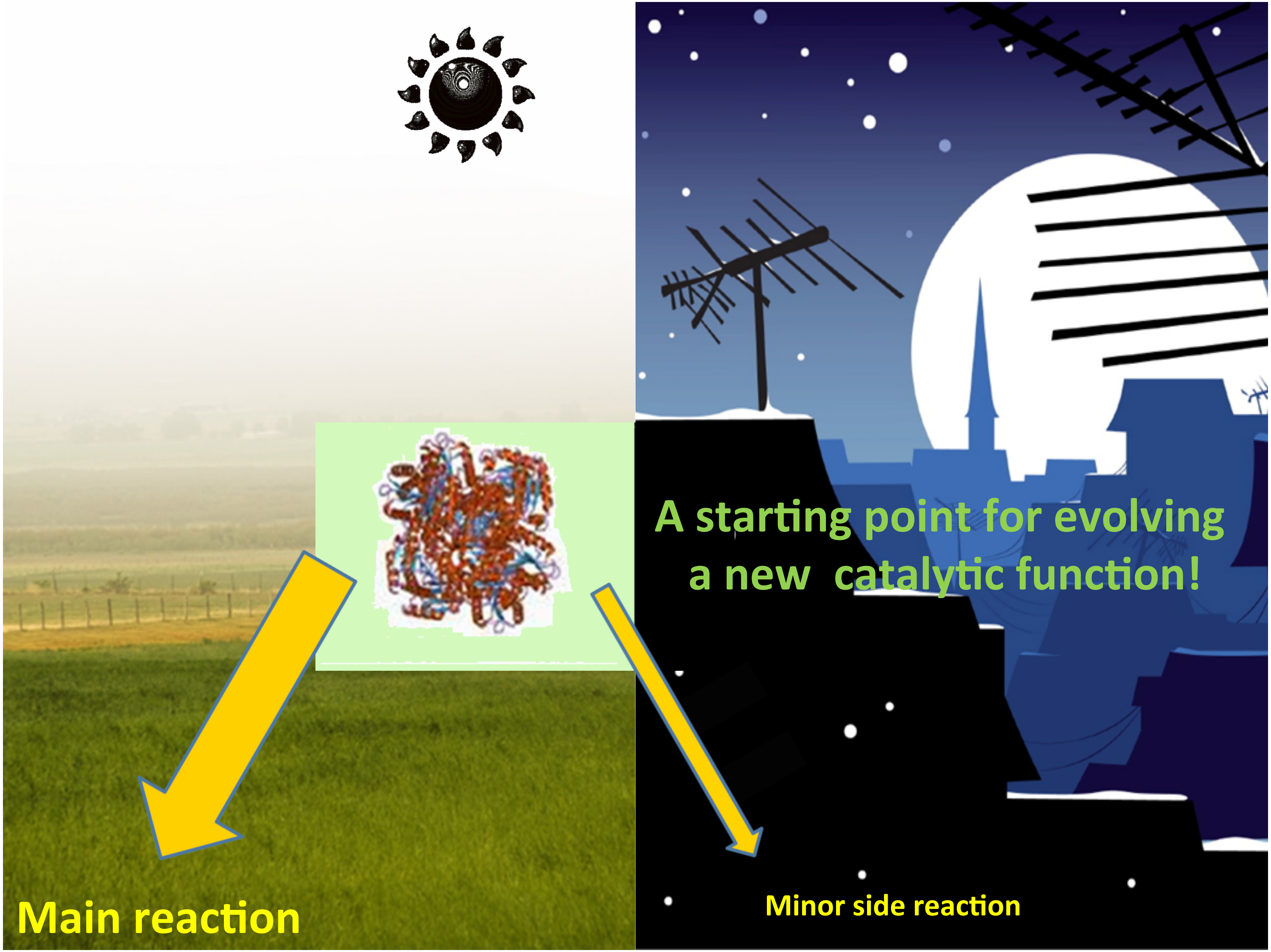
A starting point for evolving a new catalytic function!
Main reaction
Minor side reaction

How Might Divergent Evolution Occur?

At the level of the gene, the most obvious idea is via gene duplication with one copy being free to mutate away from its original function

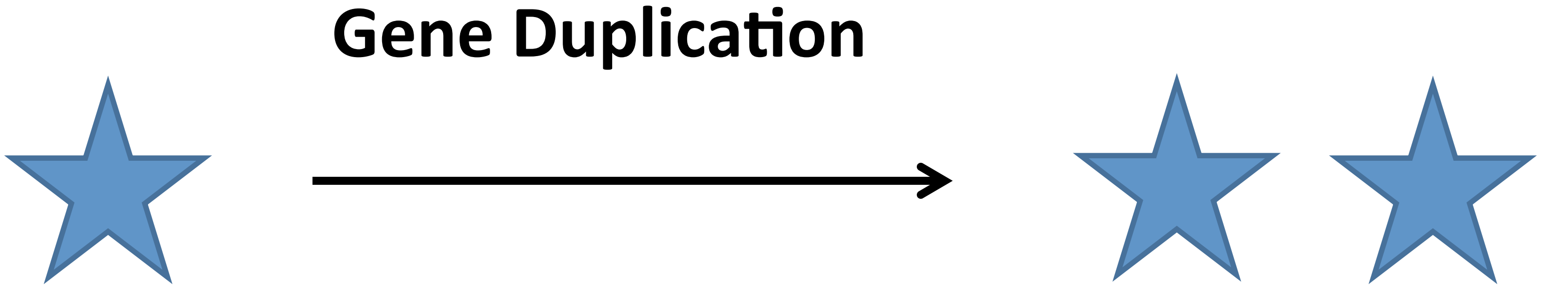
Gene Duplication

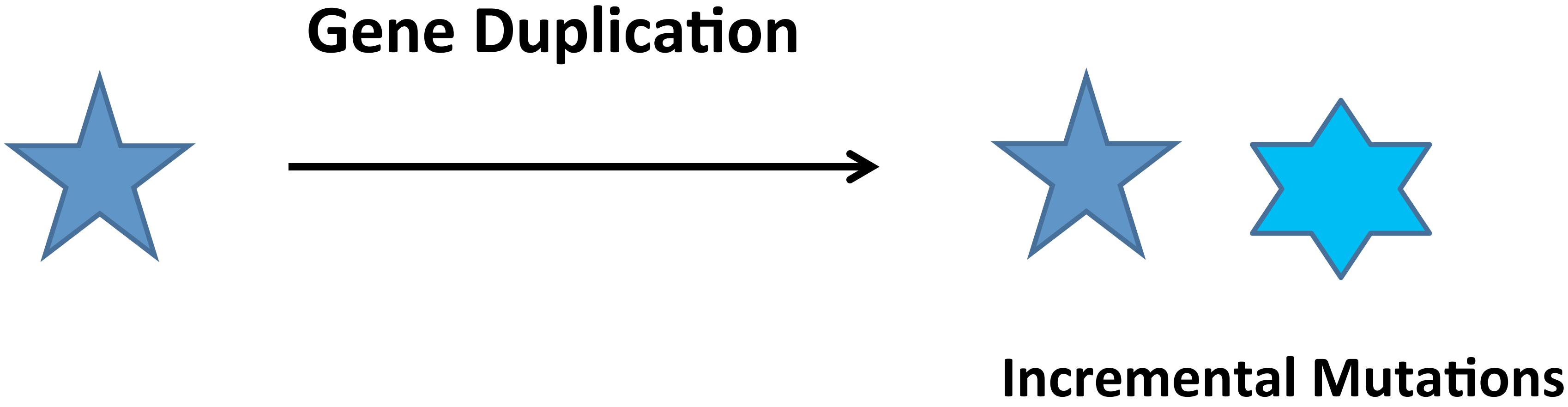
Gene Duplication
Incremental Mutations

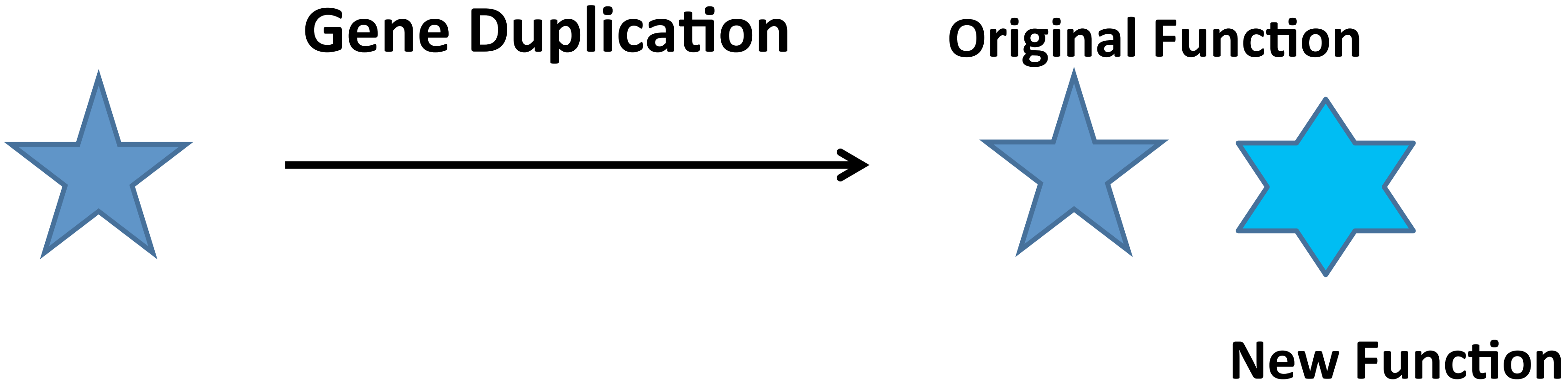
Gene Duplication
Original Function
New Function

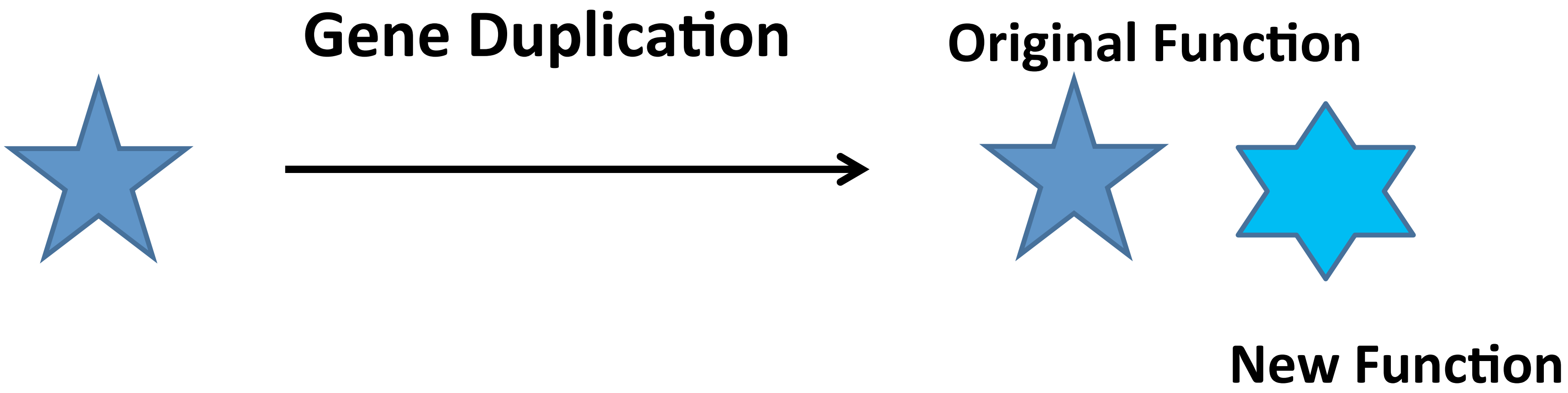

This way, the original function is maintained ***and*** a new one evolved.

How Might Divergent Evolution Occur?

There are other possible routes to diverged functions (Orengo, Thornton, Todd & others), several may occur!

Todd, Orengo & Thornton, *Current Opinion in Chemical Biology* **3**:548–556 (1999)

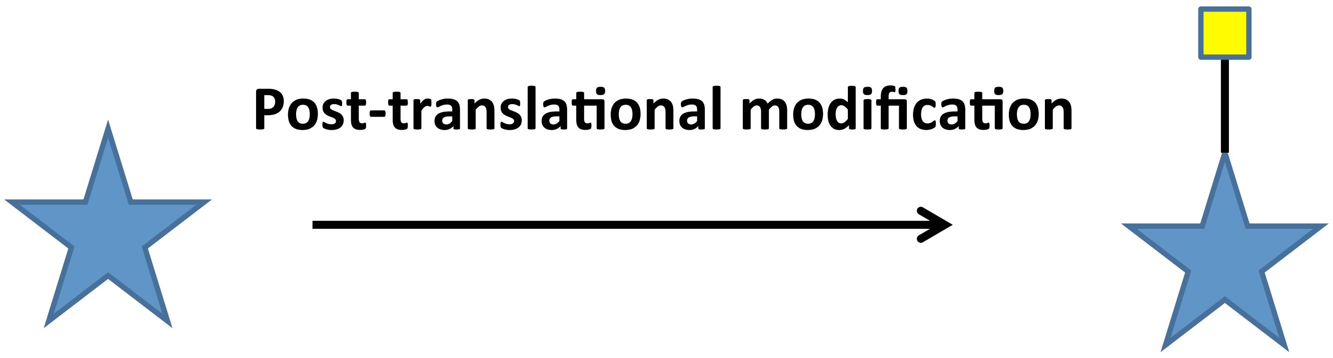
Post-translational modification

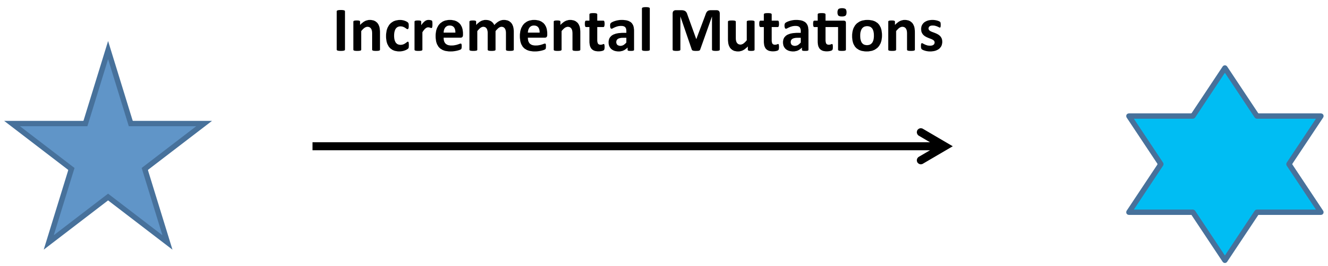
Incremental Mutations

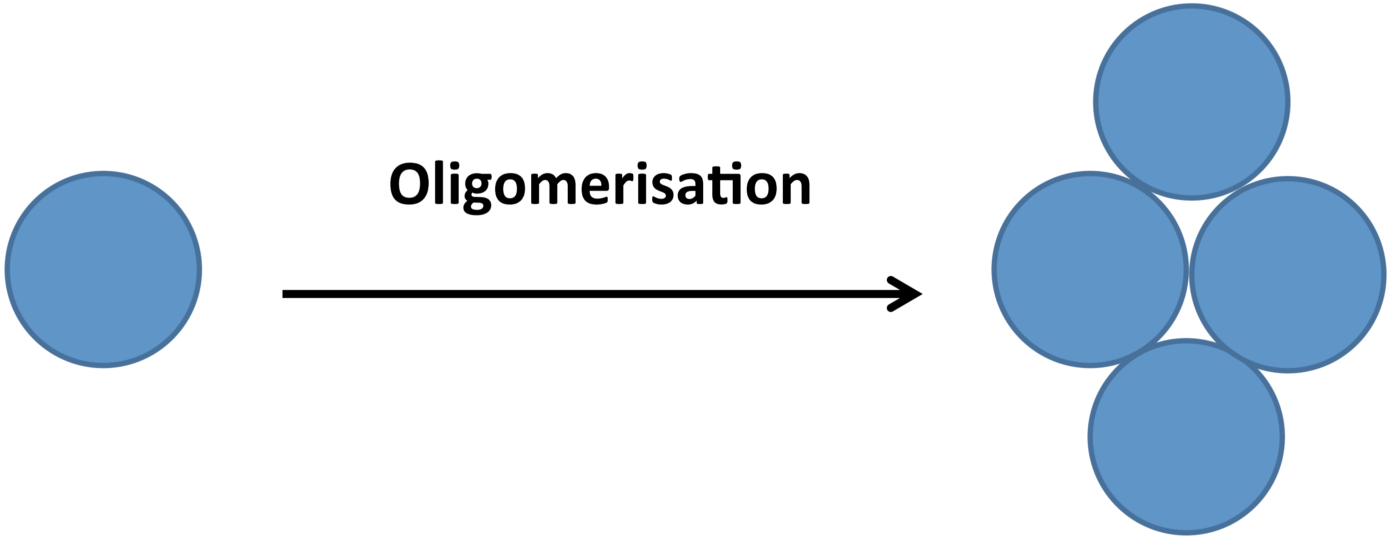
Oligomerisation

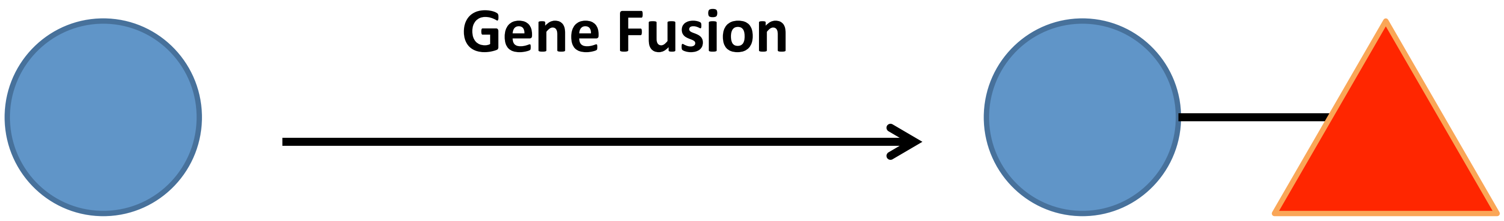
Gene Fusion

Models for Divergent Enzyme Evolution

Two main models of divergent enzyme evolution discussed by Gerlt and Babbitt:

Chemistry is conserved, substrate specificity changes.

Substrate binding is conserved, chemistry changes.

Evidence for both models in different cases, but conserved chemistry is likely to be more common.

Gerlt and Babbitt, *Annu. Rev. Biochem.* **70**:209–246 (2001)

Models for Divergent Enzyme Evolution

Two main models of divergent enzyme evolution discussed by Gerlt and Babbitt:

Chemistry is conserved, substrate specificity changes.

If true, implies that chemical reactions are harder to evolve than is substrate binding.

Fits well with patchwork recruitment model.

Phylogeny of Enzymes (Caetano-Annolés)

Taking advantage of the genomic data now available, Caetano-Annolés and group attempted to build a phylogeny of enzymes based on the occurrence of their folds in sequenced genomes.

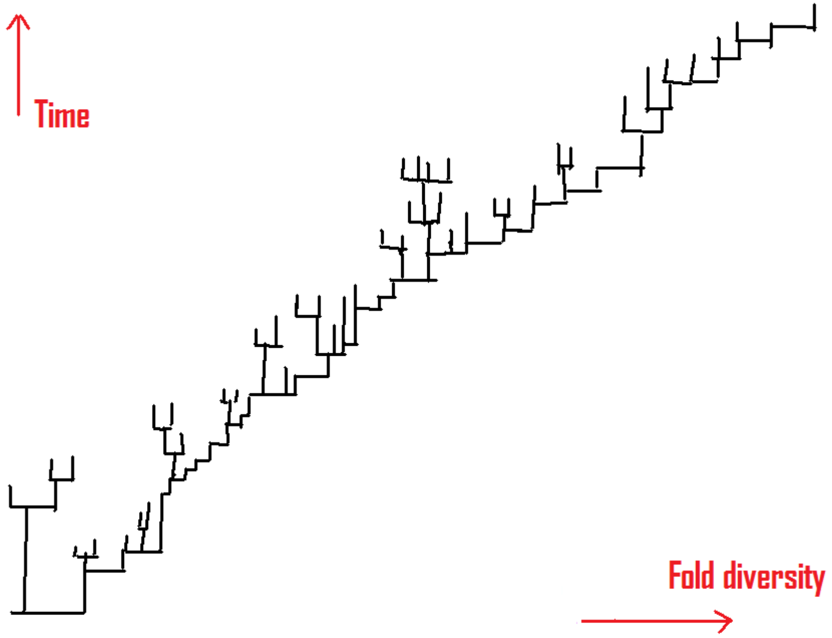

Caetano-Annolés *et al.*, *Proc. Natl Acad. Sci. USA* **104**:9358 (2007)

Phylogeny of Enzymes (Caetano-Annolés)

In principle, this could "age" enzymes
– the more universal the older.

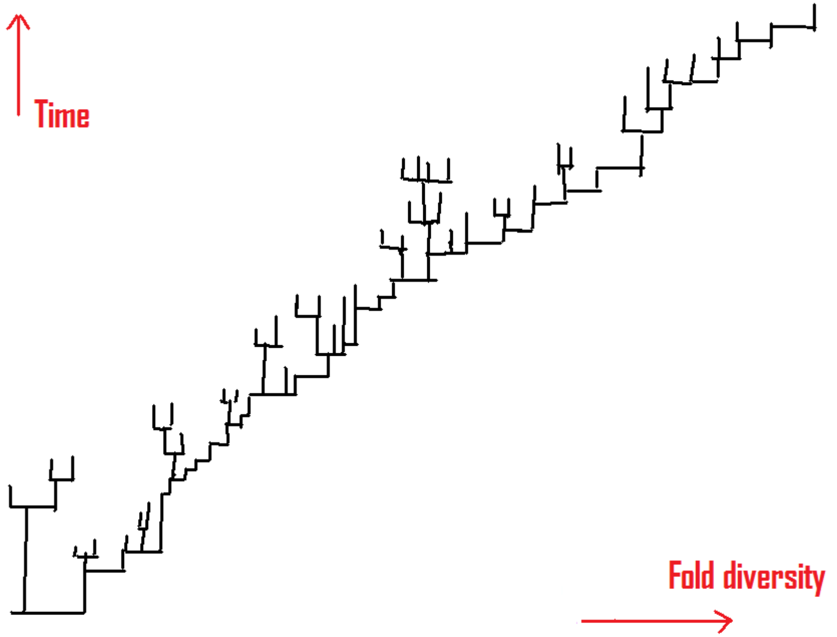

# Both Divergent & Convergent Evolution are Important

Divergent evolution leads to one fold performing a plurality of functions.

Convergent evolution leads to a plurality of folds performing the same function

Divergent Evolution

Many evolutionary superfamilies have acquired the ability to catalyse more than one kind of chemical reaction (third level EC number).

Divergent Evolution: Reactions per Superfamily

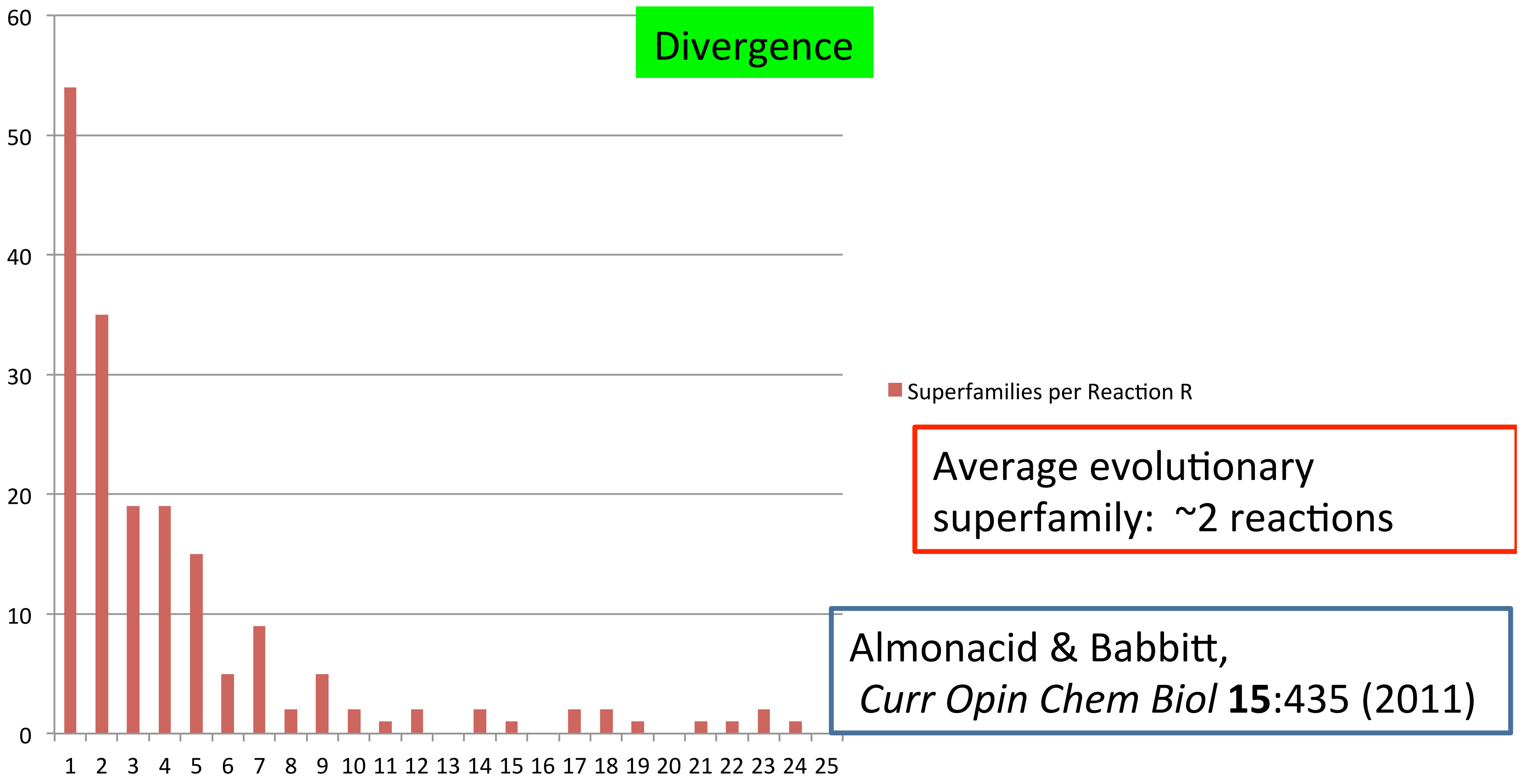

Average evolutionary superfamily: ~2 reactions

Almonacid & Babbitt, *Curr Opin Chem Biol* **15**:435 (2011)

Convergent Evolution

Catalysis of most chemical reactions (third level EC numbers) has evolved convergently in different enzyme superfamilies.

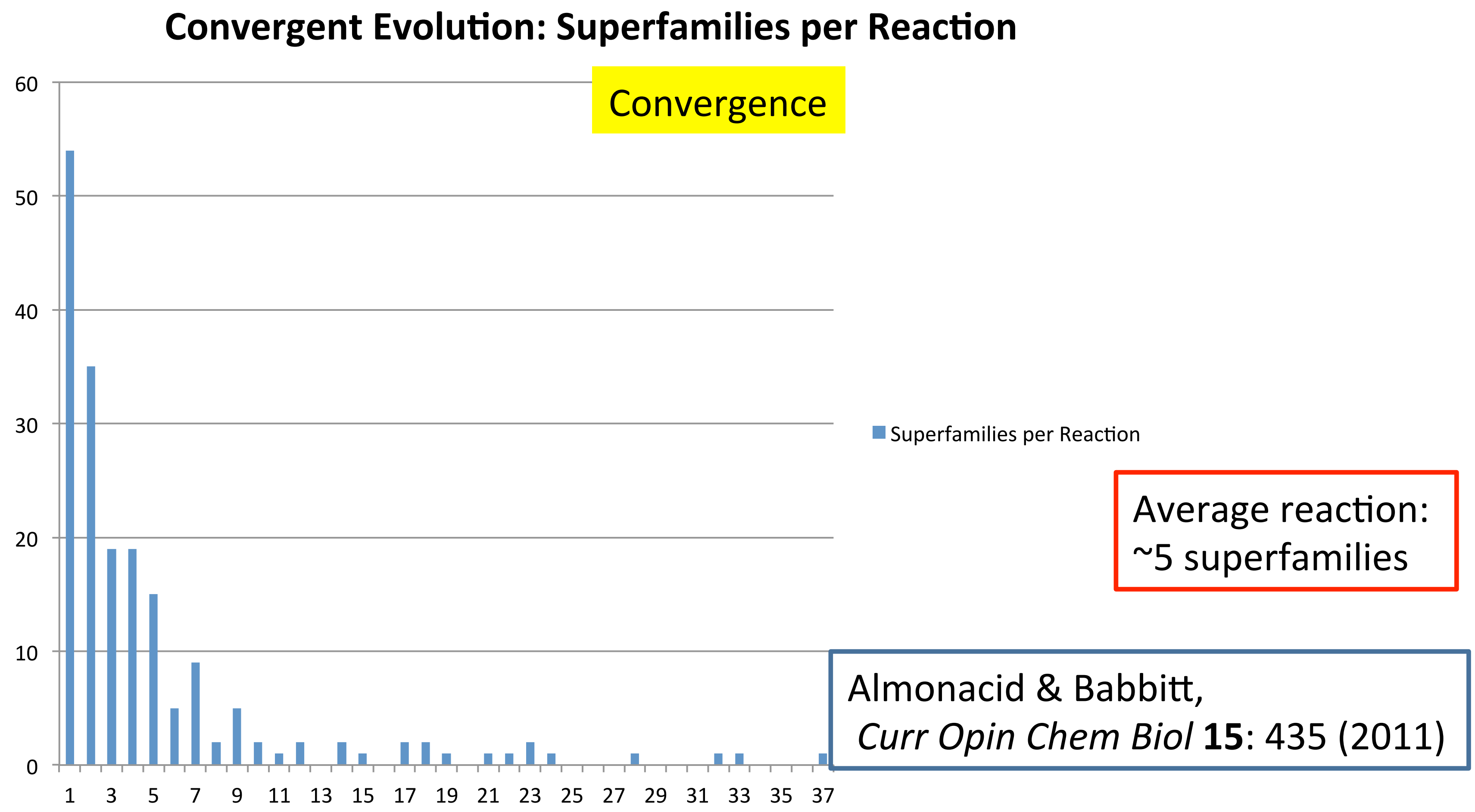

Average reaction:
~5 superfamilies

Almonacid & Babbitt,
Curr Opin Chem Biol **15**: 435 (2011)

Those figures are based on available structures. As more become available, we will find more functions for existing folds, and more folds with existing functions.

So these are underestimates!

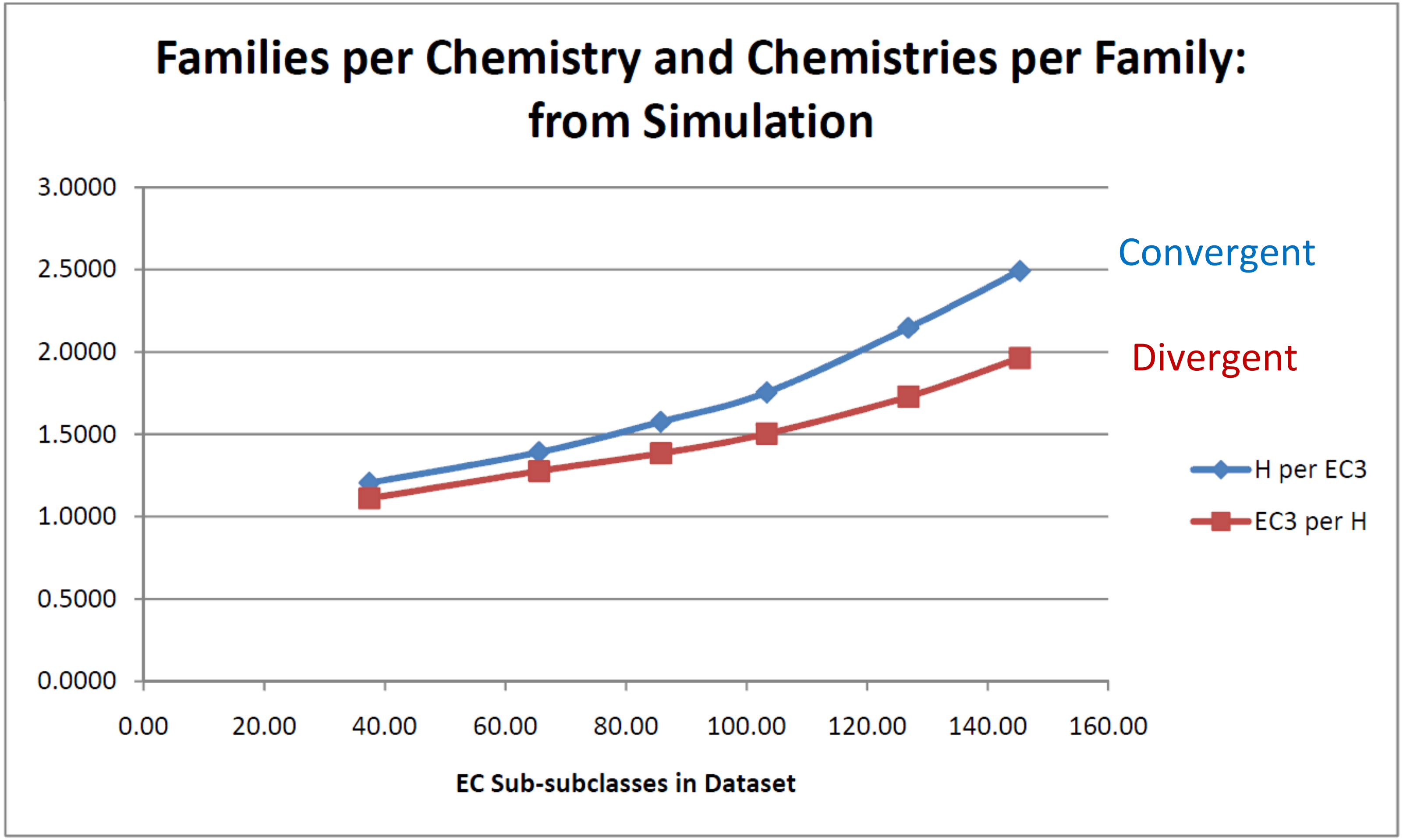

Caveat

Our working definition of "Convergent Evolution" is dependent on the EC classification, which is not a perfect gold standard.

Summary of Evidence for Evolutionary Theories

- Both convergent and divergent evolution are important.
- Patchwork evolution is quite common.
- Retrograde evolution does sometimes occur (adjacent reactions in pathways have similar enzymes more often than by chance), but is not strongly prevalent.
- Consistent with the above, conservation of chemistry is more important than conservation of substrate binding.

Part 2: Studying Evolution and Function of Enzymes with Bioinformatics

- Some specific examples of bioinformatics approaches to scientific problems.

Reconstructing Enzyme Phylogenies

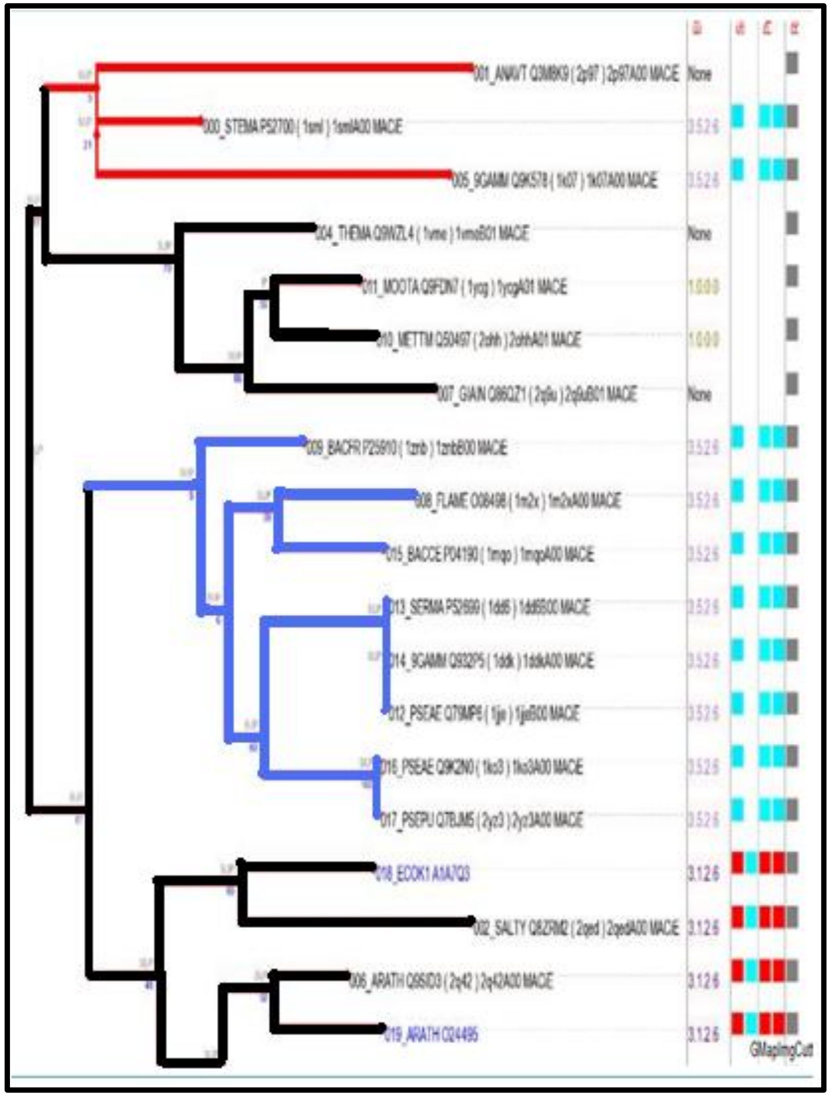

http://www.ebi.ac.uk/thornton-srv/databases/FunTree/

Evolutionary origins of the B1 & B3 metallo- beta-lactamases

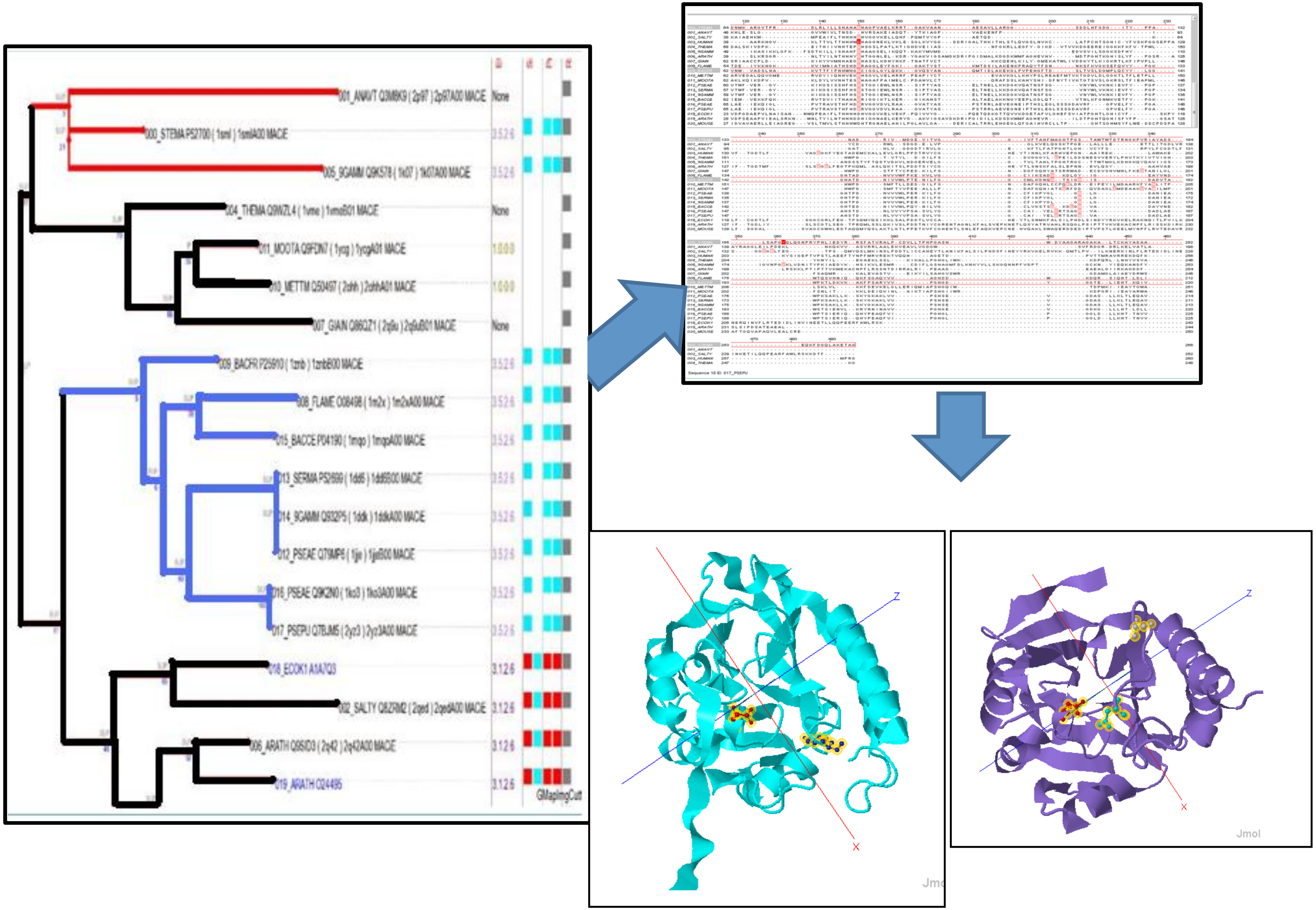

Was beta-lactamase activity ancestral and lost in two branches?

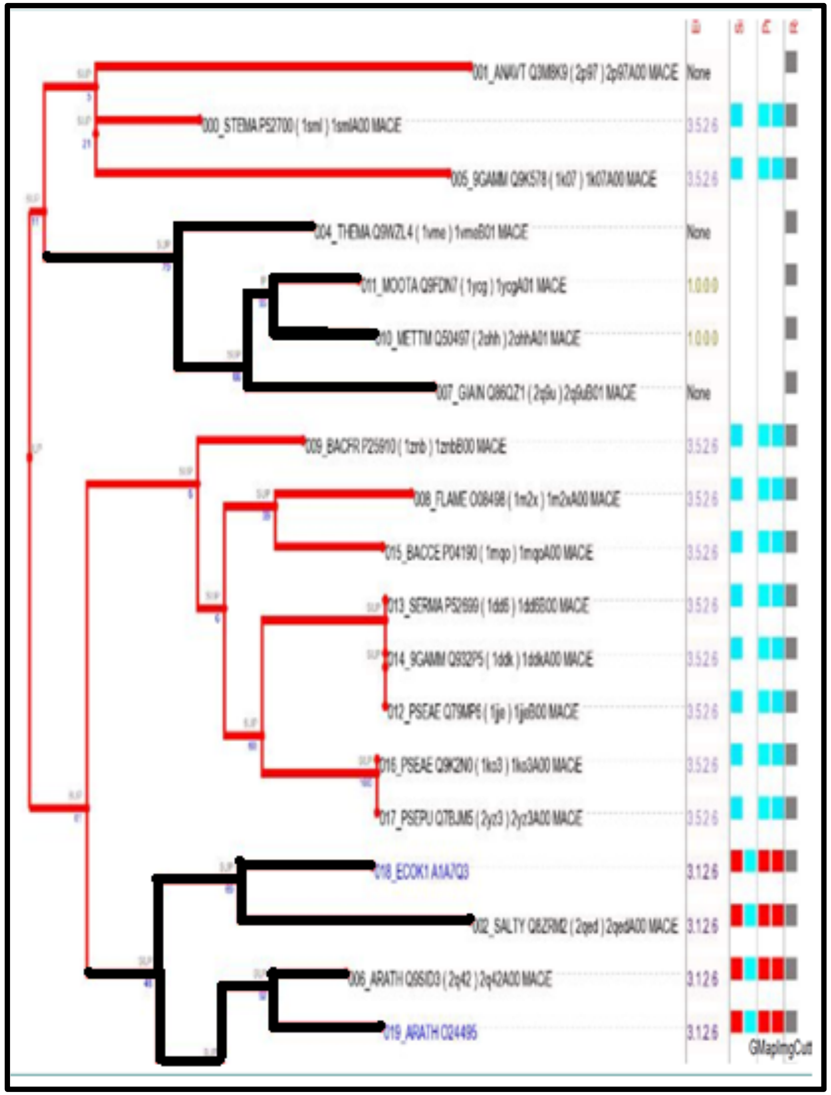

Or did beta-lactamase activity evolve independently twice?

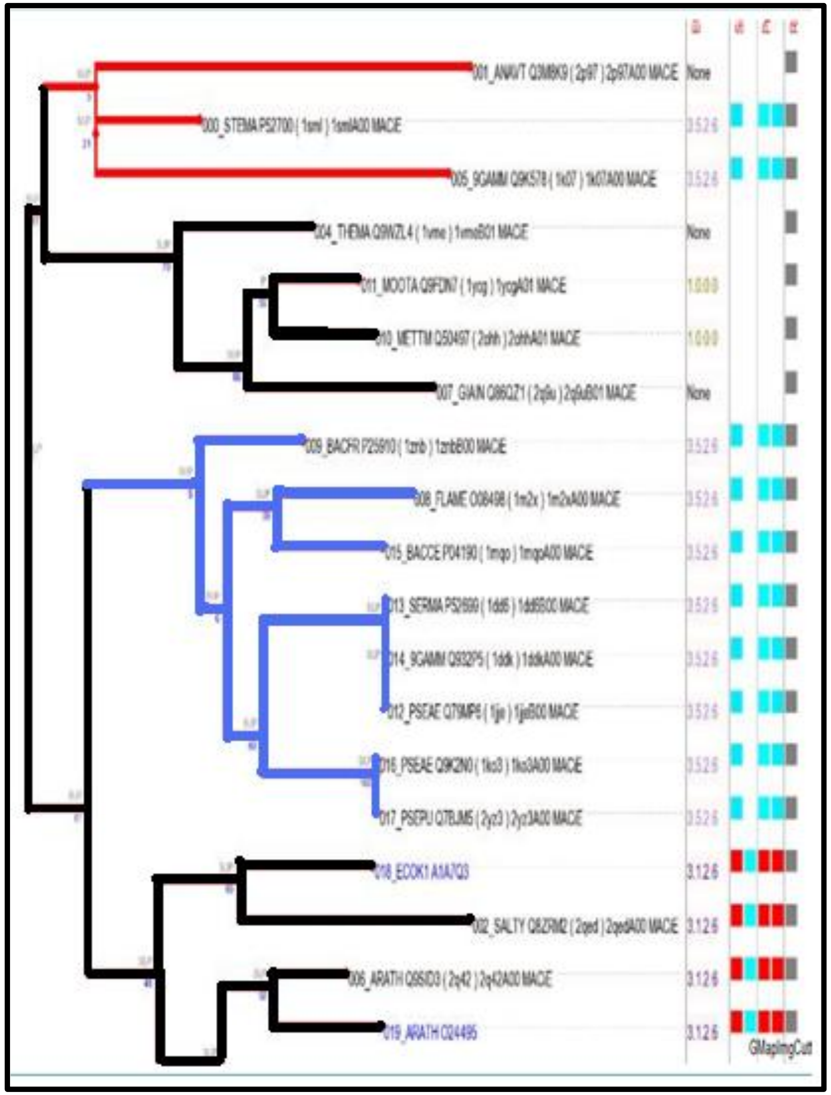

Reconstructing Evolutionary History

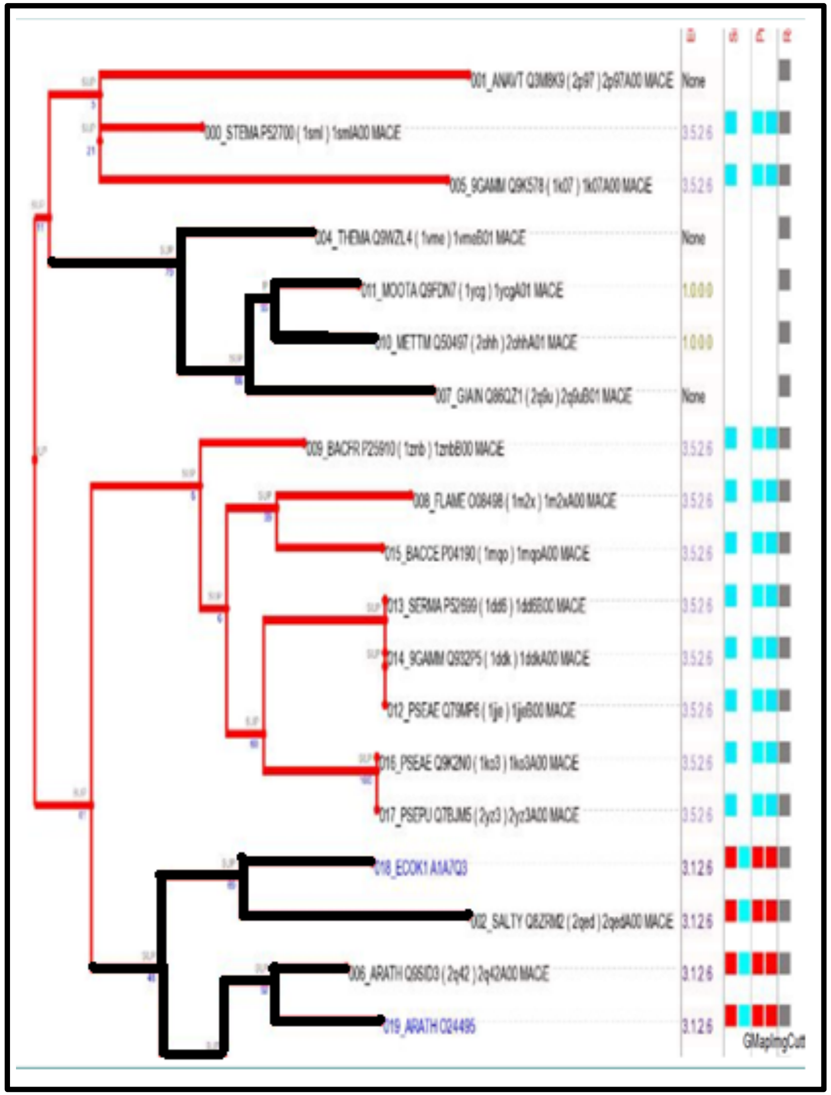

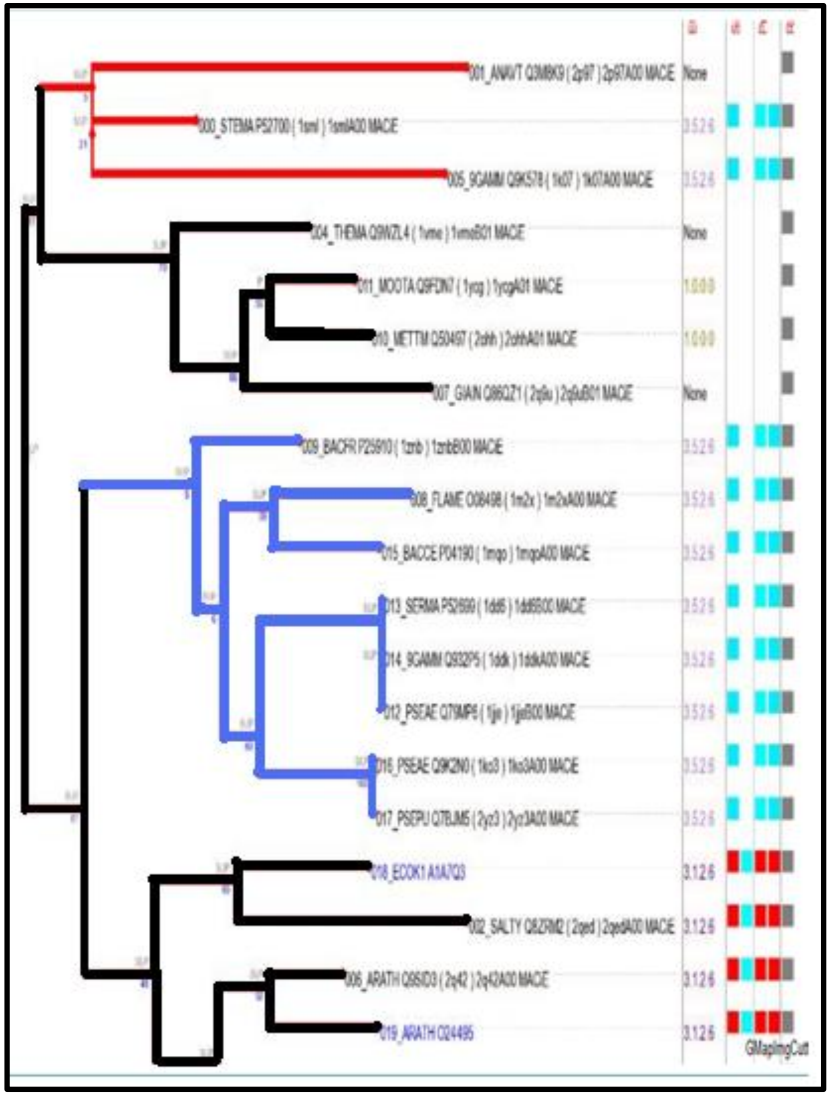

Compare the likelihoods probabilistically by constructing many different possible histories. Find the most likely tree topology.

MACiE Database

Mechanism, Annotation and Classification in Enzymes

http://www.ebi.ac.uk/thornton-srv/databases/MACiE/

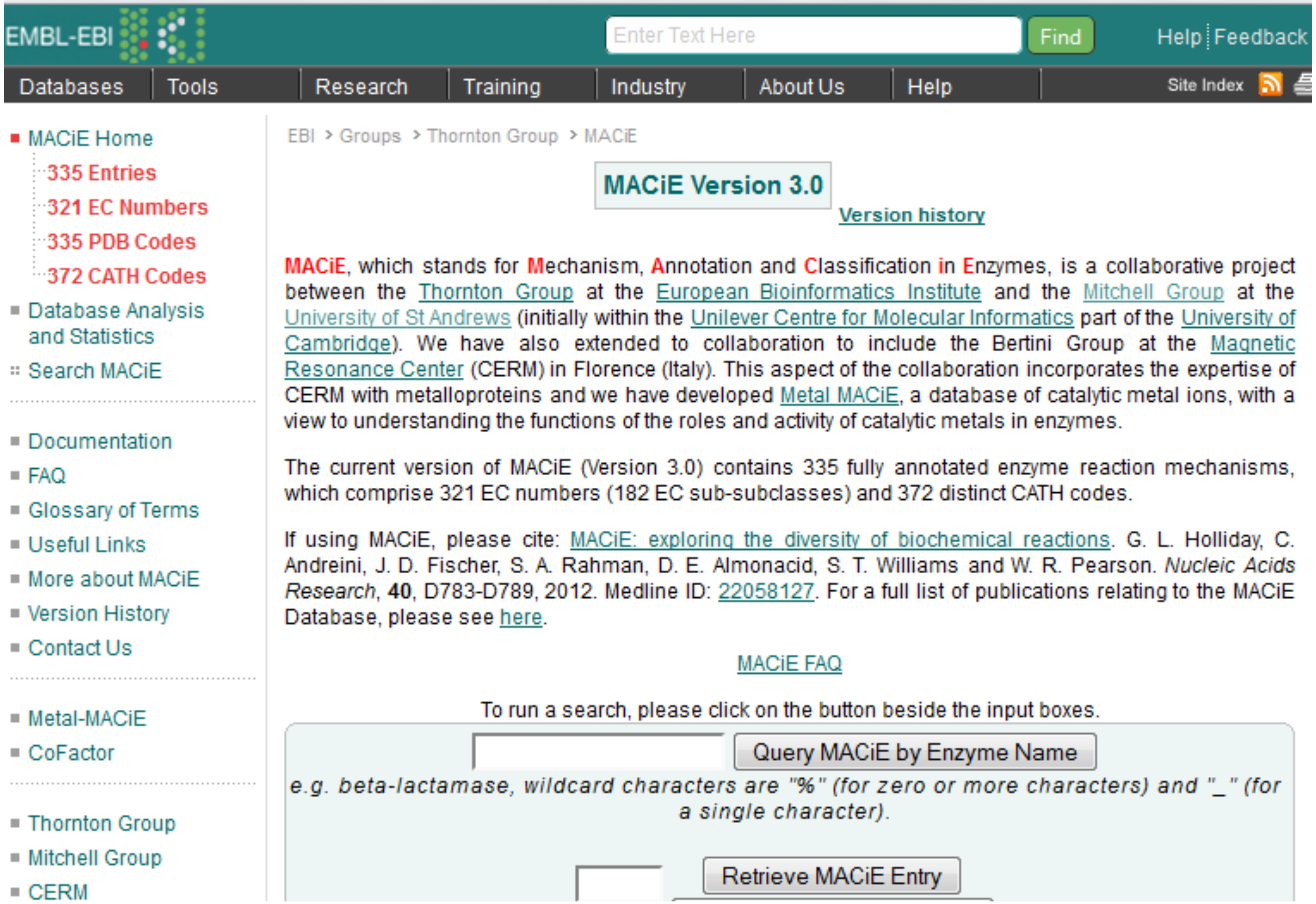

EMBL-EBI | Enter Text Here | Find | Help | Feedback

Databases | Tools | Research | Training | Industry | About Us | Help | Site Index

- MACiE Home
  - 335 Entries
  - 321 EC Numbers
  - 335 PDB Codes
  - 372 CATH Codes
- Database Analysis and Statistics
- Search MACiE

- Documentation
- FAQ
- Glossary of Terms
- Useful Links
- More about MACiE
- Version History
- Contact Us

- Metal-MACiE
- CoFactor

- Thornton Group
- Mitchell Group
- CERM

EBI > Groups > Thornton Group > MACiE

MACiE Version 3.0

Version history

MACiE, which stands for Mechanism, Annotation and Classification in Enzymes, is a collaborative project between the Thornton Group at the European Bioinformatics Institute and the Mitchell Group at the University of St Andrews (initially within the Unilever Centre for Molecular Informatics part of the University of Cambridge). We have also extended to collaboration to include the Bertini Group at the Magnetic Resonance Center (CERM) in Florence (Italy). This aspect of the collaboration incorporates the expertise of CERM with metalloproteins and we have developed Metal MACiE, a database of catalytic metal ions, with a view to understanding the functions of the roles and activity of catalytic metals in enzymes.

The current version of MACiE (Version 3.0) contains 335 fully annotated enzyme reaction mechanisms, which comprise 321 EC numbers (182 EC sub-subclasses) and 372 distinct CATH codes.

If using MACiE, please cite: MACiE: exploring the diversity of biochemical reactions. G. L. Holliday, C. Andreini, J. D. Fischer, S. A. Rahman, D. E. Almonacid, S. T. Williams and W. R. Pearson. *Nucleic Acids Research*, **40**, D783-D789, 2012. Medline ID: 22058127. For a full list of publications relating to the MACiE Database, please see here.

MACiE FAQ

To run a search, please click on the button beside the input boxes.

Query MACiE by Enzyme Name

e.g. beta-lactamase, wildcard characters are "%" (for zero or more characters) and "_" (for a single character).

Retrieve MACiE Entry

Enzyme Nomenclature and Classification

EC Classification

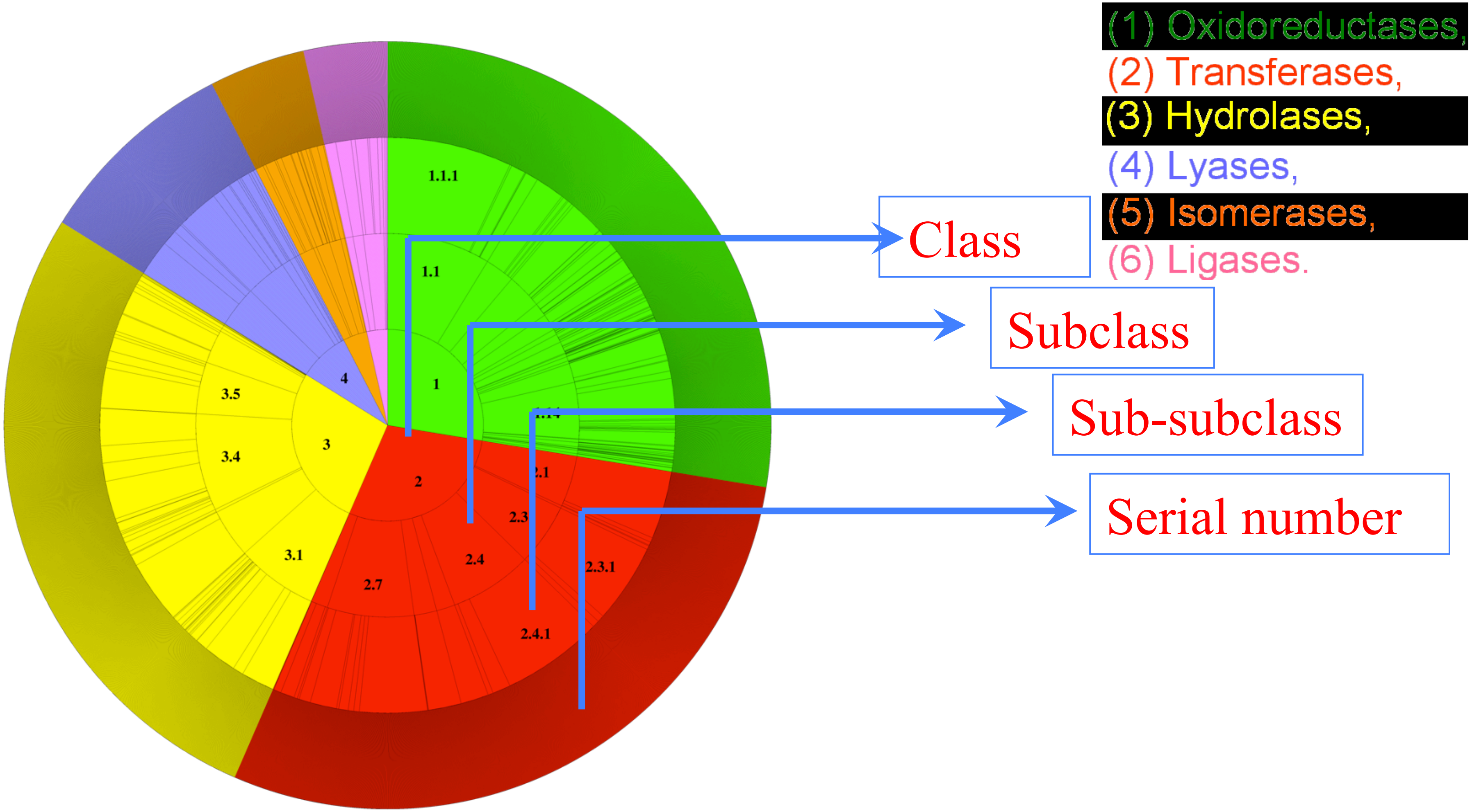

The EC Classification

- Deals with overall reaction, not mechanism
- Reaction direction arbitrary
- Cofactors and active site residues ignored
- Doesn’t deal with structural and sequence information
- However, it was never intended to do so

A New Representation of Enzyme Reactions?

- Should be complementary to, but distinct from, the EC system
- Should take into account:
  - Reaction Mechanism
    - Structure
      - Sequence
      - Active Site residues
      - Cofactors

- Need a database of enzyme mechanisms

- MACiE Home
  - 335 Entries
  - 321 EC Numbers
  - 335 PDB Codes
  - 372 CATH Codes
- Database Analysis and Statistics
- Search MACiE

Overview Enzyme Information for M0001

glutamate racemase
EC: 5.1.1.3
PDB: 1b73

This enzyme has the following catalytic CATH domains:

3.40.50.1860
Unassigned Domain

This enzyme has the following catalytic UNIPROT codes:

P56868

- Overview
  - Structural Overview
  - Similar Reactions Overall
  - Similar Reactions Composite
- Animated Reaction
- Reaction Steps
  - Step 01
  - Step 02
  - Step 03
  - Step 04
- Documentation
- FAQ
- Glossary of Terms
- Useful Links
- More about MACiE

Overview for MACiE Entry M0001

Version history

General Information

EC Number: 5.1.1.3 (*A member of the Isomerases, Racemases and epimerases, Acting on amino acids and derivatives*)

Enzyme Name: glutamate racemase

Biological Species: *Aquifex pyrophilus* (Bacteria)

Catalytic Chain UniprotKB Accession Codes:

- P56868 - *Glutamate racemase*

Representative PDB Code: 1b73 - *GLUTAMATE RACEMASE FROM AQUIFEX PYROPHILUS* (Resolution = 2.30 Å).

Catalytic CATH Codes:

- 3.40.50.1860 - *Rossmann fold*
- Unassigned Domain

Display structure information

Overall Reaction:

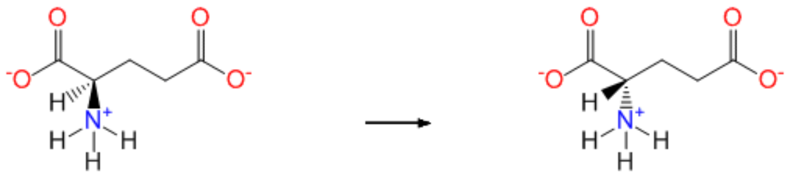

L-glutamate
C00025
CHEBI:29985

D-glutamate
C00217
CHEBI:29986

Overall Comment: The mechanism shown in the following steps refers to the direction shown in overall reaction not in the reverse direction. Either Glu147' or Asp7 can deprotonate the appropriate cysteine residue.

View similar reactions

Stepwise Description of the Reaction

Step 1	Asp7 deprotonates Cys70, activating it.
Step 2	Cys70 deprotonates the L-glutamate substrate, initiating a double bond rearrangement, resulting in the planar enol.
Step 3	The oxyanion initiates a further double bond rearrangement, which results in the deprotonation of Cys178, which is on the opposite side of Cys70, resulting in the formation of the opposite isomer.

- MACiE Home
  - Entries
  - EC Numbers
  - PDB Codes
  - CATH Codes
- Database Statistics
- Search MACiE

Overview Enzyme Information for M0001

glutamate racemase
EC: 5.1.1.3
PDB: 1b73

This enzyme has the following catalytic CATH domains:
3.40.50.1860

This enzyme has the following catalytic UNIPROT codes:
P56868

- Overview
- Animated Reaction
- Reaction Steps
  - Step 01
  - Step 02
  - Step 03
  - Step 04
- Documentation
- FAQ
- Glossary of Terms
- Useful Links
- More about MACiE
- Version History
- Contact Us
- Metal-MACiE
- Thornton Group
- Mitchell Group
- CERM

EBI > Groups > Thornton Group > MACiE > M0001

Entry M0001 5.1.1.3 glutamate racemase

Next Step

Step 01

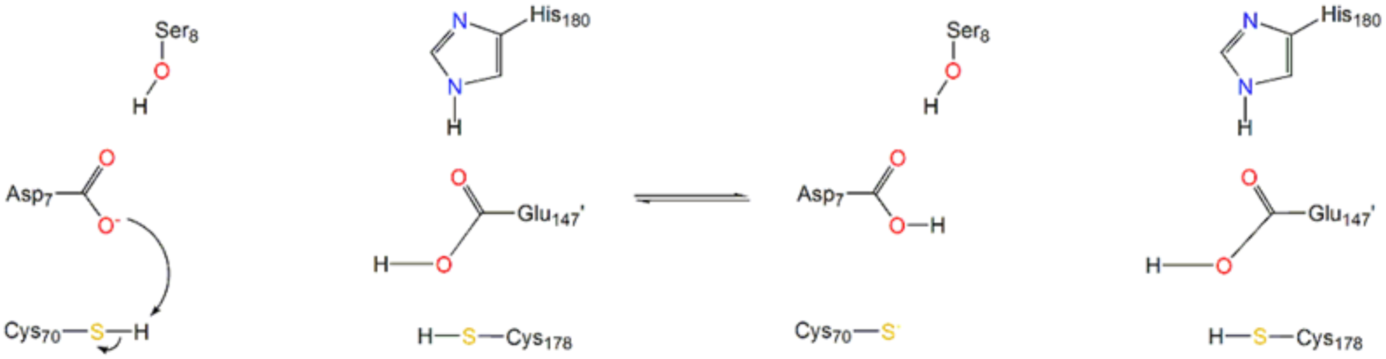

Mechanisms

Proton Transfer

Mechanism Components

Bond Cleavage
Bond Formation

Amino acids involved in the reaction step.

Amino Acid	Location of Function	Activity	Function
Cys178	Side Chain	spectator	Not Active
Ser8	Side Chain	spectator	Hydrogen Bond Donor<br>Activator
His180	Side Chain	spectator	Hydrogen Bond Donor<br>Electrostatic Stabiliser
Cys70	Side Chain	reactant	Proton Donor<br>Hydrogen Bond Donor
Glu147B	Side Chain	spectator	Hydrogen Bond Acceptor
Asp7	Side Chain	reactant	Proton Acceptor<br>Hydrogen Bond Acceptor

Reactive Centre

Bonds Formed	Bonds Cleaved	Bonds Changed in Order	Atom Types Involved
O-H	S-H	None	S<br>H<br>O

Difficulties of Hierarchical Classification

- Very similar chemical mechanisms can end up in different first level classes.
- In the case of phosphoinositide-specific phospholipases C, this is due to a slow final hydrolysis step occurring in one of the two enzymes.

Classifying Related Enzymes: Phosphoinositide-specific Phospholipases C

Eukaryotic (rat)	Prokaryotic (B. cereus)
Cell Signalling	Virulence factor
Multidomain	Single domain
Catalytic TIM Barrel	Catalytic TIM Barrel
EC 3.1.4.11	EC 4.6.1.13
Hydrolase	Lyase
Final hydrolysis step	No/slow final hydrolysis
Prefers bisphosphate	Disfavours bisphosphate
Acid-base mechanism	Acid-base mechanism
Calcium dependent	Not calcium dependent
Evolutionarily related	

Similar reactions end up far apart

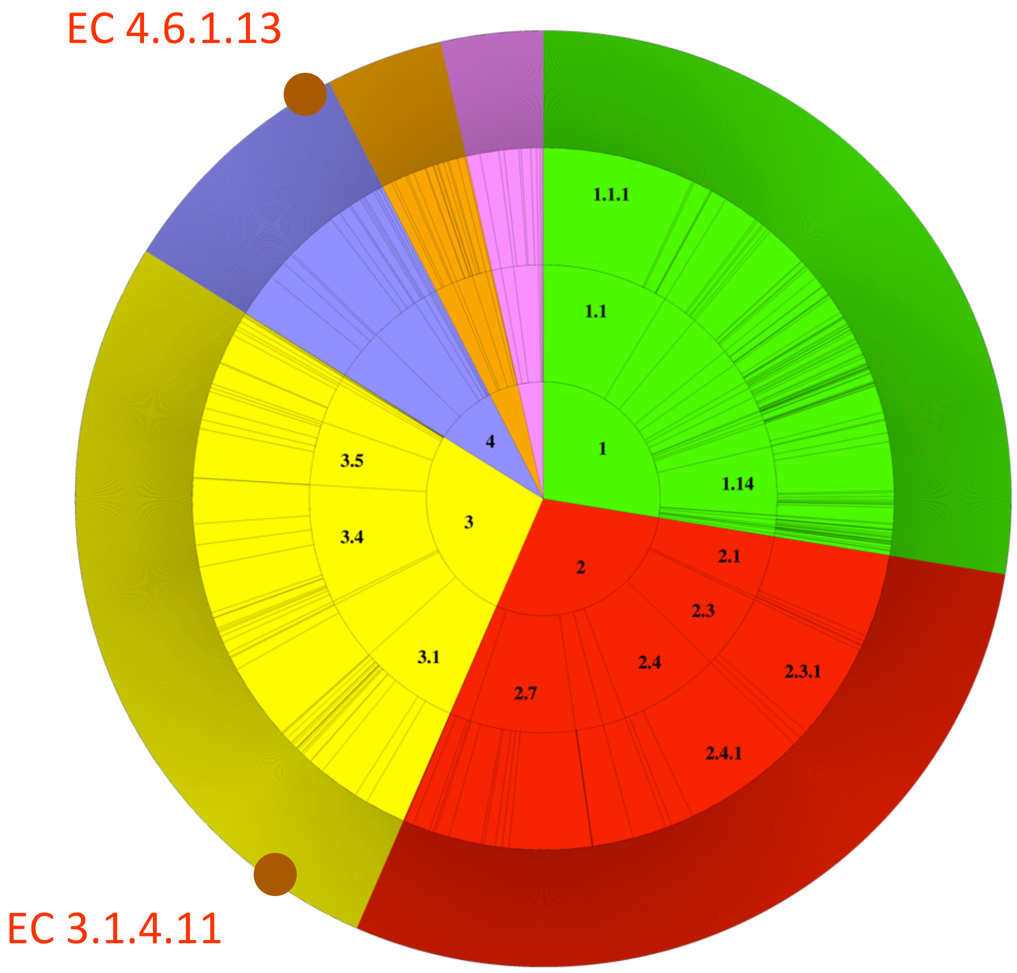

Difficulties of Hierarchical Classification

- Different chemical mechanisms can occur with exactly the same EC number.
- MACiE has six beta-lactamases, all with different mechanisms but the same overall reaction.

MACiE Entries by EC Code

Click on the MACiE Identifier number to view the entry. All other accession codes will take you to the relevant resource, e.g. the PDB code will link you into the PDBe entry for that PDB code.

Please note: CATH domains highlighted in bold are the catalytic domains

Entry Number	Enzyme Name	EC Code	PDB Code	CATH Code
M0098	peptide deformylase	3.5.1.88	1bsz	**3.90.45.10**<br>3.90.45.10
M0002	beta-lactamase (Class A)	3.5.2.6	1btl	**3.40.710.10**
M0015	beta-lactamase (Class B)	3.5.2.6	1znb	**3.60.15.10**
M0016	beta-lactamase (Class B)	3.5.2.6	1bc2	**3.60.15.10**
M0210	beta-lactamase (Class D)	3.5.2.6	1m6k	**3.40.710.10**
M0257	beta-lactamase (Class C)	3.5.2.6	1xx2	**3.40.710.10**
M0258	beta-lactamase (Class B)	3.5.2.6	1sml	**3.60.15.10**
M0096	creatinase	3.5.3.3	1chm	**3.90.230.10**<br>3.40.350.10<br>3.40.350.10
M0097	cytidine deaminase	3.5.4.5	1ctt	**3.40.140.10**<br>3.40.140.10

MACiE Mechanisms are Sourced from the Literature

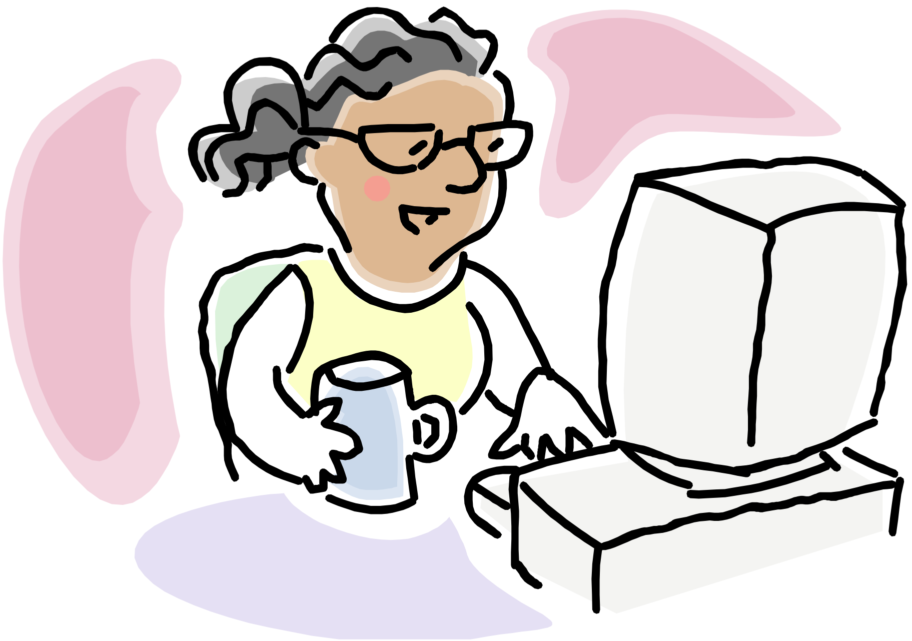

Residue Catalytic Propensities

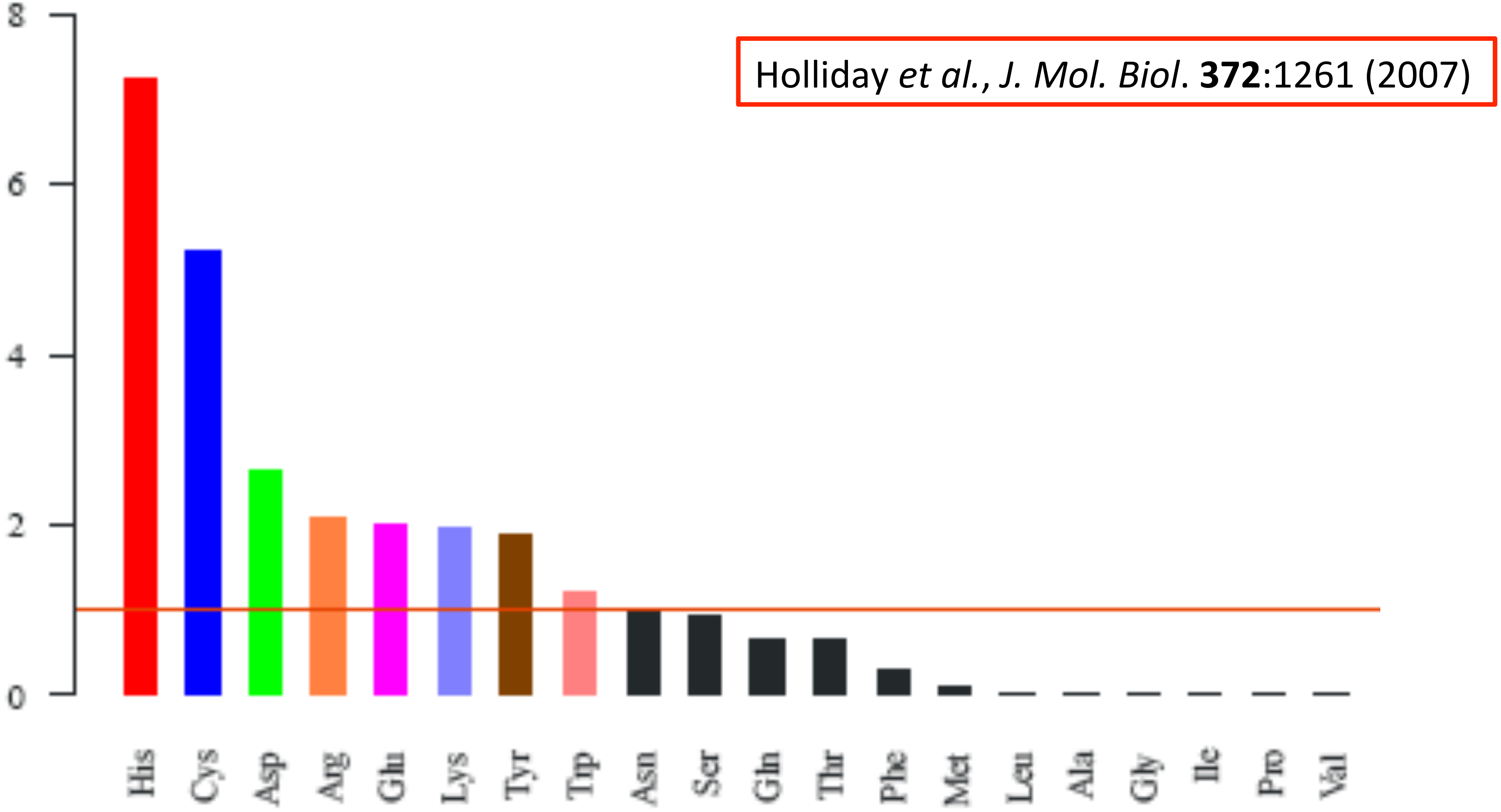

Holliday *et al.*, *J. Mol. Biol*. **372**:1261 (2007)

Residue Catalytic Functions

Holliday *et al.*, *J. Mol. Biol.* **390**:560 (2009)

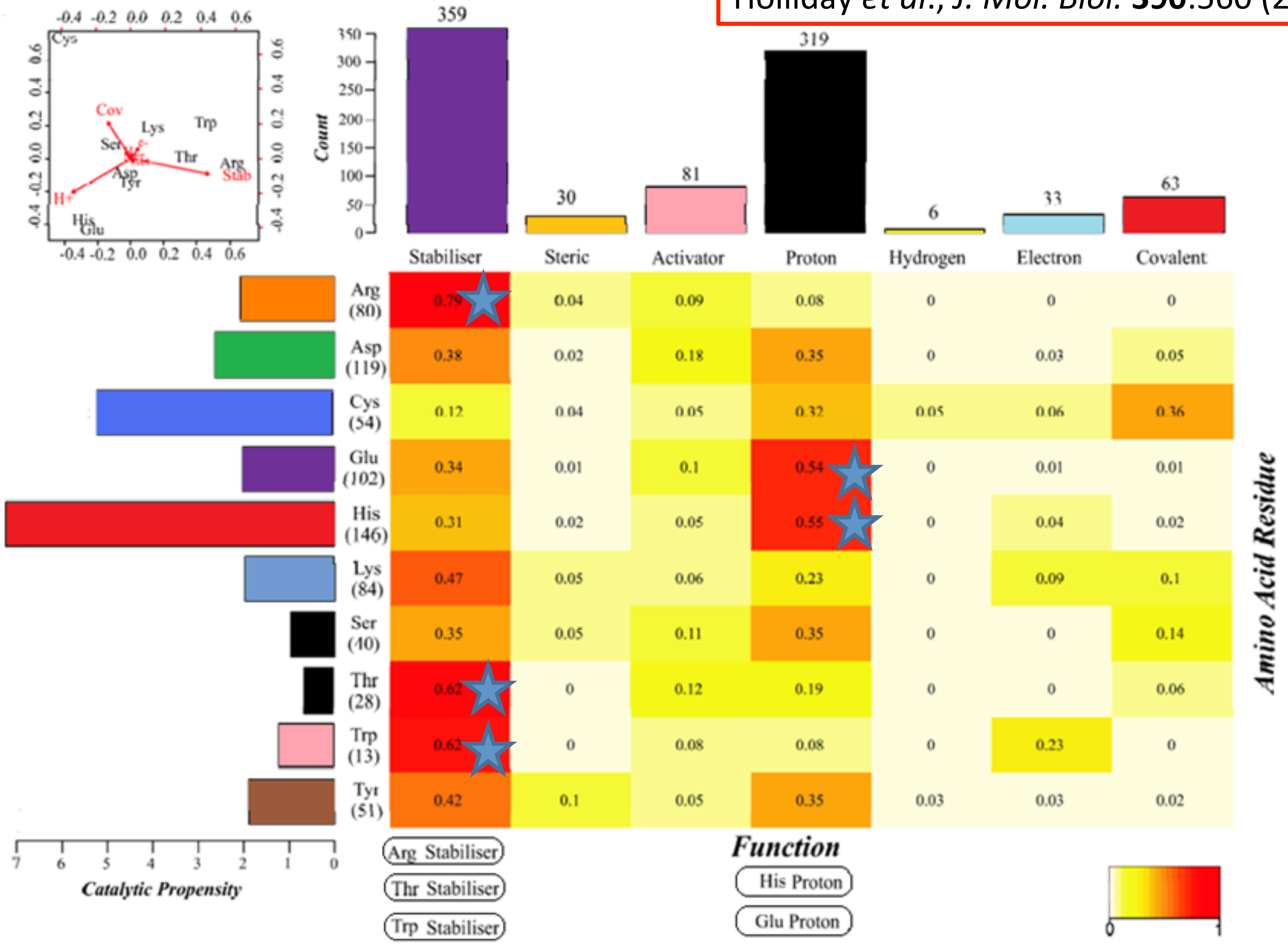

Convergently Evolved Reactions

- Study of 95 analogous pairs carrying out similar functions (EC 1.2.3.- conserved) ...

  ... and where enzymes are not homologous.

- Often catalyse similar chemical reactions by very different chemical mechanisms.

Almonacid *et al.*, *PLoS Computational Biology* **6**:e1000700 (2010)

Predicting Enzyme Function …
… and Mechanism

L. De Ferrari *et al. BMC Bioinformatics* **13**:61 (2012)

Predicting enzyme **function** from sequence

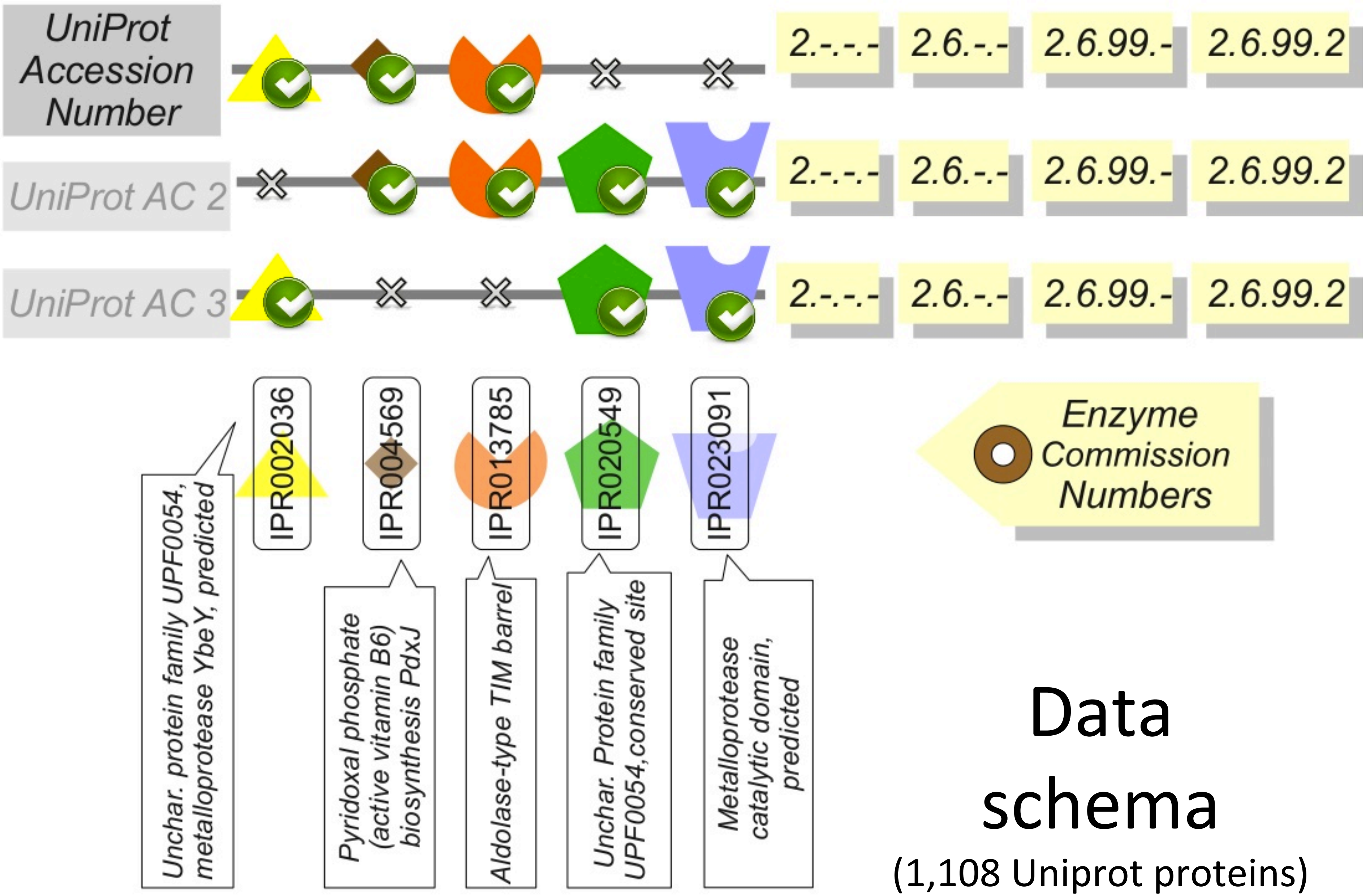

Data schema

(1,108 Uniprot proteins)

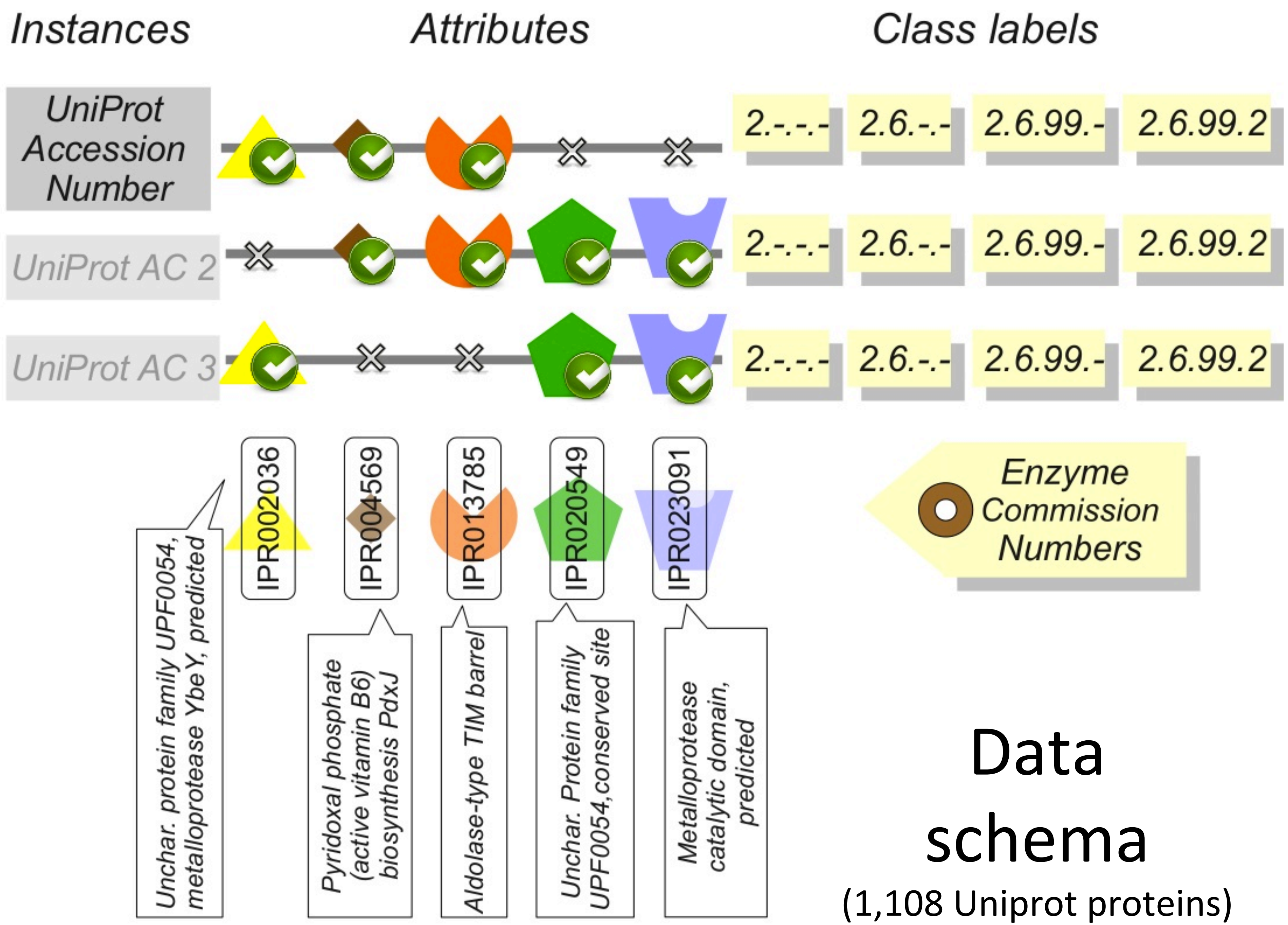

Data schema

(1,108 Uniprot proteins)

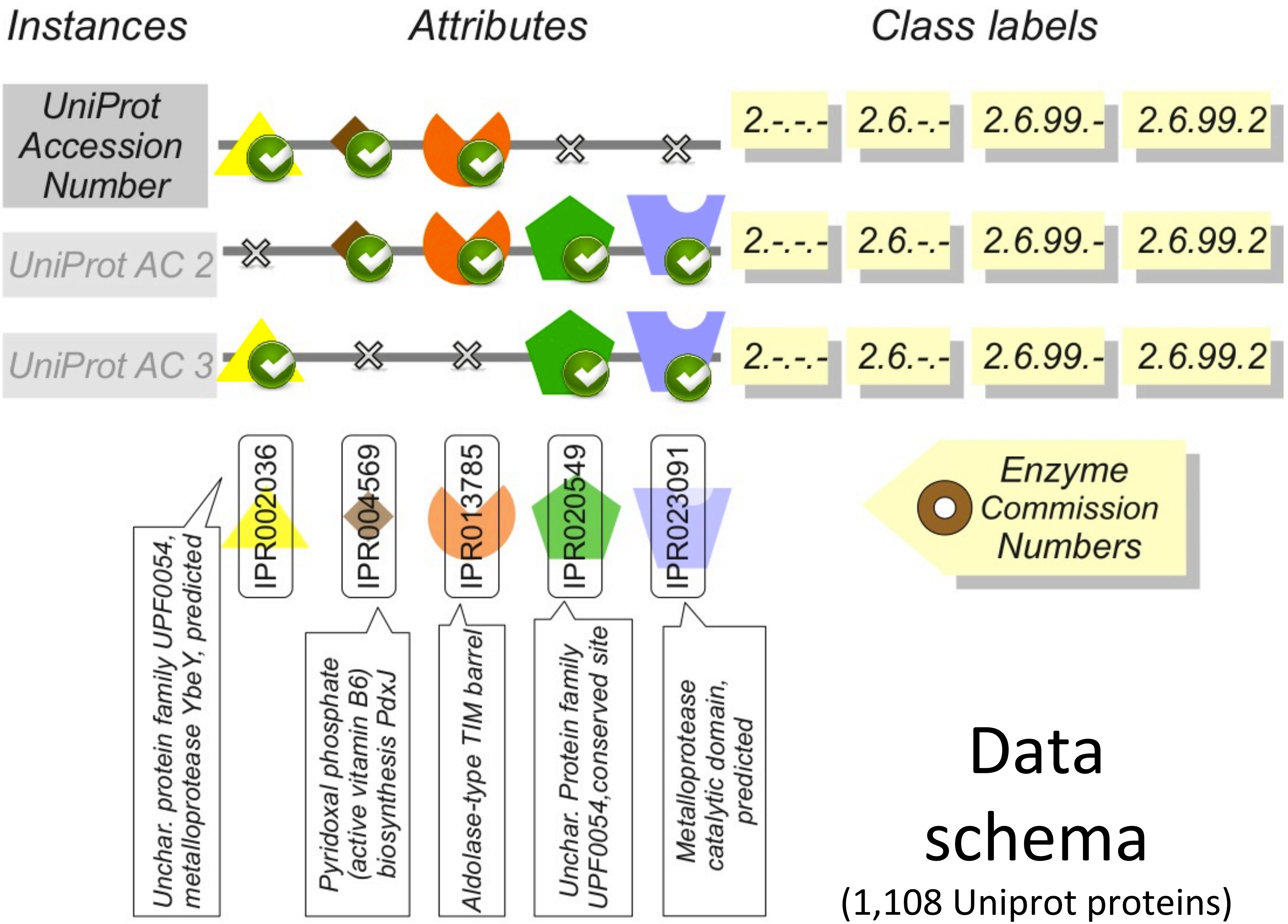

Data schema

(1,108 Uniprot proteins)

Plentiful data sources for function prediction

Predicting enzyme **mechanism** from sequence

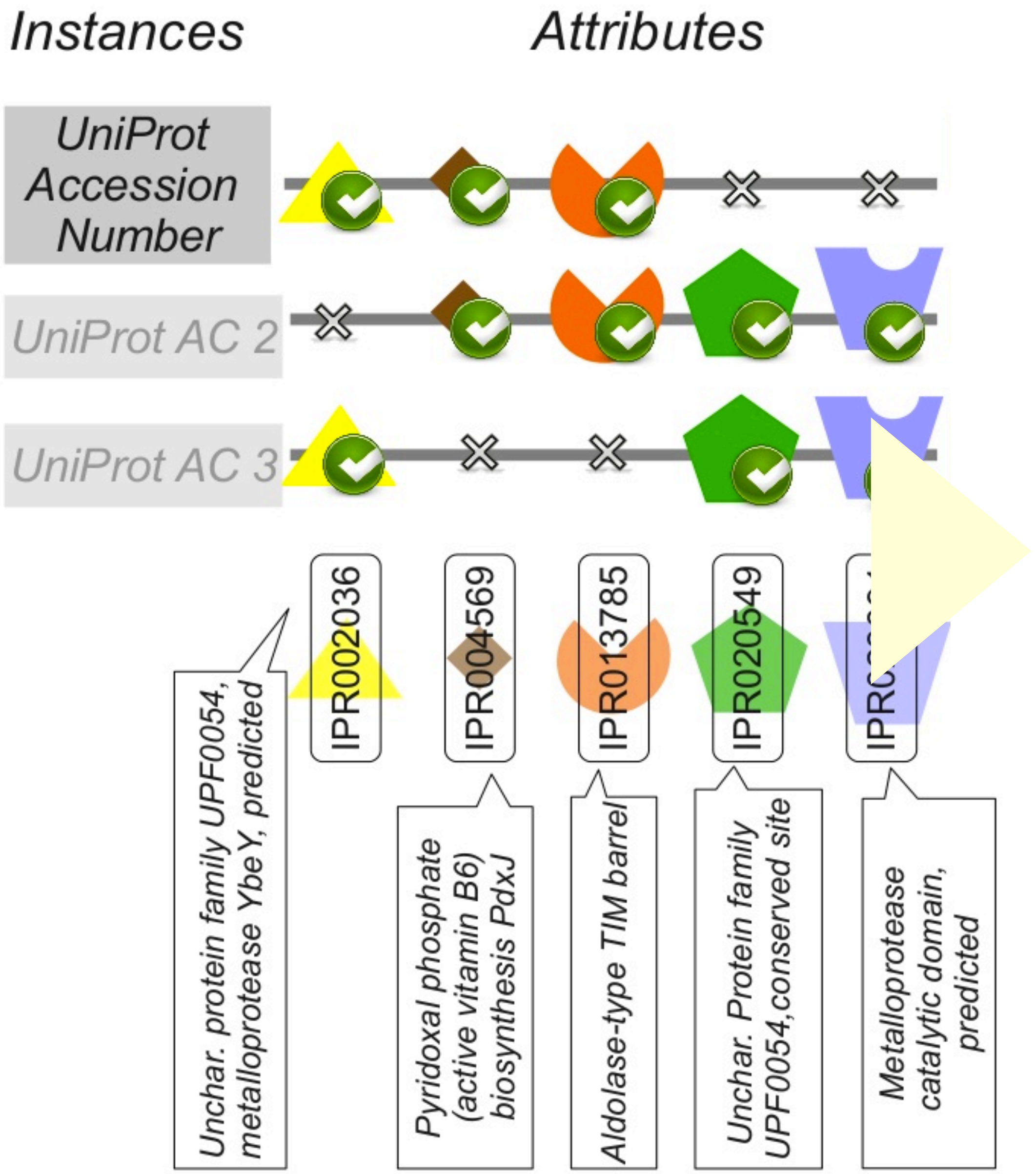

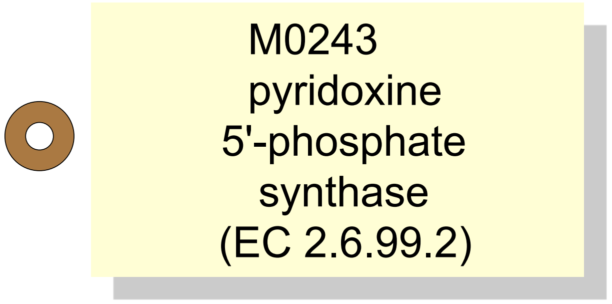

Far fewer validated data available for mechanism

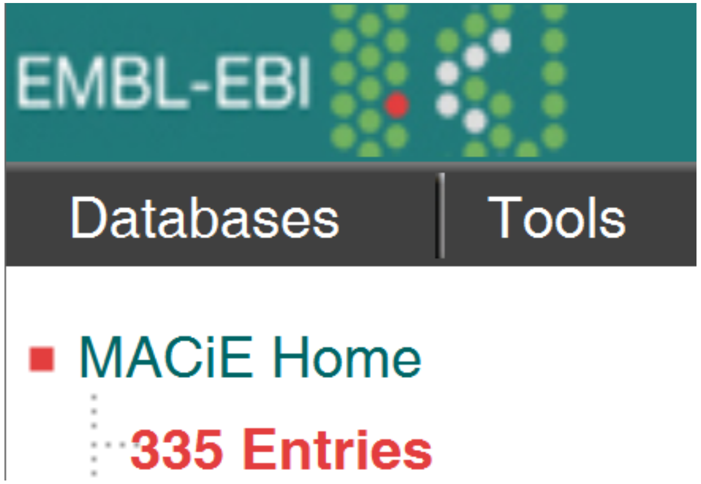

Current Enzyme Science

- Test theories of enzyme evolution
- Classify enzyme functions
- Understand enzymes' chemical mechanisms
- Reconstruct evolutionary history of enzymes
- Predict catalytic function of a sequence
- Design improved or novel enzymes
- Enzymes in industry
- Nearly half of pharmaceuticals target enzymes
- Address antibiotic resistance
