# Supplementary material for: 4273π: Bioinformatics education on low cost ARM hardware
Source: BMC Bioinformatics. 2013 Aug 12;14:243. doi: 10.1186/1471-2105-14-243 (PMC3751261; doi:10.1186/1471-2105-14-243)
Supplement: Additional file 2 — 4273π Bioinformatics for Biologists teaching material, Version 1.01. The module handbook, lectures and practicals are included. The latest version, including Linux, software and BLAST databases, is available at the 4273π Web site [25]. [file 1471-2105-14-243-S2.zip › 4273pi_course_material/week6/practical_genome_annotation.pdf]

4273π Bioinformatics for Biologists

Practical, Week 6: Genome Annotation

Daniel Barker, School of Biology, University of St Andrews
Email db60@st-andrews.ac.uk

© 2013 D. Barker. This is an Open Access document distributed under the terms of the Creative Commons Attribution License (http://creativecommons.org/licenses/by/2.0), which permits unrestricted use, distribution, and reproduction in any medium, provided the original work is properly cited.

4273π, Version 1.01. http://eggg.st-andrews.ac.uk/4273pi

This practical session involves DNA sequence analysis, specifically *primary genome annotation*. The learning objectives are:

- To perform and understand primary genome annotation (e.g. see Rajandream 2007) using sequence similarity, homology-based annotation software, and *de novo* annotation software.
- To sketch predicted gene structure, indicating coordinates and strand of exons on the genomic DNA.

Introduction

Prokaryotic genome annotation is relatively straightforward, due to the short intergenic spaces and absence of introns. Eukaryotic genome annotation, in contrast, is far more difficult. Eukaryotic genomes are larger, with large spaces between genes, and potentially large introns. Non-functioning gene fragments and pseudogenes abound, and tend to confuse genome annotation procedures. Because we like a challenge, this practical will focus on eukaryotic genome annotation.

Olfactory receptors (ORs) are involved in the sense of smell. They are part of the machinery which detects small molecules from the air, and generates neural signals within the animal. ORs are potentially important in mate recognition. The presence/absence of specific ORs, and the number of copies if present, can vary considerably even between closely related species.

Or98b (Flybase FBgn0039582) codes for an olfactory receptor in *Drosophila melanogaster*. We will seek its most similar homologue in the genome of *D. sechellia,* using three genome annotation techniques:

(1) BLAST (sequence similarity; Altschul et al. 1997, Korf 2005);
(2) GeneWise (sequence similarity and gene model; Birney et al. 2004); and
(3) SNAP (*de novo* genome annotation; Korf 2004).

Install software

Go to:

http://korflab.ucdavis.edu/software.html

Download SNAP. At the time of writing, the file is named `snap-2013-02-16.tar.gz`. Put it in the `~/4273pi/week6` directory. At the command prompt in LXTerminal, change to this directory. Then, uncompress SNAP:

```
tar xvzf snap-2013-02-16.tar.gz
```

The software is provided as source code, i.e. human-readable text, in the C programming language. This has to be translated into machine executable form, a process known as *compilation*. In the case of SNAP, this is achieved conveniently by `make`. Enter the following commands:

```
cd snap
make
```

Now edit your `~/.bashrc` text file, adding the following lines at the end. The `ZOE` environment variable is SNAP's means of finding data it requires to run.

```
PATH=${PATH}:${HOME}/4273pi/week6/snap
export PATH
ZOE=${HOME}/4273pi/week6/snap
export ZOE
```

Close LXTerminal. Open a new LXTerminal, and you should find the command `snap` is now available at the command line. Try it. Without any command-line arguments, it will give you a brief message on usage.

TBLASTN

Go to Flybase:

http://flybase.org

Search for FBgn0039582. Paste the translation of its coding sequence (CDS) into a text file, in Fasta format.

On the Flybase Web site, BLAST this protein against the *D. sechellia* genome assembly using TBLASTN. This searches a nucleotide database in translation, for homologues of a protein query. TBLASTN is only looking for sequence similarity. It has no model of gene structure in mind. It is a simple, but important tool in genome annotation.

→ In which genomic DNA fragment (‘scaffold’) does the closest *D. sechellia* homologue appear to be found?

→ Does any aspect of the BLAST results alarm you? (If not, return to this question later, after using the other genome annotation techniques.)

→ Sketch the structure of this homologue, linking together nearby BLAST matches as ‘exons’. Represent the scaffold as a single line. Draw exons on this as boxes, linked by another line. Indicate the coordinates of the start and end of each exon, and label the start codon clearly. Indicate the direction (strand) of the ‘gene’ on the DNA fragment by means of an arrow. The sketch does not have to be to scale.

Leave the FlyBase browser window open, you will refer to it again below.

GeneWise

GeneWise combines aspects of BLAST (sequence similarity search) with an attempt to model gene structure. For example, rather than simply seek similarity between a protein and DNA, it also penalises stop codons within the ‘coding sequence’, and expects splice sites to be ‘reasonable’. This makes it a far better program than BLAST for obtaining predictions of gene structure, though it runs more slowly than BLAST. However, genome annotation is difficult and no such program is perfect.

From the Flybase BLAST results report, for a BLAST match involving your putative gene, click ‘GBrowse’. This takes you to a graphical view of the genomic region in *D. sechellia*. Obtain the two kilobases (2 kbp) of genomic DNA involving this putative gene (i.e. the potential Or98b homologue you have proposed on the basis of TBLASTN). Save this as a text file in Fasta format.

Although GeneWise may be downloaded to run on your own computer, I have not yet been able to make it work on the Raspberry Pi. We will run it online at the European Bioinformatics Institute:

http://www.ebi.ac.uk/Tools/psa/genewise

Browse to find your *D. melanogaster* protein and *D. sechellia* genomic DNA files, and submit. The output shows the query protein, predicted protein, and predicted codons.

→ Sketch the structure of this predicted gene.

Look very carefully at the homologue predicted by GeneWise, in particular the region near the start of exon three (i.e. just after intron 2).

→ Bring the TBLASTN result back on-screen. In this region, how does the gene predicted by GeneWise in *D. sechellia* differ from the 'gene' predicted by TBLASTN?

SNAP

SNAP is a *de novo* genome annotation program. In contrast to BLAST and GeneWise, it doesn't use sequence similarity. Hence, no protein is required as input. Combined with its speed, this makes it a very convenient way to annotate genomes. Also, since it does not require a gene to have similarity to a known protein (or RNA) sequence, it may find genes for which no homologue is yet known. These are advantages.

The disadvantages of *de novo* genome annotation programs are that they are not so accurate. They have a reputation for being confused by pseudogenes, for example. Single-exon gene predictions often turn out to be **processed pseudogenes**. These have been inserted in the genome by retrotransposition, and are non-functional due to lack of a promoter. (There *are* bona fide single-exon genes in eukaryotic genomes – but in the human genome, for example, they are rare.) Even a gene prediction with introns may be incorrect – software may falsely add a short intron around any disabling mutation (frameshift, or substitution leading to a premature stop codon), to predict something that looks like a gene where it should not.

Many bioinformatic programs build up a 'model' of what they're looking for. Building up the model, or *training* it, involves presenting the program with positive and negative examples of what to looking for (e.g. genes as positive examples; intergenic DNA and pseudogenes as negative examples). The program will summarize the salient features of the positive and negative data sets, and use matches to these features to make future predictions.

SNAP has already been trained to predict genes for several organisms, separately. This is likely to be more accurate than a single model for all organisms, since different species have different codon usage, different intron lengths, etc. It makes sense to use a SNAP model either for the species whose DNA we're looking at, or a close relative. SNAP has not been trained for *D. sechellia*, but has been trained for the close relative *D. melanogaster*. This model is referred to by SNAP as 'fly'.

Expand an LXTerminal window horizontally so more text can be shown per line. Then, predict genes in the *D. sechellia* DNA you downloaded, using SNAP's *D. melanogaster* model, as follows, substituting the name of the genomic DNA file for *DNA_file*:

`snap fly` *DNA_file*

Of the main part of SNAP's output, the first four columns are the most important. These columns are as follows:

(1) **Feature**. '**Einit**', initial exon of a gene; '**Eterm**', final exon of a gene; '**Exon**', internal (i.e. not initial or final) exon of a gene; '**Esngl**', single-exon gene.
(2) **Start co-ordinate** (base position) on the genomic DNA.
(3) **End co-ordinate** (base position) on the genomic DNA.
(4) **Strand** on the input DNA. '+', forward; '-', reverse.

Sketch the structure of the gene(s) predicted by SNAP.

→ Compare the predictions obtained from SNAP, GeneWise and BLAST. Where and why do they differ? What do you conclude about the homologue of *D. melanogaster* Or98b in *D. sechellia*?

→ Return to FlyBase and look again at the region of the *D. sechellia* involved in your original TBLASTN alignment with Or98b. FlyBase shows gene predictions of its own. How do they relate to your results?

→ If time permits, use the FlyBase Web site and/or publications to discover how the predictions were made, and relate this to your results.

→ In your own time, use SNAP to annotate a larger region of genomic DNA from *D. sechellia*. Practice sketching gene structure, including genes on the reverse strand of the genomic DNA. Compare results with those available in FlyBase.

Acknowledgements

I am grateful to Anastasia Gardiner (Université Lyon 1) for suggesting the Or98b *Drosophila melanogaster–D. sechellia* case study.

References

Altschul, S.F., Madden, T.L., Schäffer, A.A., Zhang, J., Zhang, Z., Miller, W. and Lipman, D.J. (1997) Gapped BLAST and PSI-BLAST: a new generation of protein database search programs. *Nucleic Acids Research* 25: 3389-3402.

Birney, E., Clamp, M. and Durbin, R. (2004) GeneWise and Genomewise. *Genome Research* 14: 988-995.

Korf, I. (2004) Gene finding in novel genomes. *BMC Bioinformatics* 5: 59.

Korf, I., Yandell, M. and Bedell, J. (2005) *BLAST* (Sebastopol, California: O'Reilly).

Rajandream, M.-A. (2007) Gene prediction. In Dear, P.H., editor, *Bioinformatics* (Bloxham, Oxfordshire: Scion), pp. 71-102.
