# Supplementary material for: 4273π: Bioinformatics education on low cost ARM hardware
Source: BMC Bioinformatics. 2013 Aug 12;14:243. doi: 10.1186/1471-2105-14-243 (PMC3751261; doi:10.1186/1471-2105-14-243)
Supplement: Additional file 2 — 4273π Bioinformatics for Biologists teaching material, Version 1.01. The module handbook, lectures and practicals are included. The latest version, including Linux, software and BLAST databases, is available at the 4273π Web site [25]. [file 1471-2105-14-243-S2.zip › 4273pi_course_material/week6/lecture_BLAST_and_DNA.pdf]

BLAST and DNA Sequence Analysis

4273π Bioinformatics for Biologists

Lecture, Week 6

Daniel Barker, School of Biology, University of St Andrews

Email db60@st-andrews.ac.uk

© 2013 D. Barker. This is an Open Access document distributed under the terms of the Creative Commons Attribution License (http://creativecommons.org/licenses/by/2.0), which permits unrestricted use, distribution, and reproduction in any medium, provided the original work is properly cited.

4273π, Version 1.01. http://eggg.st-andrews.ac.uk/4273pi

1. BLAST

What is BLAST doing?

- Lining up a query sequence against sequences in a database to find (sub) sequences which have higher similarity to the query than expected by chance.
- BLAST is one of various programs for *pairwise sequence alignment*.

Dot-matrix pairwise alignment

- Make a table:
- one sequence along the top, one along the left.
- Put a dot in each cell where the sequences have the same residue.
- Join adjacent cells to make a pairwise alignment.
- The ‘ideal’ alignment would be an unbroken diagonal line from top left to bottom right.
- The actual, optimal alignment, for the sequences in question, would be as close to this as possible.

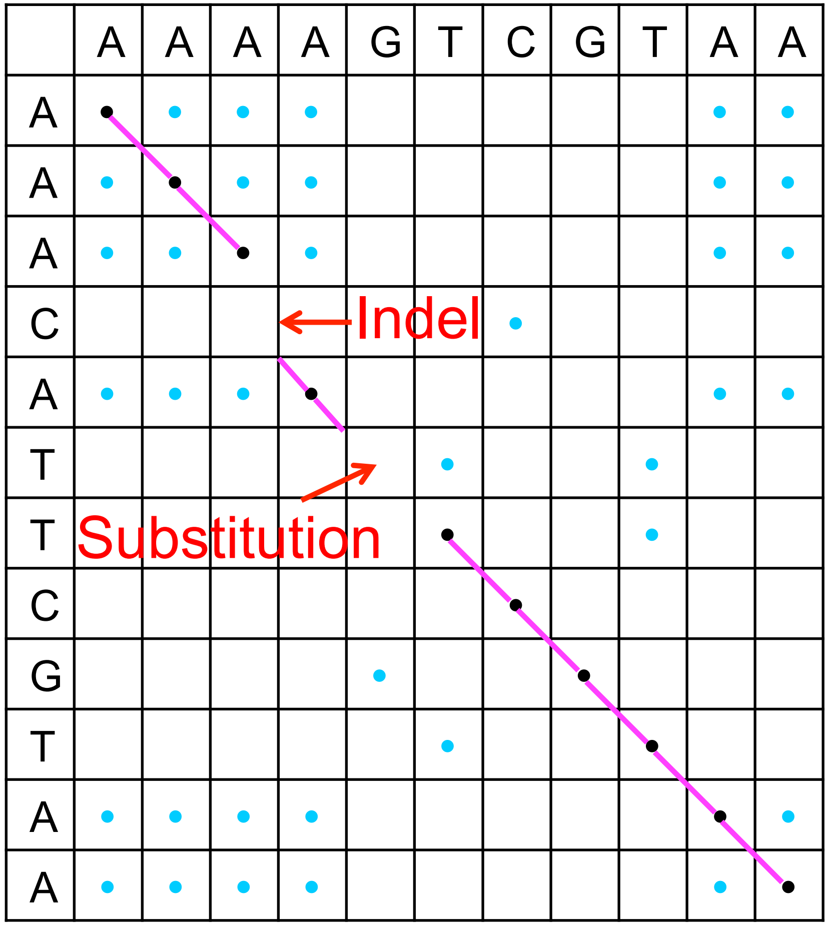

Partial sequences of 18S rDNA in human (accession NR_003286.2, top) and 18S rDNA in *Aspergillus fumigatus* (FJ214371.1, left)

```
Upper sequence:  AAA-AGTCGTAA
                 ||| | ||||||
 Left sequence:  AAACATTCGTAA
```

Global pairwise alignment

- Needleman-Wunsch alignment.
- Align two sequences along their full length, in the best possible way (even if the quality of the alignment is very poor).
- Rarely used
  - *Multiple* alignment, however, often is global (e.g. Clustal software).
- Software for pairwise global alignment: EMBOSS package (needle).

Local pairwise alignment

- Find *local* regions of similarity.
- Where sequences are not similar, no alignment is reported.
- Where only parts of sequences are similar, only parts of sequences are aligned.
- Frequently used.
- Software: e.g. EMBOSS package (water).

Substitution matrices

E.g. BLOSUM62

	A	R	N	D	C	Q	E	G	H	I	L	K	M	F	P	S	T	W	Y	V	B	Z	X	*
**A**	4	-1	-2	-2	0	-1	-1	0	-2	-1	-1	-1	-1	-2	-1	1	0	-3	-2	0	-2	-1	0	-4
**R**	-1	5	0	-2	-3	1	0	-2	0	-3	-2	2	-1	-3	-2	-1	-1	-3	-2	-3	-1	0	-1	-4
**N**	-2	0	6	1	-3	0	0	0	1	-3	-3	0	-2	-3	-2	1	0	-4	-2	-3	3	0	-1	-4
**D**	-2	-2	1	6	-3	0	2	-1	-1	-3	-4	-1	-3	-3	-1	0	-1	-4	-3	-3	4	1	-1	-4
**C**	0	-3	-3	-3	9	-3	-4	-3	-3	-1	-1	-3	-1	-2	-3	-1	-1	-2	-2	-1	-3	-3	-2	-4
**Q**	-1	1	0	0	-3	5	2	-2	0	-3	-2	1	0	-3	-1	0	-1	-2	-1	-2	0	3	-1	-4
**E**	-1	0	0	2	-4	2	5	-2	0	-3	-3	1	-2	-3	-1	0	-1	-3	-2	-2	1	4	-1	-4
**G**	0	-2	0	-1	-3	-2	-2	6	-2	-4	-4	-2	-3	-3	-2	0	-2	-2	-3	-3	-1	-2	-1	-4
**H**	-2	0	1	-1	-3	0	0	-2	8	-3	-3	-1	-2	-1	-2	-1	-2	-2	2	-3	0	0	-1	-4
**I**	-1	-3	-3	-3	-1	-3	-3	-4	-3	4	2	-3	1	0	-3	-2	-1	-3	-1	3	-3	-3	-1	-4
**L**	-1	-2	-3	-4	-1	-2	-3	-4	-3	2	4	-2	2	0	-3	-2	-1	-2	-1	1	-4	-3	-1	-4
**K**	-1	2	0	-1	-3	1	1	-2	-1	-3	-2	5	-1	-3	-1	0	-1	-3	-2	-2	0	1	-1	-4
**M**	-1	-1	-2	-3	-1	0	-2	-3	-2	1	2	-1	5	0	-2	-1	-1	-1	-1	1	-3	-1	-1	-4
**F**	-2	-3	-3	-3	-2	-3	-3	-3	-1	0	0	-3	0	6	-4	-2	-2	1	3	-1	-3	-3	-1	-4
**P**	-1	-2	-2	-1	-3	-1	-1	-2	-2	-3	-3	-1	-2	-4	7	-1	-1	-4	-3	-2	-2	-1	-2	-4
**S**	1	-1	1	0	-1	0	0	0	-1	-2	-2	0	-1	-2	-1	4	1	-3	-2	-2	0	0	0	-4
**T**	0	-1	0	-1	-1	-1	-1	-2	-2	-1	-1	-1	-1	-2	-1	1	5	-2	-2	0	-1	-1	0	-4
**W**	-3	-3	-4	-4	-2	-2	-3	-2	-2	-3	-2	-3	-1	1	-4	-3	-2	11	2	-3	-4	-3	-2	-4
**Y**	-2	-2	-2	-3	-2	-1	-2	-3	2	-1	-1	-2	-1	3	-3	-2	-2	2	7	-1	-3	-2	-1	-4
**V**	0	-3	-3	-3	-1	-2	-2	-3	-3	3	1	-2	1	-1	-2	-2	0	-3	-1	4	-3	-2	-1	-4
**B**	-2	-1	3	4	-3	0	1	-1	0	-3	-4	0	-3	-3	-2	0	-1	-4	-3	-3	4	1	-1	-4
**Z**	-1	0	0	1	-3	3	4	-2	0	-3	-3	1	-1	-3	-1	0	-1	-3	-2	-2	1	4	-1	-4
**X**	0	-1	-1	-1	-2	-1	-1	-1	-1	-1	-1	-1	-1	-1	-2	0	0	-2	-1	-1	-1	-1	-1	-4
*****	-4	-4	-4	-4	-4	-4	-4	-4	-4	-4	-4	-4	-4	-4	-4	-4	-4	-4	-4	-4	-4	-4	-4	1

Source: EMBOSS package, http://emboss.sourceforge.net

Short Cuts

- *Heuristic* local alignment is *faster.*
- Ignore regions of the dot-matrix alignment table which are unlikely to be high-scoring.
- Done rationally, heuristic local alignment is almost as good as Smith-Waterman alignment.
- Very frequently used.
- Software: e.g. BLAST, Fasta.

Protein vs nucleotide alignment

- Always compare sequences at the protein level *where possible*.
- Protein comparisons are more sensitive -
    - 20 residues (protein) *vs* 4 (nucleotide).
    - Often very high cross-species conservation (protein) *vs* lower cross-species conservation (DNA).

Use the correct version of BLAST for the question at hand

- You can get BLAST to translate nucleotide queries and/or databases, in all six conceivable reading frames (TBLASTN, BLASTX, TBLASTX).
- Where DNA is non-coding, use nucleotide BLAST (e.g. BLASTN)
  - will search for matches between either strand of query and database sequences
  - pays no attention to any reading frames.

Main BLAST programs

Program	Query	Database	Comparison
BLASTP	protein	protein	protein
TBLASTN	protein	nucleotide	protein
BLASTX	nucleotide	protein	protein
TBLASTX	nucleotide	nucleotide	protein
BLASTN	nucleotide	nucleotide	nucleotide
PSI-BLAST	protein	protein	protein
MEGABLAST	nucleotide	nucleotide	nucleotide

2. DNA Sequence Analysis

Why?

- Protein sequence fully specifies its function and provides clues to its evolutionary history.
- Thus, sequence analysis provide clues to protein function.
- Similarly, DNA sequence fully specifies its function
  - E.g. DNA: gene, promoter, enhancer, exon, intron.
  - E.g. RNA secondary structure.

Primary genome annotation

- Exons might occupy only approx. 2% of the genome of a eukaryote (varying considerably from species to species).
- How can we find the genes within the sequence of the genomic DNA?
- How can we distinguish introns, exons and inter-genic space?
- How can we distinguish coding sequence from non-coding sequence?

Example: some yeast genomic DNA - where is the gene?

```
ATTACCACCGATTAAGGAAGGCTGTATAACCGAACAGGTACGCGAGTTCTATGGACTTGAATCAAAGAAAGGAAAAAAAG
GGCCAGCATGTTGGATGCTGTGGCTCCAGGACAGACCTGTCTGCTGACACAGTGGAACTGATAGAAAGAATGGACAGATT
GGCTGAAAATCAGGCGACAGCTTCCATGTCGATCGTTGCGTTACCGTCTAGCTTCCAGGAGAGCAATAGCAGTGACAGGT
GCAGAAAGTATTGCAGCAGTGATGAGGACAGCGACACGTGCATTCATGGTAGTGCTAATGCCAGTACCAATGCGACTACC
AACTCCAGCACTAATGCTACTACCACTGCCAGCATCAACGTCAGGACTAGTGCGACTACCACTGCCAGCATCAACGTCAG
GACTAGTGCGACTACCACTGAAAGTACCAACTCCAACACTAATGCTACTACCACTGAAAGTACCAACTCCAGCACTAATG
CTACTACCACTGCCAGCATCAACGTCAGGACTAGTGCGACTACCACTGAAAGTACCAACTCCAGCACTAATGCTACTACC
ACTGCCAGCATCAACGTCAGGACTAGTGCGACTACCACTGAAAGTACCAACTCCAGCACTAATGCTACTACCACTGCCAG
CATCAACGTCAGGACTAGTGCGACTACCACTGAAAGTACCAACTCCAACACTAATGCCAGTACCAATGCGACTACCAACT
CCAGCACTAATGCTACTACCACTGCCAGCACCAACGTCAGGACTAGTGCTACTACCAATGCGACTACCAACTCCAGCACT
AATGCTACTACCACTGCCAGCACCAACGTCAGGACTAGTGCTACTACCACTGCCAGCACCAACGTCAGGACTAGTGCTAC
TACCACTGCCAGCATCAACGTCAGGACTAGTGCTACTACCACTGAAAGTATCAACTCTAGCACTAATGCTACTACCACTG
AAAGTACCAACTCCAACACTAGTGCTACTACCACCGAAAGTACCGACTCCAACACTAATGCTACTACCACTGCTAGCATC
AACGTCAGGACTAGTGCGACTACCACTGAAAGTACCAACTCCAACACTAGTGCTACTACCACCGAAAGTACCGACTCCAA
CACTAGTGCTACTACCACTGCTAGCACCAACTCCAGCACTAATGCCACTACCACTGCTAGCACCAACTCCAGCACTAATG
CCACTACCACTGAAAGTACCAACGCTAGTGCCAAGGAGGACGCCAATAAAGATGGCAATGCTGAGGATAATAGATTCCAT
CCAGTCACCGACATTAACAAAGAGTCGTATAAGCGGAAAGGGAGTCAAATGGTTTTCCTAGAGAGAAAGAAACTGAAAGC
ACAATTTCCCAATACTTCCGAGAATATGAATGTCTTACAGTTTCTTGGATTTCGGTCTGACGAAATTAAACATCTTTTCC
TCTATGGTATTGACATATACTTCTGCCCAGAGGGAGTATTCACACAATACGGATTATGCAAGGGCTGTCAAAAGATGTTC
GGGCTCTGTGTCTGTTGGGCTGGCCAGAAAGTATCGTATCGGAGGATAGCTTGGGAAGCACTAGCTGTGGAGAGAATGCT
GCGAAATGATGAGGAATACAAAGAATACTTGGAAGACATCGAGCCATATCATGGGGACCCTGTAGGATATTTGAAATATT
TTAGCGTAAAAAGGAGAGAGATCTACTCTCAGATACAGAGAAATTATGCTTGGTACCTGGCCATTACTAGAAGAAGAGAA
ACAATTAGTGTATTGGATTCGACAAGAGGCAAGCAAGGGAGCCAAGTTTTCCGCATGTCTGGAAGGCAGATCAAAGAGTT
GTATTATAAAGTATGGAGCAACTTGCGTGAATCGAAGACAGAGGTGCTGCAGTACTTTTTGAACTGGGACGAAAAAAAGT
GCCGGGAAGAATGGGAGGCAAAAGACGATACGGTCTTTGTGGAAGCGCTCGAGAAAGTTGGAGTTTTTCAGCGTTTGCGT
TCCATGACGAGCGCTGGACTGCAGGGTCCGCAGTACGTCAAGCTGCAGTTTAGCAGGCATCATCGACAGTTGAGGAGCAG
ATATGAATTAAGTCTAGGAATGCACTTGCGAGATCAGCTTGCGCTGGGAGTTACCCCATCTAAAGTGCCGCATTGGACGG
CATTCCTGTCGATGCTGATAGGGCTGTTCTACAATAAAACATTTCGGCAGAAACTGGAATATCTTTTGGAGCAGATTTCG
GAGATGTGGTTGTTACCACATTGGCTTGATTTGGCAAACGTTGAAGTTCTCGCTGCAGATAACACGAGGGTACCACTGTA
CATGCTGATGGTAGCGGTTCACAAAGAGCTGGATAGCGATGATGTTCCAGACGGTAGATTTGATATAATATTACTATGTA
GAGATTCGAGCAGAGAAGTTGGAGAGTGAAGGAAATTGTTGTTACGAAAGTCAGTGATTATGTATTGTGTAGTATAGTA
```

Source: *Saccharomyces cerevisiae* (Chromosome II) in Ensembl, http://www.ensembl.org

Gene

attaccaccgattaaggaaggctgtataaccgaacaggtacgcgagttct***ATG***GACTTGAATCAAAGAAAGGAAAAAAAG
GGCCAGCATGTTGGATGCTGTGGCTCCAGGACAGACCTGTCTGCTGACACAGTGGAACTGATAGAAAGAATGGACAGATT
GGCTGAAAATCAGGCGACAGCTTCCATGTCGATCGTTGCGTTACCGTCTAGCTTCCAGGAGAGCAATAGCAGTGACAGGT
GCAGAAAGTATTGCAGCAGTGATGAGGACAGCGACACGTGCATTCATGGTAGTGCTAATGCCAGTACCAATGCGACTACC
AACTCCAGCACTAATGCTACTACCACTGCCAGCATCAACGTCAGGACTAGTGCGACTACCACTGCCAGCATCAACGTCAG
GACTAGTGCGACTACCACTGAAAGTACCAACTCCAACACTAATGCTACTACCACTGAAAGTACCAACTCCAGCACTAATG
CTACTACCACTGCCAGCATCAACGTCAGGACTAGTGCGACTACCACTGAAAGTACCAACTCCAGCACTAATGCTACTACC
ACTGCCAGCATCAACGTCAGGACTAGTGCGACTACCACTGAAAGTACCAACTCCAGCACTAATGCTACTACCACTGCCAG
CATCAACGTCAGGACTAGTGCGACTACCACTGAAAGTACCAACTCCAACACTAATGCCAGTACCAATGCGACTACCAACT
CCAGCACTAATGCTACTACCACTGCCAGCACCAACGTCAGGACTAGTGCTACTACCAATGCGACTACCAACTCCAGCACT
AATGCTACTACCACTGCCAGCACCAACGTCAGGACTAGTGCTACTACCACTGCCAGCACCAACGTCAGGACTAGTGCTAC
TACCACTGCCAGCATCAACGTCAGGACTAGTGCTACTACCACTGAAAGTATCAACTCTAGCACTAATGCTACTACCACTG
AAAGTACCAACTCCAACACTAGTGCTACTACCACCGAAAGTACCGACTCCAACACTAATGCTACTACCACTGCTAGCATC
AACGTCAGGACTAGTGCGACTACCACTGAAAGTACCAACTCCAACACTAGTGCTACTACCACCGAAAGTACCGACTCCAA
CACTAGTGCTACTACCACTGCTAGCACCAACTCCAGCACTAATGCCACTACCACTGCTAGCACCAACTCCAGCACTAATG
CCACTACCACTGAAAGTACCAACGCTAGTGCCAAGGAGGACGCCAATAAAGATGGCAATGCTGAGGATAATAGATTCCAT
CCAGTCACCGACATTAACAAAGAGTCGTATAAGCGGAAAGGGAGTCAAATGGTTTTCCTAGAGAGAAAGAAACTGAAAGC
ACAATTTCCCAATACTTCCGAGAATATGAATGTCTTACAGTTTCTTGGATTTCGGTCTGACGAAATTAAACATCTTTTCC
TCTATGGTATTGACATATACTTCTGCCCAGAGGGAGTATTCACACAATACGGATTATGCAAGGGCTGTCAAAAGATGTTC
GGGCTCTGTGTCTGTTGGGCTGGCCAGAAAGTATCGTATCGGAGGATAGCTTGGGAAGCACTAGCTGTGGAGAGAATGCT
GCGAAATGATGAGGAATACAAAGAATACTTGGAAGACATCGAGCCATATCATGGGGACCCTGTAGGATATTTGAAATATT
TTAGCGTAAAAAGGAGAGAGATCTACTCTCAGATACAGAGAAATTATGCTTGGTACCTGGCCATTACTAGAAGAAGAGAA
ACAATTAGTGTATTGGATTCGACAAGAGGCAAGCAAGGGAGCCAAGTTTTCCGCATGTCTGGAAGGCAGATCAAAGAGTT
GTATTATAAAGTATGGAGCAACTTGCGTGAATCGAAGACAGAGGTGCTGCAGTACTTTTTGAACTGGGACGAAAAAAAGT
GCCGGGAAGAATGGGAGGCAAAAGACGATACGGTCTTTGTGGAAGCGCTCGAGAAAGTTGGAGTTTTTCAGCGTTTGCGT
TCCATGACGAGCGCTGGACTGCAGGGTCCGCAGTACGTCAAGCTGCAGTTTAGCAGGCATCATCGACAGTTGAGGAGCAG
ATATGAATTAAGTCTAGGAATGCACTTGCGAGATCAGCTTGCGCTGGGAGTTACCCCATCTAAAGTGCCGCATTGGACGG
CATTCCTGTCGATGCTGATAGGGCTGTTCTACAATAAAACATTTCGGCAGAAACTGGAATATCTTTTGGAGCAGATTTCG
GAGATGTGGTTGTTACCACATTGGCTTGATTTGGCAAACGTTGAAGTTCTCGCTGCAGATAACACGAGGGTACCACTGTA
CATGCTGATGGTAGCGGTTCACAAAGAGCTGGATAGCGATGATGTTCCAGACGGTAGATTTGATATAATATTACTATGTA
GAGATTCGAGCAGAGAAGTTGGAGAG***TGA***aggaaattgttgttacgaaagtcagtgattatgtattgtgtagtatagta

Start and stop codons are highlighted for gene coding for uncharacterized protein YBL113C.

Protein

MDLNQRKEKKGQHVGCCGSRTDLSADTVELIERMDRLAENQATASMSIVALPSSFQESNSSDRCRKYCSS
DEDSDTCIHGSANASTNATTNSSTNATTTASINVRTSATTTASINVRTSATTTESTNSNTNATTTESTNS
STNATTTASINVRTSATTTESTNSSTNATTTASINVRTSATTTESTNSSTNATTTASINVRTSATTTEST
NSNTNASTNATTNSSTNATTTASTNVRTSATTNATTNSSTNATTTASTNVRTSATTTASTNVRTSATTTA
SINVRTSATTTESINSSTNATTTESTNSNTSATTTESTDSNTNATTTASINVRTSATTTESTNSNTSATT
TESTDSNTSATTTASTNSSTNATTTASTNSSTNATTTESTNASAKEDANKDGNAEDNRFHPVTDINKESY
KRKGSQMVFLERKKLKAQFPNTSENMNVLQFLGFRSDEIKHLFLYGIDIYFCPEGVFTQYGLCKGCQKMF
GLCVCWAGQKVSYRRIAWEALAVERMLRNDEEYKEYLEDIEPYHGDPVGYLKYFSVKRREIYSQIQRNYA
WYLAITRRRETISVLDSTRGKQGSQVFRMSGRQIKELYYKVWSNLRESKTEVLQYFLNWDEKKCREEWEA
KDDTVFVEALEKVGVFQRLRSMTSAGLQGPQYVKLQFSRHHRQLRSRYELSLGMHLRDQLALGVTPSKVP
HWTAFLSMLIGLFYNKTFRQKLEYLLEQISEMWLLPHWLDLANVEVLAADNTRVPLYMLMVAVHKELDSD
DVPDGRFDIILLCRDSSREVGE

Genome annotation

- *De novo*, e.g. SNAP, Genscan
  - Examining statistical properties of DNA sequence.
- Homology-based, e.g. GeneWise (Wise2).
- Combined *de novo* and homology-based, e.g. Ensembl genome database.
- Manual
  - Simple: based on BLAST, e.g. TBLASTX.
  - Complete: integration of *de novo* and homology-based genome annotation with BLAST results and other DNA sequence analyses.

Finding regulatory regions

- Functional motifs in DNA are often found with nonrandom frequency
  - E.g. protein-binding sites, regulatory regions (promoters, enhancers).
- Software exists to discover motifs, and to search sequences for motifs
  - E.g. MEME and MAST (also work on proteins).

Further reading

Bioinformatics textbooks on the reading list for the lecture in Week 1.
