# Supplementary material for: 4273π: Bioinformatics education on low cost ARM hardware
Source: BMC Bioinformatics. 2013 Aug 12;14:243. doi: 10.1186/1471-2105-14-243 (PMC3751261; doi:10.1186/1471-2105-14-243)
Supplement: Additional file 2 — 4273π Bioinformatics for Biologists teaching material, Version 1.01. The module handbook, lectures and practicals are included. The latest version, including Linux, software and BLAST databases, is available at the 4273π Web site [25]. [file 1471-2105-14-243-S2.zip › 4273pi_course_material/general/handbook.pdf]

# 4273π Bioinformatics for Biologists: Course Handbook

© 2013 D. Barker, D.E.K. Ferrier, P.W. Holland, J.B.O. Mitchell, H. Plaisier, M.G. Ritchie and S. D. Smart. This is an Open Access document distributed under the terms of the Creative Commons Attribution License (http://creativecommons.org/licenses/by/2.0), which permits unrestricted use, distribution, and reproduction in any medium, provided the original work is properly cited.

4273π, Version 1.01. http://eggg.st-andrews.ac.uk/4273pi

# CONTRIBUTORS TO 4273π BIOINFORMATICS FOR BIOLOGISTS

Dr Daniel Barker*,
School of Biology, University of St Andrews.

Dr David E.K. Ferrier,
School of Biology, University of St Andrews.

Professor Peter W.H. Holland,
Department of Zoology, University of Oxford.

Dr John B.O. Mitchell,
School of Chemistry, University of St Andrews.

Dr Heleen Plaisier,
School of Biology, University of St Andrews.

Professor Michael G. Ritchie,
School of Biology, University of St Andrews.

Mr Steven D. Smart,
School of Biology, University of St Andrews.

# LEARNING OBJECTIVES

1. A basic understanding of bioinformatics research techniques.

2. Appreciation of how bioinformatics techniques may be applied to biological research.

* Address for correspondence: Daniel Barker, Sir Harold Mitchell Building, School of Biology, University of St Andrews, Fife, KY16 9TH, UK. Email db60@st-andrews.ac.uk

## TIMETABLE

The lectures (and the seminar) are one hour long. All practicals are two hours long.

### *Week 1*

LECTURE: Genomes, sequences and bioinformatics data.
PRACTICAL: Linux and Perl.

### *Week 2*

PRACTICAL: Linux, Perl and protein BLAST.

### *Week 3*

PRACTICAL: Linux, Perl and delimiting gene/protein families.

### *Week 4*

LECTURE: Multiple alignment and phylogeny.
PRACTICAL: Multiple alignment and phylogeny.

### *Week 5*

LECTURE: Gene family evolution.
PRACTICAL: Gene family evolution.

### *Week 6*

LECTURE: BLAST; DNA sequence analysis.
PRACTICAL: DNA sequence analysis.

### *Week 7*

LECTURE: Looking at species differences.
PRACTICAL: Detecting positive selection.

### *Week 8*

LECTURE: Function and evolution of enzymes.
PRACTICAL: Function and evolution of enzymes.

### *Week 9*

Seminar (student presentations).

# RECOMMENDED READING

The main recommended books are listed during the first lecture. Other appropriate reading is recommended during the course.

# CONTINUOUS ASSESSMENT

### *Format*

Continuous assessment consists of one practical project, to be completed and written up in your own time. The continuous assessment **must be performed on your own**.

### *Mark Allocation*

Your final grade is based on: continuous assessment 50%, exam 50%.

### *Research*

The practical project requires use of OrthoMCL, which is introduced in the practical in Week 3. It is possible to make a start before Week 3, for example by downloading genome data, performing preliminary BLAST analyses and searching the literature.

Keep all files involved in the research (input data, output, any intermediate files, and scripts) within a single directory on the Raspberry Pi. Within that directory, make a text file named 'README.TXT', containing a brief guide to what each file is. This can just be a line or two for each file. (Intermediate files which are generated only as part of the operation of BLAST or OrthoMCL do not have to be documented.)

You will be assigned two species for your own analyses, from the list given below. For the purposes of these instructions, we will refer to them as Species A and Species B.

Download the genome-wide protein sets for Species A and Species B from the integr8 database (http://www.ebi.ac.uk/integr8). For both species, find the species in integr8, click 'downloads', and download:

- the complete proteome in Fasta format;
- InterPro hits (see Mulder et al. 2007, *Nucleic Acids Research*, 35, D224-D228); and
- GO annotations (see Gene Ontology Consortium 2000, *Nature Genetics*, 25, 25-29).

To search for orthologues between the two proteomes, use OrthoMCL on BLAST results, making sure to run BLAST with the BLOSUM45 substitution matrix. (Run blastall without command-line options to see how to select the substitution matrix.)

Write your own Perl script to process the OrthoMCL output to summarize differences in protein content between Species A and Species B. Summarize counts of proteins which are:

- present in a single copy in both species;
- unique to Species A;
- unique to Species B;
- present in one copy in Species A but more than one copy in Species B;
- present in one copy in Species B but more than one copy in Species A;
- present in more than one copy in each species.

The Perl script is an important part of your work and will be examined for authenticity and correct function during marking. Even if the summary could be obtained by other means (e.g. manual counting), we wish to see this Perl script. (You can use e.g. manual counts to verify it is working). It is fine to use pieces of Perl or libraries found e.g. online, but you **must** indicate the source using a comment in the Perl code.

Where counts are notable, relate them to:

- the phenotypic characteristics of the two species, as revealed by literature searches;
- the nature of the proteins involved, as revealed by their InterPro hits and/or GO annotations; and
- the scientific literature where appropriate.

If you would find it instructive to perform and report on additional analyses, please do so.

### Write-Up

Write up your work as if it were to be submitted to a scientific journal. This will require further reading of background literature, relation of your work to the published work of others, concise but clear presentation of methods so that they are entirely repeatable, and discussion of results including any suggestions of further work that may prove valuable.

Figures and tables are often the 'heart' of a scientific paper. I suggest you keep notes of your research as it progresses, then prepare figures and tables, then begin the write-up on the basis of the figures, tables and your notes. It is often easiest to write the abstract last, since this summarizes the rest of the paper.

In preparing your write-up, follow the Instructions for Authors for submitting a research paper to the journal *Genome Biology*, at:

http://genomebiology.com/authors/instructions/research

However, please limit the total word count (including main text, reference list and captions, but not words within figures or tables) to a **strict maximum of 2000 words**. Limit the **total number of figures and tables to a maximum of three** (e.g. one table, two figures; two figures, one table; three tables; or three figures). For the main text, use Times New Roman 11 point (or a similar font), with 1.5 line spacing. Headings may be in a larger and/or different font.

### Handing In

Submit your work as files. These must be as follows:

- A zip file, which must be a zipped copy of the directory containing all your research files, not forgetting the 'README.TXT' file providing a key to the others; and
- A single PDF file, containing your write-up, in the format outlined above.

Hand-written notes made for your own records during the research should not be handed in.

You must keep at least one complete, identical copy of the work you have handed in. As ever, for the purposes of backup, I strongly recommend that you keep two copies.

### *Seminar*

Each student will present his or her practical project, in a short (5-10 minute) presentation, at the seminar in Week 10. The presentation takes place after handing in the continuous assessment. The presentation itself is not assessed. Giving and attending these presentations will be useful preparation for the exam.

### *Marking Scheme*

If your research files are complete, organized, original and authentic, your mark will mostly depend on the quality and content of the write-up.

### *Pairs of species for the practical project*

**Student 1**
Species A: *Bacillus anthracis* (strain Ames ancestor).
Species B: *Alkaliphilus oremlandii* (strain OhILAs).

**Student 2**
Species A: *Deinococcus radiodurans* (strain LMG 4051 / IFO 15346 / DSM 20539 / R1 / NCIB 9279 / ATCC 13939).
Species B: *Thermus thermophilus* (strain HB27 / ATCC BAA-163 / DSM 7039).

**Student 3**
Species A: *Chlamydia trachomatis* (strain A/HAR-13 / ATCC VR-571B).
Species B: *Protochlamydia amoebophila* (strain UWE25).

**Student 4**
Species A: *Thermoplasma volcanium* (strain DSM 4299 / GSS1 / ATCC 51530 / JCM 9571 / IFO 15438).
Species B: *Picrophilus torridus* (strain NBRC 100828 / JCM 10055 / DSM 9790 / ATCC 700027).

**Student 5**
Species A: *Chlorobaculum parvum* (subsp. *thiosulfatophilum*, strain DSM 263 / NCIB 8327 / NCIB 8327).
Species B: *Pelodictyon phaeoclathratiforme* (strain DSM 5477 / BU-1).

**Student 6**
Species A: *Nanoarchaeum equitans* (strain Kin4-M).
Species B: *Ignisphaera aggregans* (strain DSM 17230 / JCM 13409 / AQ1.S1).

**Student 7**
Species A: *Escherichia coli* 83972.
Species B: *Escherichia fergusonii* B253.

**Student 8**
Species A: *Rhodobacter sphaeroides* (strain ATCC 17023 / 2.4.1 / NCIB 8253 / DSM 158).
Species B: *Rhizobium meliloti* (strain 1021).

# EXAM

The exam lasts two hours. Please bring a calculator.

The exam consists of four questions, all of which are compulsory. Questions are broadly of a ‘problem-solving’ kind. There are 20 marks for each question and questions are weighted equally. Marks allocated to each part of each question are indicated.

Any subject from the lectures and practicals may appear. A thorough practical and theoretical understanding of the material covered will help. However, an in-depth knowledge of the details of Perl programming will not be required in the exam. If you have used Perl successfully as required for the practical-project, you should have sufficient knowledge of Perl for the exam, and can focus your revision on other aspects of the course.

To assist with revision, one ‘mock’ question is provided, below. Although this is intended to be helpful, it has not been subject to the same level of checking that would be applied to an actual exam question.

### *Mock Exam Question: Sequence alignment and phylogeny*

A multiple alignment of orthologous proteins for 20 species of eukaryote and one of archaea is to be used for phylogeny reconstruction. The multiple alignment is submitted to the phylogenetic model selection program, Modelgenerator. Part of Modelgenerator’s output is as follows:

```
****Bayesian Information Criterion (BIC)****

Model Selected: LG+I+G+F
-lnL = 226052.80535126594
k = 60 (Branch lengths included as model parameters)
BIC = 452680.14719995187

Substitution Model (with Rate Distribution):
Model of substitution: LG (Le and Gascuel, MBE 2008 25:1307-20)

Amino acid frequencies:
pi(A) = 0.06499
pi(R) = 0.05272
pi(N) = 0.04510
pi(D) = 0.06103
pi(C) = 0.01571
pi(Q) = 0.03788
pi(E) = 0.08217
pi(G) = 0.05936
pi(H) = 0.02181
pi(I) = 0.06304
pi(L) = 0.09289
pi(K) = 0.07879
pi(M) = 0.02303
pi(F) = 0.04061
pi(P) = 0.04163
pi(S) = 0.06028
pi(T) = 0.04958
pi(W) = 0.00801
pi(Y) = 0.03123
pi(V) = 0.07017


Model of rate heterogeneity: Discrete Gamma + Invariable sites
Number of rate categories: 1 + 4
Gamma distribution parameter alpha: 0.89
Proportion of invariable sites: 0.04


Relative rates and their probabilities:

    Rate      Probability
1   0.00000   0.04453
2   0.12499   0.23887
3   0.49881   0.23887
4   1.10482   0.23887
5   2.45778   0.23887
```

(a) Which phylogenetic model has been selected by the Bayesian Information Criterion (BIC)? Which substitution matrix and other features appear in this model? **[6 marks]**

(b) If we wish to reconstruct the phylogeny not from the protein sequences used in (a), but from the DNA sequences which code for them, outline the necessary steps to obtain a multiple alignment and select a phylogenetic model. **[4 marks]**

(c) The globin protein superfamily is present in all domains of life. Three-dimensional protein structures of globins are reasonably well-conserved, but their protein sequences are more variable. A researcher wishes to gather a representative sample of globins from the public sequence database. These will be used for phylogeny reconstruction, in a study of gene duplication, loss and divergence over evolutionary time. To find globin sequences, the researcher uses several known globin sequences as queries in searches of the protein database at the National

Center for Biotechnology Information (NCBI). Why might PSI-BLAST be helpful here? **[4 marks]**

(d) Using sketches to illustrate your answer, compare global and local pairwise sequence alignment. **[6 marks]**
