# Supplementary material for: 4273π: Bioinformatics education on low cost ARM hardware
Source: BMC Bioinformatics. 2013 Aug 12;14:243. doi: 10.1186/1471-2105-14-243 (PMC3751261; doi:10.1186/1471-2105-14-243)
Supplement: Additional file 2 — 4273π Bioinformatics for Biologists teaching material, Version 1.01. The module handbook, lectures and practicals are included. The latest version, including Linux, software and BLAST databases, is available at the 4273π Web site [25]. [file 1471-2105-14-243-S2.zip › 4273pi_course_material/week4/lecture_multalign_phylogeny.pdf]

Multiple Alignment and Phylogeny

4273π Bioinformatics for Biologists

Lecture, Week 4

Daniel Barker, School of Biology, University of St Andrews
Email db60@st-andrews.ac.uk

© 2013 D. Barker. This is an Open Access document distributed under the terms of the Creative Commons Attribution License (http://creativecommons.org/licenses/by/2.0), which permits unrestricted use, distribution, and reproduction in any medium, provided the original work is properly cited.

4273π, Version 1.01. http://eggg.st-andrews.ac.uk/4273pi

Outline of Lecture

- A ‘protocol’ to reconstruct phylogeny from sequences, with some discussion (but not too much) …
  - Steps involved.
  - Outline of theory.
  - Recommended software.
    - Non-definitive. Plenty of other software may be used, at every stage.
  - Additional material
    - Make sure to look through this, even if there isn’t time to cover it in the lecture.

What’s involved?

**Step:**	**Human difficulty:**	**Comput. difficulty:**
1. Gather sequences	*High*	Low
2. Make a multiple alignment	Low	Low
3. Choose a model of evolution	Low	Low
4. Phylogeny reconstruction	*Medium*	*High*
5. Visualization	Low	Low
6. Root the phylogeny	Low	Low

Step 1. *Gather sequences*

- When seeking homologues of protein-coding sequences, perform the search **at the protein level**
    - e.g. BLASTP, TBLASTN, BLASTX, TBLASTX.

**Program**	**Query**	**Database**	**Comparison**
**BLASTP**	**protein**	**protein**	**protein**
**TBLASTN**	**protein**	**nucleotide**	**protein**
**BLASTX**	**nucleotide**	**protein**	**protein**
**TBLASTX**	**nucleotide**	**nucleotide**	**protein**
BLASTN	nucleotide	nucleotide	nucleotide

- For non-coding sequences (e.g. rDNA, introns), there is no meaningful ‘protein level’.
    - Perform the search at the nucleotide level (e.g. BLASTN).

Multiple alignment

- Most functionally constrained sites tend to be more conserved across species
  - Protein function
- Sequences more closely related tend to share mutations
  - Phylogeny

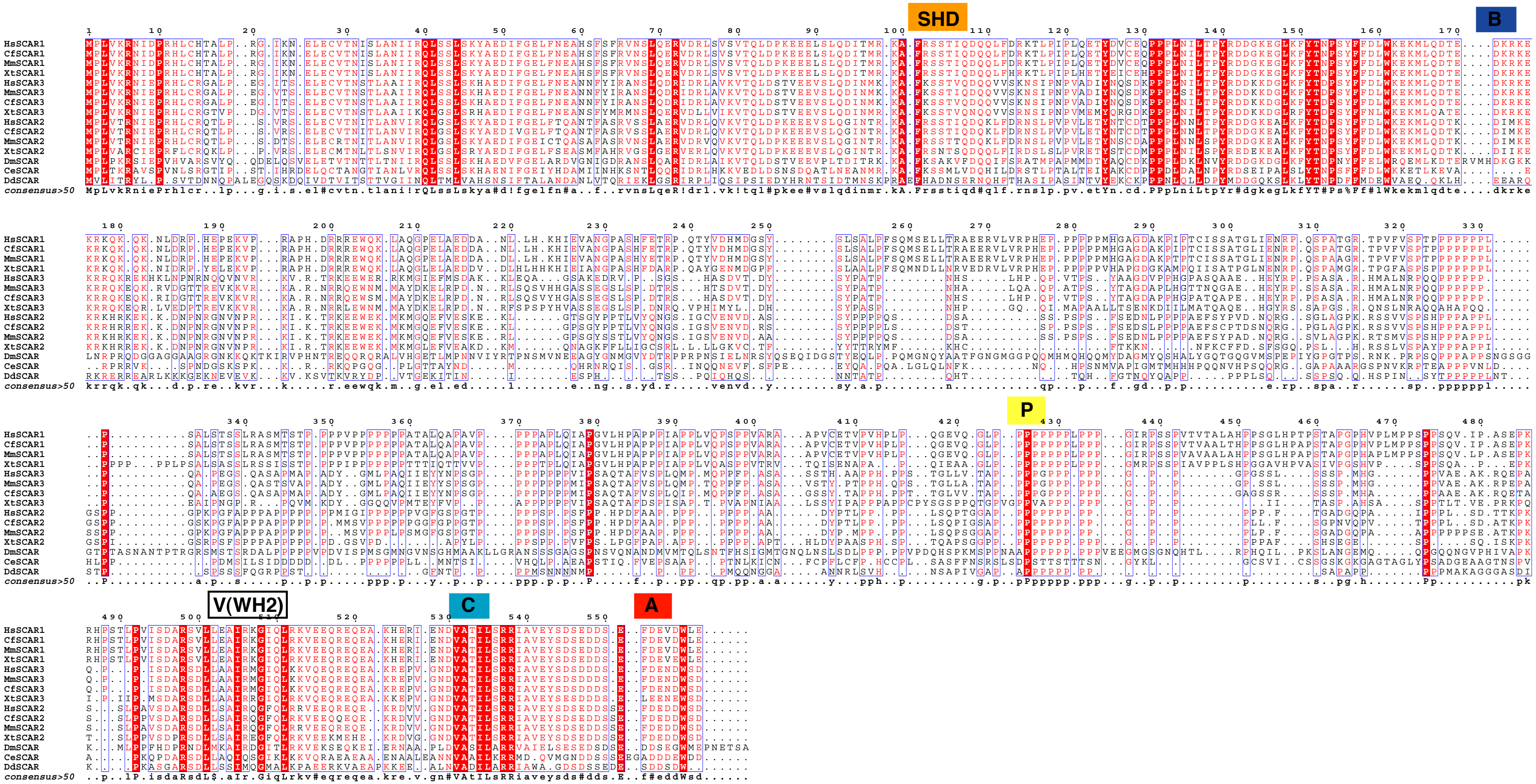

Source: Linardopoulou E.V., Parghi S.S., Friedman C., Osborn G.E., Parkhurst S.M. *et al*. (2007) *PLoS Genetics* 3: e237. Supplementary Figure S9.

Accumulated mutations provide evidence of evolutionary relationships

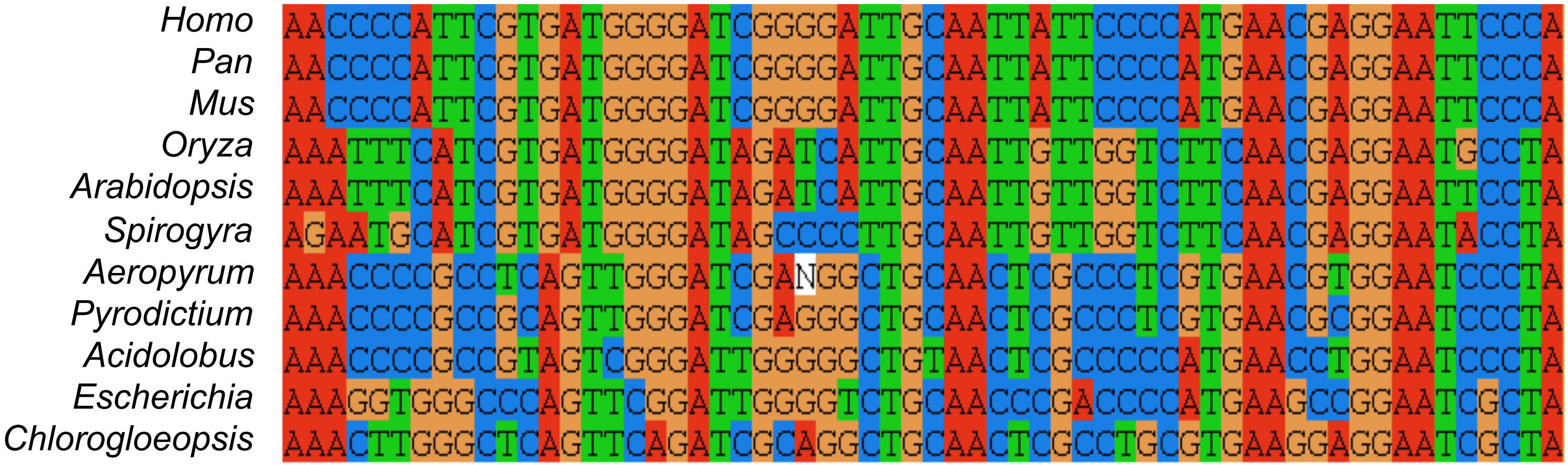

Multiple alignment of part of the SSU ribosomal RNA gene, from 11 organisms.

Sequences from GenBank.

Step 2. *Make a multiple alignment*

- MAFFT software.
  - ‘Cleaner’, more realistic indels than Clustal-X.
- Or, make or edit the multiple alignment manually
  - if you know what you’re doing (e.g. JalView software).
- For coding sequence, (± always) make the multiple alignment at the protein level.
  - If you want a nucleotide multiple alignment, get it from the protein multiple alignment and corresponding unaligned nucleotides (e.g. EMBOSS tranalign).
- For noncoding sequence, align nucleotides directly.

Running MAFFT

Mode	Suitable for
E-INS-i	sequences with any number of conserved domains, with less conserved sequences in between.
L-INS-i	sequences with one conserved domain and less conserved flanks.
G-INS-i	sequences with ± equal conservation along their whole length.

See: http://mafft.cbrc.jp/alignment/software/algorithms/algorithms.html

- Typical method of use (E-INS-i):

mafft --genafpair --maxiterate 10000 *input.fasta* > *output.fasta*

Running EMBOSS tranalign

- Sequences must be in same order in both input files (protein multiple alignment and unaligned nucleotides).
- Algorithm: ‘The protein sequences will typically include gap (-) characters. These are ignored during sequence comparison but replaced by --- in the nucleotide sequence alignment output.’

- Typical method of use, for sequences using ‘standard’ genetic code:

tranalign *unaligned_nucs.fasta aligned_prots.fasta*

- 23 different genetic codes are allowed, but you have to ask for them.
    - -table option.
    - E.g. invertebrate mitochondrial (EMBOSS 6.2):

    tranalign -table 5 *unaligned_nucs.fasta aligned_prots.fasta*

http://emboss.sourceforge.net

Step 3. *Choose a model of evolution*

- Many models of protein substitution exist.
  - A symmetrical matrix of substitution rates fixed *a priori.*
  - Plus, frequencies of each amino acid (either fixed *a priori*, or estimated in some way).
- Many models of nucleotide subsitution exist.
  - A symmetrical matrix of substitution rates, either estimated from your data, or with some parameters constrained to equal each other or a constant.
  - Plus, frequencies of each nucleotide (fixed *a priori* or estimated in some way).
- Use software to choose a model, e.g. MODELGENERATOR.
  - Use the Bayesian Information Criterion (BIC).

Which model of subsitution?

- In the GTR model, substitutions are changes between the four nucleotides (T, C, A and G).
- How about a simpler model, only paying attention to *transitions* and *transversions*?
- E.g. 'K80':

  Two rate parameters,

  α and β

  Frequencies fixed *a priori:*

  $\pi_T = \pi_C = \pi_A = \pi_G = 0.25$

From:	***To:***			
	**T**	**C**	**A**	**G**
**T**	-	α	β	β
**C**	α	-	β	β
**A**	β	β	-	α
**G**	β	β	α	-

Redrawn from Yang (2006, Table 1.1)

Rate heterogeneity

- Rates may vary at different columns in the multiple alignment
  - Conserved domains and active sites *vs* unconserved ‘linker regions’.
  - In coding nucleotide sequence, variation between codon positions.
  - In eukaryotic genes, introns *vs* exons. Etc.
- ‘Gamma’ models (+G or +Γ)
  - Allow several different rates of evolution (typically, 4).
  - Often recommended by model selection software. Fine.
- Invariant sites models (+I)
  - Often recommended by model selection software. Then, I suppose we must use ‘invariant sites’. (But +I models remain disturbing!)

Residue frequencies

- In a model like GTR, there are four different probabilities (for the four DNA bases).
  - Three parameters.
- These may be **estimated** by ML along with tree topology, branch lengths and rate parameters.
- Or: base probabilities may be estimated before phylogeny reconstruction proper, from the multiple alignment, and fixed at these **empirical** values.
  - This reduces the number of free parameters in the phylogeny likelihood maximization problem, by three.
  - I'm not sure what's best. (Maybe **empirical** as opposed to **estimated**?)

Format conversion

- Multiple sequence alignments may be read/ saved in different formats using Clustal-X.
- Or, convert formats using EMBOSS seqret, e.g.

seqret -auto -sequence fasta::*prots_aligned.fasta* -outseq phylip::*prots_aligned.phy*

- PHYLIP-format multiple alignments may still require editing to work with all software.
    - For PhyML, ensure the final character of every name is a space.

Step 4. *Phylogeny reconstruction*

- Given your data (multiple alignment), and the structure of your model of evolution, maximize the likelihood of the hypothesis (phylogenetic tree and the parameters of the model).
    - ML phylogeny reconstruction (PhyML or TREEFINDER).
- A heuristic search is used.
- Brief guide to heuristic searches in phylogeny:
    - NNI: very quick, unreliable.
    - SPR: moderately quick, moderately reliable.
    - TBR: very slow, more reliable.
    - PhyML 3.0 uses NNI by default, but can be made to use *both* NNI and SPR (recommended).

Support

- Conclusions based on a phylogenetic tree are dependent on its correctness.
- What about trees which are *almost* as likely as the ML tree?
- Bootstrap. It has been argued that bootstrap support values might be estimates of the following (note, these are all incompatible with each other):
    - *p*-values for tests of clade existence vs non-existence (p = 1 – bootstrap support value).
    - The probability of getting the same clade, if you did not use your sequences, but other sequences in the same gene family, evolving according to the same model.
    - (Some kind of posterior probability.)
    - See Felsenstein (2004, Chapter 20); Alfaro *et al*. (2003).

Step 5. *Visualize the phylogeny*

- Software: Mesquite is OK.
- Almost all phylogeny reconstruction software (PAML, MrBayes, etc.) gives an **unrooted** tree.
    - Any apparent root position, when you first load the tree into Mesquite, is **arbitrary** – **not** a result of any interest.
    - Do not draw any conclusions over the apparent root position!
    - It's up to you to assume a sensible root position, and impose it in a separate step …

Step 6. *Root the phylogeny*

- The phylogeny reconstruction software does not tell us where, in evolutionary time, the tree ‘began’.
- Outgroup rooting:
    - Include sequences homologous to the sequences of interest (ingroup), but ***known*** to be outside their clade.
    - Impose a root between the outgroup and the ingroup.
    - The ideal outgroup would be sister group to the ingroup.
    - The nightmare outgroup would be, if our knowledge was wrong, and we accidentally chose something that belongs in the ingroup.
    - Software: as for visualization (e.g. Mesquite).

Tree files

- Phylogeny reconstruction software outputs trees as text files.
- The tree is represented as bracketed text.
    - Newick (Phylip) format – rather plain.
    - Nexus format – same principle, but more bells & whistles (or the full kitchen sink).
- Visualisation software turns these into graphics (e.g. Mesquite).
- If things start going wrong which might relate to file format, take a look at the tree file in a text editor.

If it takes too much computer time

- Reconstructing phylogeny by maximum likelihood may be slow
  - for very large multiple alignments, or for large numbers of analyses.
- Can use ‘tricks’ to speed things up
  - – at increased risk of failing to obtain the true ML topology.
  - – E.g. do not estimate the free parameters of the model of evolution simultaneously with the phylogeny; instead, fix the them to the rough estimates which MODELGENERATOR supplied.

Faster methods

- Slightly faster: parsimony (e.g. in PAUP software).
- Much faster, and often not bad: neighbor joining (e.g. in BIONJ, QuickTree, PHYLIP or PAUP software).
- Fast, but ***horribly obsolete*** for phylogeny reconstruction: UPGMA.
    - Use Neighbor joining instead.

Bayesian methods

- Uses the same subsitution models as ML phylogeny reconstruction. E.g. MrBayes software.
- In constrast to ML, there is a 'built-in' indication of support
  - Posterior probabilities.
  - Depend on prior probabilities on topologies, branch lengths, and parameters of model of evolution (as well as multiple alignment and model).
- Bayesian phylogeny reconstruction is not rapid.
  - Samples the posterior distribution of phylogenetic trees.
  - Poor-quality samples are available almost immediately. Better samples take longer to obtain.
  - **Software defaults are not always appropriate.**

Web

- Joe Felsenstein’s list of phylogeny programs, http://evolution.genetics.washington.edu/phylip/software.html

Reading

- Alfaro, M.E., Zoller, S. and Lutzoni, F. (2003) Bayes or bootstrap? A simulation study comparing the performance of Bayesian Markov chain Monte Carlo sampling and bootstrapping in assessing phylogenetic confidence. *Molecular Biology and Evolution* 20: 255-266.
- Felsenstein, J. (2004) *Inferring Phylogenies* (Sunderland, Massachusetts: Sinauer).
- **Foster, P.G. (2007) Inferring phylogenetic relationships from sequence data. In P.H. Dear, editor, *Bioinformatics* (Bloxham, Oxfordshire: Scion), pp. 265-282.**
- Page, R.D.M. and Holmes, E.C. (1998) *Molecular Evolution: A Phylogenetic Approach* (Oxford: Blackwell).
- Yang, Z. (2006) *Computational Molecular Evolution* (Oxford: Oxford University Press).

ADDITIONAL MATERIAL FOLLOWS

Modelling nucleotide evolution: GTR

- The ‘general time-reversible’ model is often used, also called GTR or ‘6st’.
- Six parameters, *a*, *b*, *c*, *d* and *e*, representing rate of change between nucleotides
  - one rate of change between each nucleotide and each other nucleotide
  - symmetrical (time reversible) model, so, for example, rate from A to T is same as rate from T to A.
- Three parameters for nucleotide probabilities, π_T, π_C, π_A and π_G, where $\pi_T + \pi_C + \pi_A + \pi_G = 1$.

From:	To:			
	T	C	A	G
T	-	$a\pi_C$	$b\pi_A$	$c\pi_G$
C	$a\pi_T$	-	$d\pi_A$	$e\pi_G$
A	$b\pi_T$	$d\pi_C$	-	$f\pi_G$
G	$c\pi_T$	$e\pi_C$	$f\pi_A$	-

Redrawn from Yang (2006, Table 1.1).

Reconstructing phylogeny

- In the ML framework, we choose a *model*. Values for parameters of the model represent a *hypothesis*. We find the hypothesis which maximizes likelihood of our observed data, i.e. maximizes $L(H|D)$, where

  $L(H|D) = k\,P(D|H)$.
- In phylogeny, conventionally, our *model* consists of a substitution model (e.g. GTR) and a bifurcating tree with varying branch lengths.
- A hypothesis then consists of particular values of the parameters of the substitution model, a particular tree topology, and a set of branch lengths on that topology.

- Or the simplest nucleotide substitution model of all, JC69, which has only one parameter:

From:	*To:*			
	**T**	**C**	**A**	**G**
**T**	-	α	α	α
**C**	α	-	α	α
**A**	α	α	-	α
**G**	α	α	α	-

$\pi_T = \pi_C = \pi_A = \pi_G = 0.25$

- Several other nucleotide models are in common use
  - see Yang (2006, Chapter 1).

Automated model selection

- Choosing the substitution model that maximizes likelihood is not appropriate.
  - Models with more free parameters always explain the data better, i.e. have a higher likelihood.
  - E.g. there is *no way* that K80 can give a lower likelihood than JC69; and *no way* that GTR can give a lower likelihood than K80.
- If models are hierarchical, we could use a likelihood ratio test to choose between them.
  - E.g. JC69 is a special case of K80, in which the transition rate is fixed at the same value as the transversion rate.
- But, we're not *really* interested in the substitution models as hypotheses themselves.

Bayesian information criterion

- We want the most complex model for which we have a hope that we can estimate the parameters fairly well.
- (1) Generate a 'quick and dirty' tree topology, e.g. using NJ. Keep the topology fixed.
- (2) Maximize the tree's likelihood by varying branch lengths and the free parameters of the substitution model, for each of a range of substitution models.
- (3) Then, for the 'proper' phylogeny use the model with the lowest Bayesian Information Criterion, BIC, where

  $\mathrm{BIC} = -2 \ln L + p \ln(n)$.

  $\ln L$ is the log likelihood of the phylogeny, p is the number of free parameters in the model, and n is the number of sites in the multiple alignment.

- May use a criterion other than BIC.
  - Likelihood ratio tests are possible, but not widely used in this context.
  - Akaike information content, AIC, is a popular alternative to BIC.
  - BIC, LRTs and two versions of AIC are available in Modelgenerator software.
- With AIC or LRTs, longer multiple alignments will tend to favour complex models. BIC attempts to address this.
  - With BIC, with a longer multiple alignment a complex model has to show a greater increase in likelihood before it is preferred over a simpler model.

Variable rates among sites

- We know, in most sequences, some sites will evolve faster than others. This is not reflected in the substitution models discussed so far.
- ‘Gamma’ site heterogeneity addresses this. The substitution matrix is as before (e.g. GTR, K80, JC69). However, the relative rate at different sites is allowed to vary. These models are called ‘+Γ’ (or ‘+G’), e.g. GTR+Γ, K80+Γ, JC69+Γ.
- Adding ‘Γ’ adds a parameter to the model.
  - Assess ‘+Γ’ models, along with others, during model selection.
  - Use a ‘+Γ’ model if it has lowest BIC.

- It is possible to assume some sites are invariant, i.e. never change.
- Such models are ‘+I’. Often, the approach is used in combination with ‘+Γ’, e.g. GTR+I+Γ, K80+I+Γ, JC69+I+Γ.

- (**But, think about this**: *Is there much reason to use ‘+I’, when ‘+Γ’ already allows rates to vary?*)

Bayesian Methods

- Unlike likelihood methods, for a given model structure, Bayesian methods assign a probability to every conceivable hypothesis.
- This relies on *prior probabilities* for all parameters.
- Prior probabilities may be ‘made up’, or subjective. This is controversial.
- Or, prior probabilities may be ‘learnt’ empirically, from ‘training data’. This is uncontroversial.
- Bayesian methods are useful for tasks of automatic classification.
  - I.e. we have a few known examples of each class. We will use their features to ‘learn’ the nature of each class.
  - This suits many problems in bioinformatics, but, arguably, perhaps not phylogeny reconstruction.

Bayesian phylogeny reconstruction

- Given prior probability distributions for tree topology, branch lengths, and the parameters of the substitution model, sample the universe of possible trees in proportion to their probability.
  - Bayesian Markov chain Monte Carlo (MCMC).
- This outputs not one tree, but a sample of trees in which features (e.g. topology) will be represented in proportion to their posterior probabilities.
- A clade with high posterior probability is well supported.

Bayesian difficulties

- Bayesian MCMC is a sampling procedure. It's easy to get a *poor* sample from the posterior distribution, very quickly. A representative sample takes more time and effort.
  - Just a practical difficulty, easily addressed.
- Priors. We may believe, *a priori*, that all tree topologies are equally probable. This sounds reasonable and is a 'flat prior on tree topology'.
- We may believe, *a priori*, that all clades are equally probable. This sounds reasonable and is a 'flat prior on clades'.
- These two priors are not the same!
  - A theoretical difficulty, less easily addressed.
  - Practical relevance of the difficulty is disputed.
  - See Yang (2006, Chapter 5);
    Brandley *et al*. (2005) *Systematic Biology* 55: 138-146.

Similarities between Bayesian and ML phylogeny reconstruction

- Both can use the same models of evolution (e.g. GTR, K80, JC69, '+Γ' and '+I' variants).
- The model selection procedure is, potentially, the same (or at least, addresses the same issues).
- Often, the ML tree topology is also highly probable, in which case Bayesian and ML approaches give similar answers.

Reasons to use Bayesian phylogeny reconstruction rather than ML

- **(1) The interpretation of Bayesian support values (posterior probabilities) is straightforward**.
  - In contrast to bootstrap support values on ML trees (with have at least three, conflicting interpretations), the *meaning* of posterior probabilities is clear and, in itself, uncontroversial.

- **(2) Complicated models of evolution are more easily implemented in a Bayesian framework.**
  - It is hard to maximize likelihood across tree topology, branch lengths, and values of the parameters of the substitution model, if the substitution model has a large number of parameters.
  - This is a practical reason to use Bayesian-MCMC instead of ML with models involving heterotachy, pattern-heterogeneity, etc.

Summary of Bayesian and ML problems

- **Theoretical (fundamental) problems**
  - **In ML phylogeny reconstruction, obtaining a measure of support for clades and other features.**
  - **In Bayesian phylogeny reconstruction, the choice of priors.**
- **Practical problems**
  - In ML phylogeny reconstruction, maximizing likelihood for very complex substitution models.
  - In Bayesian phylogeny reconstruction, getting a good sample of the posterior probability distribution.

A potential course of action …

- Use ML phylogeny reconstruction (except where the substitution model is too complex for this to be practical).
- Or, you can run both Bayesian-MCMC phylogeny reconstruction too, with the same substitution model. Present the ML tree, and label its nodes with both ML bootstrap support values and Bayesian posterior probabilities.
- Where these two kinds of support value broadly agree, the conclusion is more immune to criticism.
- No phylogeny will ever be totally immune to criticism! **Problems in taxon sampling, choice of gene, and sequence alignment affect all methods of phylogeny reconstruction.**
