# Supplementary material for: 4273π: Bioinformatics education on low cost ARM hardware
Source: BMC Bioinformatics. 2013 Aug 12;14:243. doi: 10.1186/1471-2105-14-243 (PMC3751261; doi:10.1186/1471-2105-14-243)
Supplement: Additional file 2 — 4273π Bioinformatics for Biologists teaching material, Version 1.01. The module handbook, lectures and practicals are included. The latest version, including Linux, software and BLAST databases, is available at the 4273π Web site [25]. [file 1471-2105-14-243-S2.zip › 4273pi_course_material/week4/practical_multalign_phylogeny.pdf]

4273π Bioinformatics for Biologists

Practical, Week 4: Multiple Alignment and Phylogeny

Daniel Barker, School of Biology, University of St Andrews
Email db60@st-andrews.ac.uk

© 2013 D. Barker. This is an Open Access document distributed under the terms of the Creative Commons Attribution License (http://creativecommons.org/licenses/by/2.0), which permits unrestricted use, distribution, and reproduction in any medium, provided the original work is properly cited.

4273π, Version 1.01. http://eggg.st-andrews.ac.uk/4273pi

The learning objective of this practical is to:

- gain practical experience of all aspects of phylogeny reconstruction, using a state-of-the-art protocol.

This practical session involves collating sequences for making a multiple alignment, making a multiple alignment, choosing a phylogenetic model, and reconstructing phylogeny according to that model. We'll use the forkhead gene family as a case study.

Our protocol is based on that of Foster (2007). However, compared to a 'research-grade' analysis, in this practical we will take certain shortcuts to save time. We will take a large shortcut at the step of gathering sequences from the database. For the protein family in our case study, it would be a major job to assemble a representative set of sequences across all species in which they occur. We will also take further shortcuts: we will omit consideration of site-heterogeneity models (Gamma models), and will also omit the step of obtaining branch support (bootstrap) values.

Install software

As in Week 2, firstly, update the list of packages your computer knows about:

```
sudo apt-get update
```

Then, use `apt-get` to install the following packages:

```
clustalx
mafft
phyml
```

These all install the similarly named program.

Modelgenerator and Mesquite may not be installed via the standard installation mechanism, but may be installed as follows.

Download the UNIX/Linux distribution of Mesquite, `mesquite.tgz`, from:

http://mesquiteproject.org/mesquite/download/download.html

Put the file in the `~/4273pi/week4` directory. Change to that directory. Then, unpack the software (this is all the 'installation' that has to be done):

```
tar xzf mesquite.tgz
```

Download Modelgenerator, `modelgenerator_v_851.zip`, from:

http://bioinf.may.ie/modelgenerator

Put the file in the `~/4273pi/week4` directory. Then, unzip the software:

```
unzip modelgenerator_v_851.zip
```

Finding initial sequences by a keyword search at the NCBI

One famous protein in the forkhead (fox) family is forkhead box protein P2 (FoxP2), encoded by the *FOXP2* gene. It has been argued that mutation in this gene is responsible for language in humans (Enard et al. 2002).

Go to the NCBI Web site:

http://www.ncbi.nlm.nih.gov

In the search box, enter the following:

```
foxp2 AND Homo sapiens[ORGN]
```

This searches for all sequence records that contain the text 'foxp2' and come from the organism *Homo sapiens*. When the search is done, click 'Protein'. There are a few dozen sequences – a larger number than the human genome actually contains. This is not surprising, because forkhead box proteins have been a major area of research. They have often been sequenced and re-sequenced. Let's narrow down our search, to find a human sequence from a high-quality protein database. Click the 'Limits' link and change 'Source database' to 'UniprotKB/Swiss-Prot'.

Display the sequence in Fasta format, then paste it into a text file on the Raspberry Pi. (Take care not to split the Fasta header onto two lines! That would lead to trouble later.)

Using BLAST or PSI-BLAST to find additional homologous sequences

Beginning with this initial sequence you found with the keyword search, use BLASTP and/or PSI-BLAST on the NCBI Web server to gather a range of forkhead box protein sequences, from a variety of organisms. BLAST may be run online here:

http://blast.ncbi.nlm.nih.gov/Blast.cgi

Click ‘protein blast’. You can then paste in your query, choose either the ‘blastp’ or ‘PSI-BLAST’ algorithm, and click ‘BLAST’.

Note on PSI-BLAST

PSI-BLAST begins with a BLASTP search. When you run a PSI-BLAST search, this is the first search reported. If you then scroll down to where the report says ‘Run PSI-Blast iteration 2’ and click ‘Go’, PSI-BLAST will use the best-matching proteins, found in this first search, to build a *profile*. This is a model of the proteins. A profile does not necessarily have a single amino acid at each position. Rather, amino acids are represented according to their frequency at each position. This better represents the diversity of a protein family, where the residue at a given site is not always perfectly conserved. For the second iteration, the profile (rather than the original query sequence) is used as the query for search. Several rounds of searching, adjusting the profile with sequences found and searching again, may be performed interactively on the NCBI Web server. The PSI-BLAST approach is not very sophisticated – for building a profile one should really use a multiple alignment, whereas PSI-BLAST just approximates a multiple alignment by using a series of pairwise alignments. Nevertheless, PSI-BLAST often works well with large protein families and a large database. PSI-BLAST is not suitable for small databases, e.g. databases containing proteins from only one genome (or a small number of genomes).

Using BLAST and/or PSI-BLAST, make sure to obtain at least one sequence from each of these taxonomic groups: chimpanzee, birds and fungi. On the NCBI BLAST Web form, you can specify the species, genus or higher taxon for BLAST to search, in the optional ‘Organism’ box under ‘Choose Search Set’. Some common names will work, but it is safest to use the formal (Latin) names. Suitable names can be found in the NCBI Taxonomy database:

http://www.ncbi.nlm.nih.gov/taxonomy

Alternatively, after a BLAST or PSI-BLAST search, click ‘Taxonomy reports’ near the top of the results page. This organises the sequences found (‘hits’) according to the species they come from.

Also obtain one more sequence from human, making sure it is not just an isoform of the human protein that you already have.

In all, gather five or six sequences into a single text file on the Raspberry Pi.

Multiple alignment with MAFFT, and viewing with Clustal-X

Now make a multiple alignment on the Raspberry Pi, using MAFFT (Katoh and Toh 2008). We will use MAFFT's 'E-INS-I' algorithm. Use this command, substituting appropriate file names for *input.fa* and *aligned.fa*:

`mafft --genafpair --maxiterate 10000` *input.fa* > *aligned.fa*

Take a look at the output file in `nedit`. It's a multiple alignment in Fasta format – not easy to interpret as it is. Start Clustal-X, in the 'Education' section under the 'Start' menu. Although we are not using Clustal to make the multiple alignment, you can still use it to open and view the multiple alignment made by MAFFT.

Question. Does it look like MAFFT did a good job of aligning your sequences?

Question. PSI-BLAST is most useful for finding divergent sequences. Looking at the multiple alignment, was PSI-BLAST appropriate for the search or was BLASTP likely to be adequate?

Converting the multiple alignment to PHYLIP format, with `seqret`

Data formats in phylogeny are still a problem – there are various different standards. PHYML, below, requires data in PHYLIP format. First, take a copy of the file containing your aligned sequences. Next, edit the file in `nedit` to give each sequence a name strictly *nine characters* long. Try and include the sequence accession and a summary of the species name within these nine characters! Use the underscore if you require spacing.

Now, convert the multiple alignment to PHYLIP format. This can be done using the program `seqret` in the EMBOSS package:

```
seqret -auto -sprotein1 -sequence fasta::aligned.fa -outseq phylip::aligned.phy
```

(Substitute the appropriate input file name for `aligned.fa` and the desired output file name for `aligned.phy`.) Take a look at the new, PHYLIP-format multiple alignment in `nedit`. It is still a text file, but its layout is different from that of the Fasta-format version.

Choosing a model for phylogeny reconstruction with Modelgenerator

A range of protein substitution matrices have been defined in the literature, based on analyses of large numbers of multiple alignments. One example is BLOSUM62 (the default used in BLAST searches), or the more recently developed JTT. The question is, when inferring your phylogeny by maximum likelihood (ML), which model is appropriate to use for your own particular multiple alignment? We want a model which explains the multiple alignment adequately, but without over-fitting. In other words, this is a problem of model selection, and the usual approaches (e.g. Akaike Information Criterion, AIC) may be applied.

Modelgenerator (Keane *et al.* 2006) builds a quick phylogenetic tree topology using neighbor-joining (NJ). Fixing the tree topology to that of the NJ tree, it then calculates the likelihood of the phylogeny according to each of a range of protein substitution models. The first part of the model is the substitution matrix (e.g. BLOSUM or JTT). If the model has '+F' (e.g. JTT+F), this means it estimates amino acid frequencies from the multiple alignment, rather than assuming the frequencies implied by the substitution matrix. If the model has '+G' (e.g. JTT+G), it allows rates of evolution to vary at different sites in the alignment – also known as a site heterogeneity model or Gamma (+Γ). '+I' (e.g. JTT+I) allows a class of sites to be invariant, i.e., to not evolve at all within the case study. '+F', '+G' and '+I' may be used in combination (e.g. JTT+I+G, JTT+I+F, JTT+G+F, JTT+I+G+F).

There are various ways to assess the most suitable substitution model. We will regard the best model as the one with the lowest Bayesian Information Criterion (BIC). We will then use this selected model to infer the phylogeny, 'properly', using separate phylogeny reconstruction software.

Modelgenerator is a Java program. If you are working in the `~/4273pi/week4` directory and you put `modelgenerator.jar` there, you can launch Modelgenerator with the command:

```
java -jar modelgenerator.jar
```

If working in another directory, give the path to `modelgenerator.jar`, not just the file name.

Normally, one would request four Gamma categories. This means that, when a model of evolution is applied which allows heterogeneity in evolutionary rates among sites, there will be four different rates. *To save time, we will request one Gamma category*. This means that evolutionary rate variation among sites will not be considered. This is not ideal, but allows Modelgenerator to run faster.

Modelgenerator can read either the Fasta-format or the PHYLIP-format multiple alignment file. It will write output to the screen and also to a text file. The first time you run Modelgenerator in the current directory, the output file will be named `modelgenerator0.out`. Subsequent runs will not delete this file, but will create new ones, named `modelgenerator1.out`, `modelgenerator2.out`, etc.

When Modelgenerator is complete, open its output file in `nedit`, maximise the window, and scroll through it carefully. The models selected by AIC (AIC1), a variant on the AIC criterion (AIC2) and BIC are reported. There is also a list of models, sorted in order of AIC1 from lowest ('best') AIC1 to highest. In some cases, Modelgenerator will report AICc, is a 'corrected' version of AIC suitable for small amounts of data.

Make a note of the model selected by the BIC criterion.

<u>Question</u>. If you have followed the instructions precisely, you will find all '+G' models have the same log likelihood (`Ln` column in the 'Model-fit Ranking') as the same model *without* +G. Why?

Reconstructing the phylogeny with PHYML

You are now ready to run the phylogeny reconstruction using PHYML (Guindon *et al.* 2010). Launch it as follows:

```
phyml
```

Interactively set up the analysis as recommended by Modelgenerator. The results will go into files with names ending in `.txt`, of which the file ending in `_tree.txt` is the phylogenetic tree in PHYLIP format.

PhyML presents a range of options as text in the terminal window. Make the window big, so that the text fits neatly. Read each 'page' of options very carefully. On the first page, for example, you will want to change 'Data type' from 'DNA' to 'AA' (amino acid). You can flick through the possible data types by pressing `d`, followed by RETURN. When you are satisfied that all options on the page are appropriate, enter + to move on to the next page. On the third and final menu, when all options have been set correctly enter `Y` to launch the analysis.

Other changes you should make are:

- set 'Model of amino-acids substitution' to the substitution matrix chosen by Modelgenerator;
- set 'Amino acid frequencies' to either 'empirical' (if Modelgenerator selected a +F model) or 'model' (otherwise);
- set 'One category of substitution rate?' to 'yes' (if a '+G' model was recommended by Modelgenerator) or 'no' (otherwise);
- if 'one category of substitution rate' is 'no' (i.e. if you *are* using a '+G' model), set 'number of substitution rate categories' to the number you asked Modelgenerator to consider;
- set 'Proportion of invariable sites (fixed/estimated) to 'estimated' (i.e. 'optimise p-invar') if Modelgenerator selected a '+I' model, or to 'fixed (p-invar = 0.00)' otherwise;
- set 'Tree topology search operations' to 'best of NNI and SPR', which will make PhyML run more slowly and more carefully; and

- set ‘Approximate likelihood ratio test’ to ‘no’.

Visualising and rooting the phylogenetic tree with Mesquite

Typical phylogeny reconstruction software, including PhyML, always outputs an unrooted tree. *If it looks rooted, the root position is just arbitrary*! To remedy this, you may set the position of the root, between the ingroup and the outgroup. The outgroup is a sequence or group of sequences that we ‘know’ lies outside the ingroup. In the current case, it is reasonable to use the fungal sequence as the outgroup. You can place the root between this and the animal sequences.

Start Mesquite. the command is `run_mesquite.sh` but this is not on your `PATH`, so you have to give its location (or change your `PATH` environment variable). If you installed Mesquite in the `~/4273pi/week4` directory, you may launch it as follows:

```
~/4273pi/week4/Mesquite_Folder/run_mesquite.sh
```

Mesquite has a graphical user interface. Once Mesquite has started (which takes a while), from its ‘File’ menu you can open the tree found by PhyML. This is in PHYLIP format. You can then ‘View Trees’ and re-root the tree using the ‘Reroot at branch’ tool.

Mesquite has many options. Experiment, for example with the ‘Branches proportional to Lengths’ option in the ‘Drawing’ menu.

Question. Does the topology of your phylogeny match expectations from previous knowledge and the literature on forkhead box proteins?

Extension: repeating the analysis with nucleotide sequences

Programs like MAFFT can make a multiple alignment of DNA sequences, using DNA sequences as input, but this is *only* recommended for non-coding sequence (e.g. introns or rRNA genes). For coding sequence, directly aligning the nucleotides is not usually a good idea. MAFFT may introduce indels whose length is not a multiple of three. For coding sequence, such indels imply a frameshift mutation, which is unlikely to be biologically correct. (This may be correct, but only very rarely. Frameshift mutations are likely to be disadvantageous, hence rapidly eliminated from the gene pool by natural selection.)

Using the accessions of the protein sequences you used to make the multiple alignment, return to the NCBI Web site and obtain the corresponding coding sequences. Use the EMBOSS program `tranalign` on the Raspberry Pi to convert the protein multiple alignment, and the unaligned coding sequences, into a nucleotide multiple alignment. EMBOSS is documented here:

http://emboss.sourceforge.net/apps

(see 'Stable release 6.4.0' under 'Application Documentation'). Submit this nucleotide multiple alignment to Modelgenerator to select a model, and use PhyML to reconstruct the phylogeny from the nucleotide multiple alignment using this model.

Question. Does the phylogeny reconstructed from nucleotides differ from the phylogeny reconstructed from proteins? Consider both the topology of the reconstructed phylogeny and its branch lengths. Which topology do you consider most likely to be correct?

References

Enard, W., Przeworski, M., Fisher, S.E., Lai, C.S.L., Wiebe, V., Kitano, T., Monaco, A.P. and Pääbo, S. (2002) Molecular evolution of *FOXP2*, a gene involved in speech and language. *Nature* 418:869-872.

Foster, P.G. (2007) Inferring phylogenetic relationships from sequence data. In P.H. Dear, editor, *Bioinformatics* (Bloxham, Oxfordshire: Scion), pp. 265-282.

Guindon S., Dufayard J.F., Lefort V., Anisimova M., Hordijk W. and Gascuel O. (2010) New algorithms and methods to estimate maximum-likelihood phylogenies: assessing the performance of PhyML 3.0. *Systematic Biology* 59:307-21.

Katoh, K. and Toh, H. (2008) Recent developments in the MAFFT multiple sequence alignment program. *Briefings in Bioinformatics* 9:286-298.

Keane, T.M., Creevey, C.J., Pentony, M.P., Naughton, T.J. and Mclnerney, J.O. (2006) Assessment of methods for amino acid matrix selection and their use on empirical data shows that ad hoc assumptions for choice of matrix are not justified. *BMC Evolutionary Biology* 6:29.
